# Effects of Microplastic Exposure on Human Digestive, Reproductive, and Respiratory Health: A Rapid Systematic Review

**DOI:** 10.1021/acs.est.3c09524

**Published:** 2024-12-18

**Authors:** Nicholas Chartres, Courtney B. Cooper, Garret Bland, Katherine E. Pelch, Sheiphali A. Gandhi, Abena BakenRa, Tracey J. Woodruff

**Affiliations:** † Program on Reproductive Health and the Environment, Department of Obstetrics, Gynecology and Reproductive Sciences, University of California, San Francisco, San Francisco, California 94143, United States; ‡ School of Pharmacy, Faculty of Medicine & Health, The University of Sydney, Sydney 2006, Australia; § Natural Resources Defense Council, San Francisco, California 94104, United States; ∥ Division of Occupational, Environmental, and Climate Medicine, Department of Medicine, University of California, San Francisco, San Francisco, California 94117, United States; ⊥ Division of Pulmonary, Critical Care, Allergy, and Sleep Medicine, Department of Medicine, University of California, San Francisco, San Francisco, California 94117, United States

**Keywords:** systematic review, microplastics, reproductive, digestive, respiratory, hazard assessment, toxicology, cancer

## Abstract

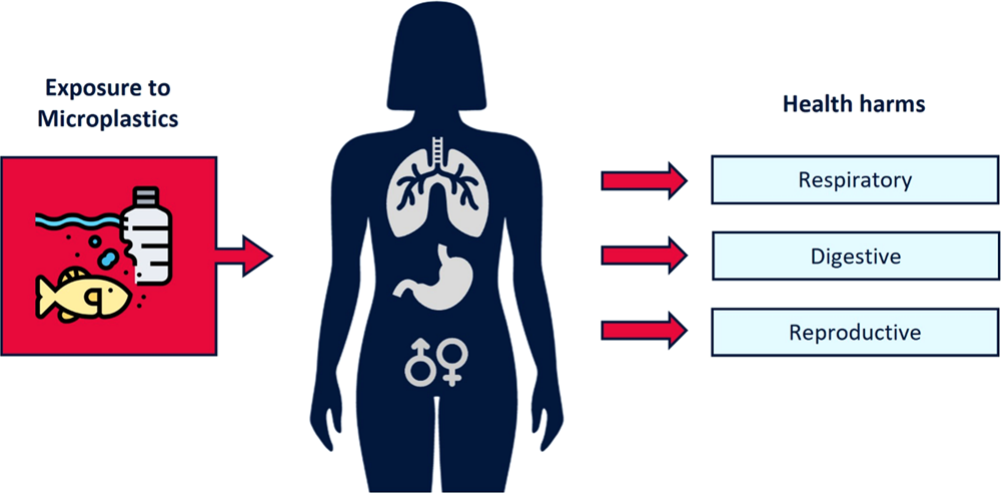

Microplastics are ubiquitous environmental contaminants for which there are documented human exposures, but there is a paucity of research evaluating their impacts on human health. We conducted a rapid systematic review using the “Navigation Guide” systematic review method. We searched four databases in July 2022 and April 2024 with no restriction on the date. We included studies using predefined eligibility criteria that quantitatively examined the association of microplastic exposure with any health outcomes. We amended the eligibility criteria after screening studies and prioritized digestive, reproductive, and respiratory outcomes for further evaluation. We included three human observational studies examining reproductive (*n* = 2) and respiratory (*n* = 1) outcomes and 28 animal studies examining reproductive (*n* = 11), respiratory (*n* = 7), and digestive (*n* = 10) outcomes. For reproductive outcomes (sperm quality) and digestive outcomes (immunosuppresion) we rated overall body evidence as “high” quality and concluded microplastic exposure is “suspected” to adversely impact them. For reproductive outcomes (female follicles and reproductive hormones), digestive outcomes (gross or microanatomic colon/small intestine effects, alters cell proliferation and cell death, and chronic inflammation), and respiratory outcomes (pulmonary function, lung injury, chronic inflammation, and oxidative stress) we rated the overall body of evidence as “moderate” quality and concluded microplastic exposure is “suspected” to adversely impact them. We concluded that exposure to microplastics is “unclassifiable” for birth outcomes and gestational age in humans on the basis of the “low” and “very low” quality of the evidence. We concluded that microplastics are “suspected” to harm human reproductive, digestive, and respiratory health, with a suggested link to colon and lung cancer. Future research on microplastics should investigate additional health outcomes impacted by microplastic exposure and identify strategies to reduce exposure.

## Introduction

In 2019, 460 million metric tons of plastic were produced,^1^ with estimates that production will triple by 2060.^1,2^ The largest proportion of plastic production comes from single-use plastics, and 98% of single-use plastics are derived from fossil fuels.^3^ Fossil fuels are used to make petrochemicals, a broad and diverse group of chemicals that are the feedstock for the production of plastics.^4^ The petrochemical industry is pivoting to ramp up the production of plastics given expectations that the sales of oil and gas will decrease.^5,6^ This has raised concern, as the production of plastics also contributes to greenhouse gases across their life cycle from cradle to grave.^3,7^ In addition, there is well-established evidence from authoritative or systematic reviews on the human health effects of plasticizers and plastics-related chemicals.^8^ For example, phthalates can increase the risk of preterm birth^9^ and adverse male reproductive effects^10^ and bisphenol A (BPA) exposure is likely or very likely to be a hazard for immunotoxicity, metabolic effects, neurotoxicity and developmental toxicity, female reproductive toxicity, male reproductive toxicity, and carcinogenicity.^11^

Microplastics are defined as plastic particles that are <5000 μm in size and can be further classified as primary or secondary depending on their source.^12^ Primary microplastics are those that are intentionally produced to serve a specific function, for example, as microbeads used for exfoliation in cosmetic products.^13^ Secondary microplastics, in contrast, are the breakdown products of larger plastic debris and can be generated by physical, chemical, or biological processes. Secondary microplastics are more prevalent in the environment and can include, for example, the microfibers that degrade from car tires, plastic bottles, and clothing.^14^ Like bulk plastic, microplastics can also be a variety of polymers with different physical and chemical properties.^15^

Microplastics are widespread and mobile in the environment, being detected in air, surface water, costal beaches, sediment, and food.^14,16,17^ They have been discovered in remote and pristine locations, including the Antarctic,^18^ deep ocean trenches,^19^ and Arctic sea ice.^20^ Due to their small size, microplastics more easily enter and are distributed in the human body in comparison to larger particles;^21^ microplastics have been measured in human placenta,^22^ breastmilk,^23^ and liver.^24^ It has been estimated that humans consume a “credit card worth” of microplastics every week.^25,26^ Due to ubiquitous exposure^23^ and bioaccumulative characteristics of microplastics,^17^ the extent of human health impacts due to microplastic exposure is of great concern.

Research on microplastics and their health effects on humans is still in its infancy. A growing body of evidence exists, however, indicating the adverse health effects of microplastic exposure on living organisms.^16^ For example, microplastics increase the susceptibility of fish and seabirds to infections.^27,28^ Microplastics have also been shown to accumulate in organs and lead to biological changes, including oxidative stress and inflammation in human cell lines,^29,30^ and exposure to microplastics has been linked to poor cardiovascular and respiratory outcomes, metabolic disorders, gastrointestinal effects, reproductive effects, and cancer in humans.^29−36^

Evaluations of the human health effects of microplastics have been narrative nonsystematic reviews, not systematic reviews that assess both the quality and strength of the existing evidence, using rigorous, predefined, transparent methods that minimize bias and provide a bottom line summary of the evidence.^29−33,37^ These narrative reviews, therefore, are able to speculate about only the association between microplastic exposure and human health outcomes as they do not follow prespecified, consistently applied, and transparent rules like those utilized by systematic reviews. Systematic reviews are thus needed to provide more confidence in the evaluation of the relationship between microplastic exposure and health effects and to provide a conclusive statement regarding the implications for human toxicity.

Given the growing body of evidence, as well as the urgent need to better characterize the effects of microplastic exposure on human health, we were therefore asked to conduct a rapid systematic review of the evidence to assess the association of microplastic exposures on human health outcomes for policymakers in the State of California (details in Materials and Methods). The primary objectives of this rapid systematic review were to evaluate the human and animal evidence assessing microplastic exposure to any adverse human health outcome,^a^ rate the quality and strength of the human and animal evidence, integrate the human and animal evidence streams and develop a final bottom line statement regarding the health effects of microplastics.

## Materials and Methods

This work builds on a report by the California State Policy Evidence Consortium (CalSPEC), submitted to the State of California in 2023,^16^ which aimed to evaluate the impact of microplastic exposure on human health. CalSPEC seeks to provide rapid, well-researched responses to policymakers in the State of California within a policy cycle, which is less than one year from the time the topic is provided to the report deadline. This prompted our research team to employ rapid systematic review methods rather than conduct a full systematic review.

Rapid reviews represent a type of systematic review that omits certain methodological steps to accelerate the process of performing a full systematic review.^38,39^ This rapid review deviates from a full systematic review in three key ways. (1) After pilot screening, one individual screened all studies, and the other individual screened only excluded studies. (2) After title and abstract screening, a decision was made to narrow the focus to select health outcomes (this is a result of short-circuiting scoping during protocol development; however, we did not look at the study results before prioritizing outcomes). (3) There was one risk of bias assessor and another that quality checked their decisions.

This current publication expands our work on the CalSPEC report to include an evaluation of respiratory outcomes and provides a more detailed description of the methodology we used across the three body systems evaluated.

Our rapid review was guided by the Navigation Guide systematic review method,^40^ which has been implemented to evaluate the health effects of multiple chemical exposures^41−43^ and used by the World Health Organization and International Labor Organization Joint Estimates of the Work-related Burden of Disease and Injury.^44^ We developed and made publicly available a protocol that prespecified our methods for conducting the rapid review on Open Science Framework (OSF).^45^ Due to the condensed time line set by CalSPEC, the review needed to be conducted within a year. Therefore, we prioritized specific health outcomes for inclusion in the review. Deviations from the original protocol (published on OSF October 17, 2022 OSF | The Human Health Effects of Microplastics) are summarized in the updated protocol (published on OSF January 12, 2023) and below in Differences between the Protocol and Systematic Review.

### Study Question

The objective, identified by the CalSPEC team with guidance from the California State Legislature, was to answer the initial research question, “What are the human health effects of microplastics exposure?” The “participants”, “exposure”, “comparator”, and “outcomes” (PECO) statements are outlined below.

#### PECO Statement for Human and Animal Evidence

##### Population

Humans and animals of any age and any health status.

##### Exposure

Any exposure to microplastics, based on our predefined definition of microplastics informed by the State of California,^12^ that occurred prior to or concurrent with diagnosis, exacerbation, or other measure of any health outcome. Exposures can be from any route (air, water, or food), any duration, and any exposure pathway (inhalation, ingestion, or direct contact) and can be measured on the basis of biosamples or from exposure estimates.

We defined microplastics as solid (“solid” means a substance or mixture that does not meet the definitions of a liquid or a gas) and polymeric materials [polymeric material means either (i) a particle of any composition with a continuous polymer surface coating of any thickness or (ii) a particle of any composition with a polymer content] to which chemical additives or other substances have been added, which are particles that are <5000 μm in one dimension. This definition is based on The State of California Water Board definition of microplastics in water^12^ with modification to include all microplastics without a percent content of polymers and lower dimension boundary requirement due to difficulty in measuring and potential exclusion of microplastics that come from surface coatings or tire wear (both of which will be included in our definition of MPs).^15^

##### Comparator

Humans and animals exposed to lower levels of microplastics than the most exposed subjects or treatment groups.

##### Outcome

Any adverse health outcome was assessed. Adverse health outcomes were based on the definition from the U.S. Environmental Protection Agency (“A biochemical change, functional impairment, or pathological lesion that affects the performance of the whole organism, or reduces an organism’s ability to respond to an additional environmental challenge”^46^) and California law hazard trait regulation (Title 22, Cal Code of Regs, Div 4.5, Chapter 54; alternatively 22 CCR 69401 et seq) [“(a) “Adverse effect” for toxicological hazard traits and end points means a biochemical change, functional impairment, or pathologic lesion that negatively affects the performance of the whole organism, or reduces an organism’s ability to respond to an additional environmental challenge. An “adverse effect” for environmental hazard traits and end points means a change that negatively affects an ecosystem, community, assemblage, population, species, or individual level of biological organization.”^47^] Adverse health outcomes included systemic apical end points (e.g., observable end points such as cancer, birth defects, and organ level effects)^48^ and biological responses (e.g., influences DNA/epigenome, oxidative stress, hormone responses, inflammation, immunosuppression, and receptor mediation).

Given the time line, we prioritized digestive, reproductive and respiratory outcomes (see Table 1 for rationales). We did not look at the study results before we made the decision to prioritize these outcomes.

**Table 1:** Rationale for Selecting Specific Health Outcomes

health outcome	rationales
digestive system	(1) food and water are major routes of exposure to microplastics
	(2) the digestive system is a first point of entry for potential toxicity
	(3) there are a range of outcomes associated with this system, including inflammatory disease and cancer
reproductive system	(1) the reproductive system may be particularly sensitive to environmental insults
	(2) this system is of policy interest to regulatory agencies, including the California Environmental Protection Agency
respiratory system	(1) accounts for direct inhalation exposures
	(2) the respiratory system is a first point of entry for potential toxicity
	(3) microplastics are ubiquitous in the air

### Study Search Strategy

We performed a comprehensive search in partnership with a University of California, Davis, medical librarian. The search was first run on July 12, 2022, in PubMed, Embase, ProQuest, and Web of Science and re-run on April 10–15, 2024, and was not restricted by year. The search strategies used in these databases are available in the protocol.^45^ Following the search, de-duplication of references was first conducted in EndNote^49^ and then in Excel before the references were uploaded to DistillerSR for screening, data extraction, and risk of bias evaluation.^50^

### Study Selection

Title/abstract (T/A) and full text screening was informed by our PECO statement and specific inclusion/exclusion criteria. Four screeners (C.B.C., G.B., A.B., and N.C.) reviewed references at T/A and then again at full text using DistillerSR.^50^ Following Cochrane’s Rapid Review guidance,^38^ C.B.C., G.B., A.B., and N.C. independently screened 30 of the same references at T/A to pilot the form and then continued to dual screen 20% of the references. Thereafter, C.B.C. and N.C. reviewed all of the remaining references at T/A for inclusion and G.B. and A.B. reviewed only references that C.B.C. and N.C. had tagged for exclusion.

A similar process was applied for the screening at full text using Cochrane’s Rapid Review guidance.^38^ C.B.C., G.B., A.B., and N.C. pilot screened the same five references at full text to test the form and calibrate their screening. After this, C.B.C. and N.C. screened all references at full text for inclusion and G.B. and A.B. verified only references that C.B.C. and N.C. had tagged for exclusion.

For both T/A and full text screening, any disagreements in terms of inclusion or exclusion of references were first reviewed and discussed between reviwers. If the reviewers could not reach a consensus, N.C. and T.J.W. served as arbitrators to make the final decision.

### Eligibility Criteria

As this was a rapid systematic review, less restrictive eligibility criteria, which can be found in Differences between the Protocol and Systematic Review, was applied during the T/A screening.

#### Final Inclusion Criteria

Ultimately, studies were included if they adhered to the PECO statement and met the following criteria.

- published in English or with an English version online
- primary human observational studies, including, cohort, case-control, cross-sectional, or other relevant designs
- experimental animal studies such as mammalian rodent studies (rats and mice)
- reported exposure to microplastics, as defined by the PECO statement
- comparator group with no or lower levels of microplastic exposure
- measured any outcome of the digestive system (excluding gut microbiota outcomes), reproductive system, or respiratory system
- outcomes reported quantitatively (*p* values and figures considered sufficient)
- mammalian rodents (rats and mice) exposed by oral route via food and/or water (digestive and reproductive studies) or intratracheal or intranasal routes (respiratory studies)
- mammalian rodent (rats and mice) studies evaluated repeated exposures to microplastics
- mammalian rodent (rats and mice) exposed to multiple concentrations of microplastics (i.e., more than one exposed group)

#### Final Exclusion Criteria

Studies were excluded if one or more of the following criteria were not met.

- does not contain original data (e.g., commentary, editorial, review, etc.)
- in a language other than English
- does not involve human or mammalian rodent (rats and mice) animals (i.e., cell line only, plants, non-rodent mammal studies, or rodents other than rats and mice)
- does not report exposure to microplastics, as defined by the PECO statement
- no comparator group
- mammalian rodents (rats and mice) exposed to microplastics via gavage, dermal exposures, intraperitoneal injection, caudal vein injection, or intragastric administration
- mammalian rodent (rats and mice) studies that evaluated only one exposure group versus a control
- case report of a single participant
- other reasons (explanation required)

### Data Extraction

We utilized DistillerSR for data extraction of study characteristics, including exposure and outcome information, and numerical results of the study (e.g., *p* values and dose response as reported in the studies).^50^ Our data extraction forms are available in the protocol (appendices C, D, and E).^45^ C.B.C., G.B., and N.C. all participated in data extraction for reproductive and digestive outcomes. C.B.C., G.B., N.C., A.B., and K.E.P. all extracted information about respiratory outcomes. A single reviewer extracted relevant data from included studies, and a second reviewer checked the extracted data for correctness and completeness.^38^ Any discrepancies were discussed, and N.C. and T.J.W. served as arbitrators in the event that a consensus could not be reached.

We planned on extracting the mean and standard error from each study; however, as described in Analysis, the quantitative data were very limited due to poor reporting in studies, and *p* values were often the only data available to extract. Additionally, the figures were extracted to provide Supporting Information to allow visual assessment of the dose response (control group compared to the largest dose of microplastics).

### Types of Outcome Measures

We organized outcomes by apical outcomes and biological changes. For the organization of biological changes, we were guided by the concept of “key characteristics”. Key characteristics are biomarkers or mechanistic effects that comprise properties of known human carcinogens or reproductive toxicants [these charcteristics of carcinogens include (1) electrophilicity, (2) genotoxicity, (3) altering DNA repair or causeing genomic instability, (4) inducing epigenetic alterations, (5) inducing oxidative stress, (6) inducing chronic inflammation, (7) being immunosuppressive, (8) modulating receptor-mediated effects, (9) causing immortalization, and (10) altering cell proliferation, cell death, or nutrient supply].^51−57^ For the digestive and respiratory outcomes, we utilized the key characteristics of carcinogens.^53^ For reproductive health outcomes, we utilized the key characteristics of reproductive toxicity.^51,56^

We considered every eligible outcome in human studies. We prioritized the apical and biological outcomes listed in Table 2 for animal studies on the basis of what we considered to be the most relevant for each system. We did not look at the study results before prioritizing outcomes. See Table 2 and Supporting Information File 4 (“Study results tables”) for all study results by system.

**Table 2:** Eligible Outcomes Included in Our Rapid Review of the Effects of Microplastic Exposure on Human Digestive, Reproductive, and Respiratory Health

eligible outcomes	
digestive	included for analysis
	apical end points (gross or microanatomic colon and intestine effects)
	key characteristics of carcinogens (chronic inflammation, oxidative stress, immunosuppressive effects, cell proliferation, and receptor-mediated effects)
	excluded from analysis
	key characteristics of carcinogens (epigenetic alterations, effects on DNA repair, or genomic instability)
reproductive	included for analysis
	apical end points (sperm-related outcomes, follicle/ovarian reserve capacity, oocyte meiotic progression, blatstocyst development, and angiogenital distance)
	apical end points (birth outcomes such as the weight of fetus and placenta and litter size)
	other (age at puberty)
	key characteristics of reproductive toxicants (alterations in reproductive hormones)
	excluded from analysis
	apical end points (body weight and testicular damage)
	key characteristics of carcinogens (oxidative stress, epigenetic alterations, genotoxicity, inflammation, alterations in immune function; male, changes in germ or somatic cells; female, altered survival, proliferation, cell death, or metabolic pathways)
respiratory	included for analysis
	apical end points (total cell count, lung injury, and pulmonary function)
	key characteristics of carcinogens (chronic inflammation and oxidative stress)
	excluded from analysis
	apical end points (protein levels in lung)
	key characteristics of carcinogens (immunosuppressive, induces epigenetic alterations, and alters cell proliferation, cell death, or nutrient supply)

### Rate the Quality and Strength of the Evidence

#### Assessing the Risk of Bias

We used the Navigation Guide risk of bias tool to evaluate human and animal studies.^41,43,58,59^

In human studies, this contains nine domains: “study group representation”, “knowledge of group assignments”, “exposure assessment methods”, “outcome assessment methods”, “confounding”, “incomplete outcome data”, “selective outcome reporting”, “conflict of interest”, and “other”. In animal studies, this tool contains seven domains that are evaluated for each study and/or outcome: “sequence generation”, “allocation concealment”, “blinding of personnel and outcome assessors”, “incomplete outcome data”, “selective outcome reporting”, “conflict of interest”, and “other potential threats to validity”. We developed customized instructions for evaluating the validity of how the outcome assessment was conducted for the domain “other potential threats to validity”.

Possible ratings for each domain were “low”, “probably low”, “probably high”, or “high” risk of bias, Prior to conducting risk of bias assessments, all individuals (C.B.C., K.E.P., G.B., N.C.) reviewed training materials from a systematic review expert (J.L., listed in the acknowledgements) and our subject-matter experts (G.B. and S.A.G.) discussed important criteria for considering the “blinding of personnel and outcome assessors”, “incomplete outcome data”, and “other threats to validity” (how the assessment of outcomes was conducted). For more details on the process of evaluating the risk of bias for each study, see the protocol in ref (45).

We used a single reviewer to evaluate the risk of bias (N.C. for digestive, respiratory, and reproductive outcomes and K.E.P. for respiratory outcomes) for each study by outcome, while a second reviewer (G.B. for digestive and reproductive outcomes and S.A.G. and T.J.W. for respiratory outcomes) verified the judgements.^38^ Any disagreements were first discussed between reviewers, with T.J.W. serving as an arbitrator for any instances in which a consensus could not be reached.

The risk of bias was evaluated on an outcome level, meaning that different health outcomes in a study could receive different ratings within a single domain. We visually depicted and reported the ratings and rationales for each risk of bias domain across each study.

### Analysis

We analyzed the result representing the effect of the highest level of microplastic exposure compared with the lowest level of microplastic exposure (i.e., highest concentration of microplastics compared to the control group). We used the information extracted on study characteristics to assess the comparability across studies and determine whether biological heterogeneity was a concern. We then combined end points that were biologically similar across each system to synthesize results; e.g., for digestive outcomes and chronic inflammation, we combined study results measuring TNF-α, IL-2, IL-5, IL-6, IL-9, IL-10, IP-10, IL-1α, Ifng, Il1b, G-CSF, RANTES, iNOS expression, COX-2 expression, NF-kB, and mRNA expression.

We planned on extracting the mean and standard error from each study and utilizing a two-step analysis to conduct meta-analysis if the data were sufficiently homogeneous. However, these data were not available in almost every study or too heterogeneous to combine. For example, papers reported only point estimates, estimates were reported on different scales or used different association metrics, or the scales on the figures were not fully reported preventing us from converting the results across studies into a single scale. As this is a rapid review, we did not contact study authors for missing data.

We therefore used established methods of Cochrane for statistical synthesis when meta-analysis of effect estimates was not possible.^60^ As we were unable to combine *p* values as there were only *p* values for studies with statistical significance, we instead estimated the proportion of effects favoring the intervention along with a confidence interval (e.g., using the Wilson interval methods).^61^

Additionally, we assessed (1) the statistical significance (*p* value representing statistically significant differences between control and the most exposed group at follow-up) and (2) whether a dose–response relationship was identified for each outcome included.

For each synthesis that has concluded microplastics harm human health, we visually display the results included in the synthesis by adapting a Harvest plot to include the direction of effect, *p* value, and significance (e.g., <0.001, <0.01, ≤0.05, or >0.05), whether a dose response was identified, and the sample size of the study. However, we were unable to conduct subgroup analysis or meta-regression to explore heterogeneity in the study results.

We classified outcomes as showing harm from microplastic exposure if there was a change in effect in between the most exposed group and the non-exposed/least exposed group in the direction indicating harm (between group analysis).

We acknowledge that our approach has limitations; however, we have avoided placing increased weight on statistical significance that does not address biological significance or the magnitude of the effect observed.^62^ For outcomes for which we did not conclude that microplastics harm human health, we narratively present the results for each outcome.

### Sensitivity Analysis

We conducted a sensitivity analysis to test the robustness of our results when including only one type of microplastic and only one study result per outcome to the synthesis, as per Cochrane guidance.^63^ For studies that had multiple (more than two) eligible study results for an outcome (e.g., for the outcome “induction of chronic inflammation”, a study measured and reported both IL-6 and TNFα), we randomly selected one result. We used two proportion *Z* tests to measure statistically significant differences between proportions of effects [i.e., one type of microplastic (e.g., polystyrene only) vs another type of microplastic (e.g., polyethylene only) and/or when only one study result per outcome was included in the synthesis versus our primary analysis of including all study results for each study per outcome in the synthesis] at the 0.05 level (two-tailed).

#### Quality of the Evidence across Studies

**Figure 1:**
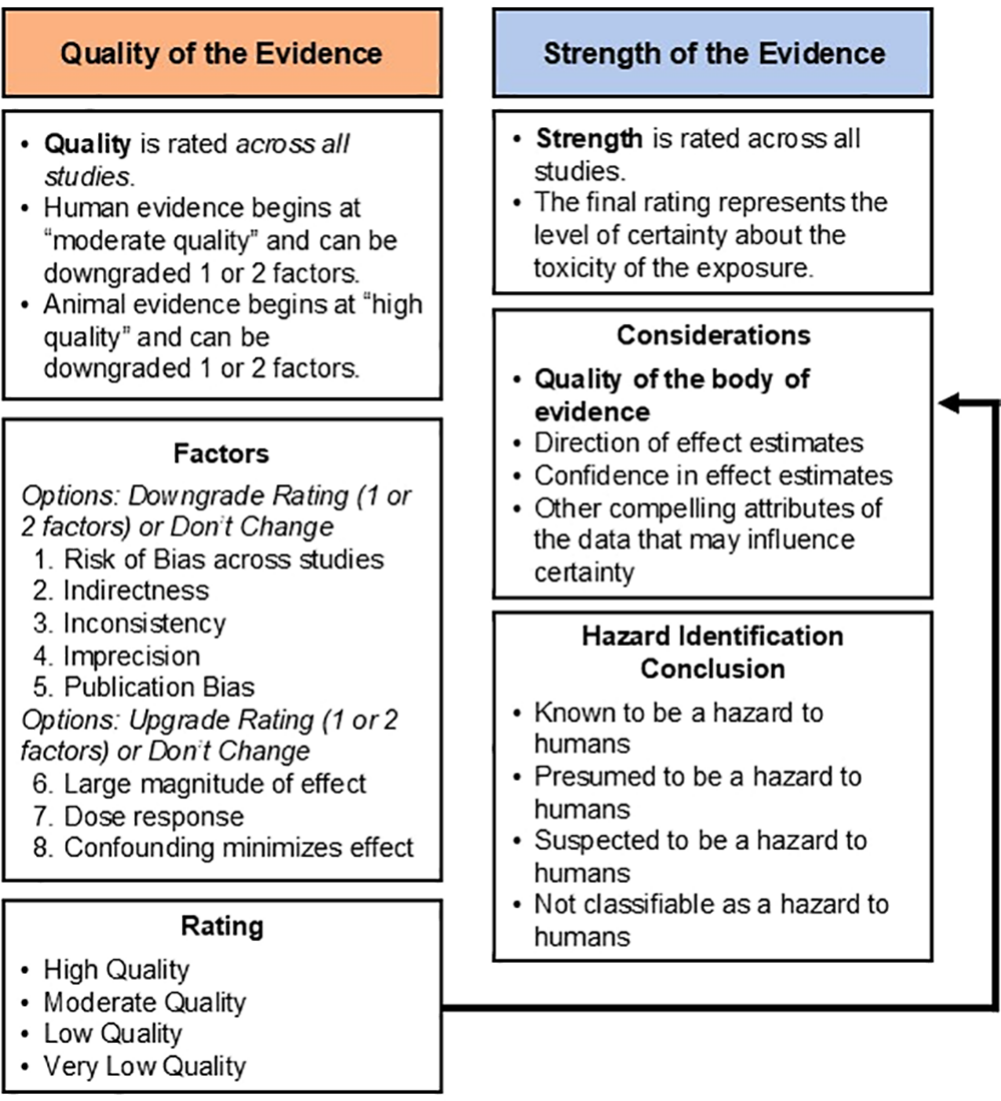
Evaluating the quality and strength of the body of evidence using Navigation Guide.

We assessed the overall quality of the body of evidence for each health effect. Evidence from human studies was initially rated “moderate”, and for experimental animal studies, the quality of each body of evidence was initially rated “high” on the basis of a previously described rationale (see Figure 1).^43,64^ As animals can be randomized before being exposed to toxic hazards like microplastics, this eliminates selection bias and the potential influence of confounding, and they are therefore started at a higher level of certainty. The rating of the quality of each body of evidence was then adjusted on the basis of eight factors and could ultimately be rated as “high”, “moderate”, “low”, or “very low”. The quality of each body of evidence could be downgraded by five factors: “risk of bias across studies”, “indirectness”, “inconsistency”, “imprecision”, and “publication bias”. The quality of each body of evidence could be upgraded by three factors: “large magnitude of effect”, “dose response”, and “accounting for confounding that might minimize the effect” (Figure 1). The possible ratings for each downgrade or upgrade factor were 0 (no change from the initial quality rating), −1 (one-level downgrade), −2 (two-level downgrade), +1 (one-level upgrade), or +2 (two-level upgrade). Review authors (C.B.C., N.C., G.B., K.E.P., T.J.W., and S.A.G.) independently evaluated the quality of the evidence across studies, and then ratings were compared as a group. We recorded (and present) the consensus and rationale for each factor and each final decision.

#### Strength of the Evidence

**Figure 2:**
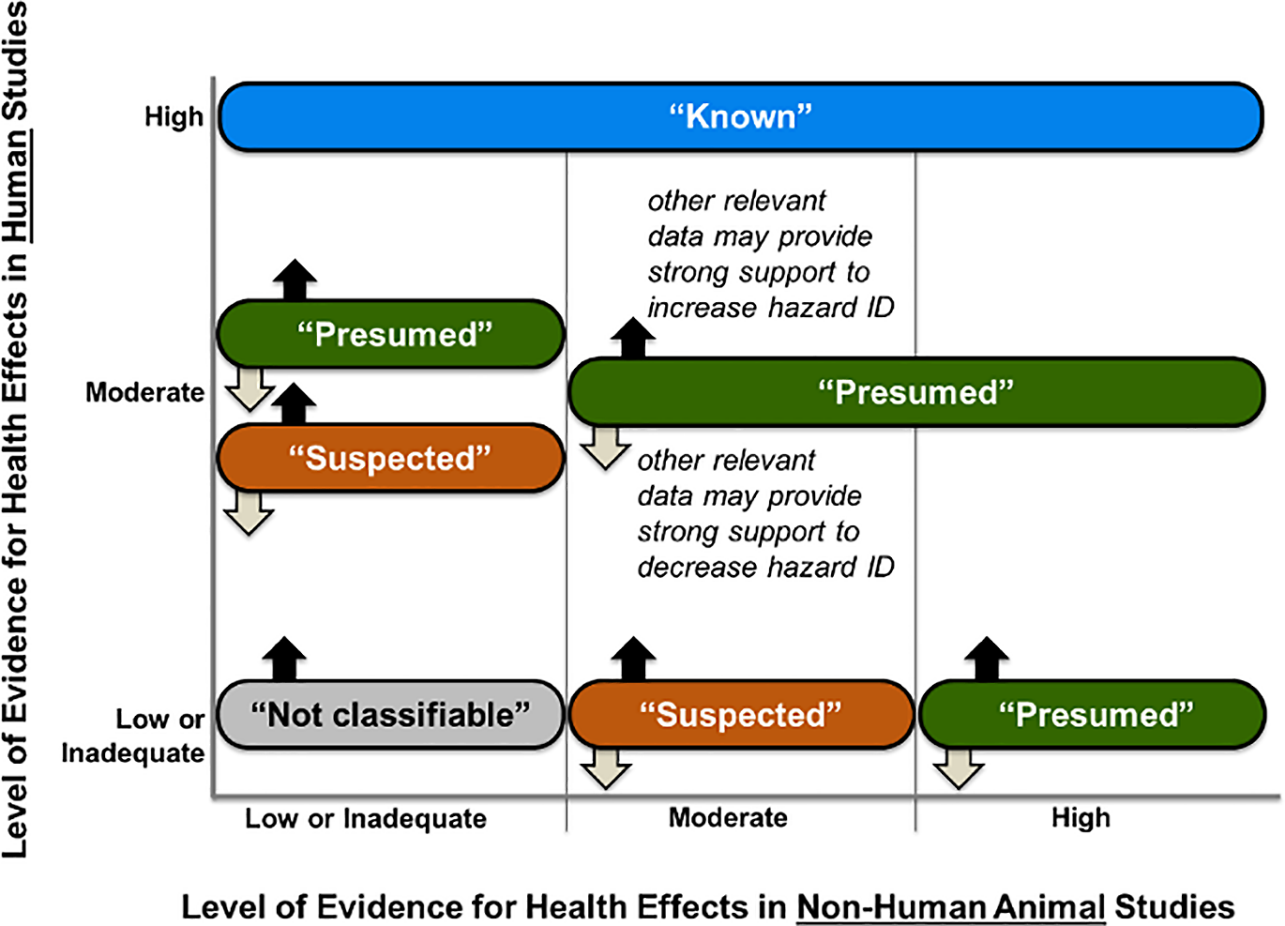
Hazard identification conclusion statements informed by the NTP OHAT approach.

We rated the overall strength of the body of evidence (Figure 1) on the basis of four considerations: (1) “quality of the body of evidence”, (2) “direction of effect estimates”, (3) “confidence in the effect” (considering factors such as the number and size of studies), and (4) “any other compelling attributes of the data that may influence certainty”. This informed the final hazard conclusion statements, which were guided by the National Toxicology Program Office of Health Assessment and Translation (NTP OHAT) approach.^64^ There were four possible conclusions regarding the risk of microplastic exposure to humans outlined in Figure 2: (1) “known” to be a hazard to humans, (2) “presumed” to be a hazard to humans, (3) “suspected” to be a hazard to humans, and (4) “not classifiable” as a hazard to humans.

## Differences between the Protocol and Systematic Review

### Eligibility Criteria

After running the first search in July 2022, we amended the eligibility criteria after screening studies at T/A for the following reasons.

1. We identified no studies that had evaluated the impact of microplastic exposure on human health using human subjects.
2. Given the lack of epidemiological evidence, we prioritized exposure pathways that most directly mimic human experiences in animal studies.
3. We focused our review on mammalian rodent studies, specifically rats and mice, which have been robustly used by regulatory agencies to identify potential human harm.^65−67^
4. The time line for this rapid review was driven by a legislative cycle, meaning that we had to be judicious about the number of studies and health outcomes we had the capacity to evaluate as a team.

### Inclusion Criteria (original, applied at T/A screening)

Studies were included if they adhered to the PECO statement and met the following criteria.

- published in English or with an English version online
- primary human observational studies, including cohort, case-control, cross-sectional, or other relevant designs
- experimental animal studies
- reported exposure to micoplastics, as defined by the PECO statement
- comparator group with no or lower levels of microplastics
- measured any health outcome relevant to human health
- outcomes reported quantitatively
- experimental animal studies evaluated repeated exposures to MPs

### Exclusion Criteria (original, applied at T/A screening)

Studies were excluded if one or more of the following criteria were not met.

- does not contain original data (e.g., commentary, editorial, review, etc.)
- does not involve human subjects or animals (i.e., cell line only, plants, and rodents other than rats and mice)
- no comparator group
- case report of a single participant
- other reasons (explanation required)

### Outcomes

We had planned to prioritize analyzing only digestive and reproductive outcomes, while narratively summarizing respiratory (which we originally described as pulmonary) studies. After publishing the report, we fully analyzed the respiratory outcomes.

### Analysis

We planned on extracting the mean and standard error from each study and utilizing a two-step analysis to conduct meta-analysis if data were sufficiently homogeneous. However, these data were not available in almost every study or too heterogeneous to combine. For example, papers reported only point estimates, estimates were reported on different scales and used different association metrics, or the scales on the figures were not fully reported, preventing us from converting the results across studies into a single scale. As this is a rapid review, we did not contact study authors for missing data.

We therefore used established methods by Cochrane for statistical synthesis when meta-analysis of effect estimates was not possible.^60^ As we were unable to combine *p* values as there were *p* values only for studies with statistical significance, we instead estimated the proportion of effects favoring the intervention along with a confidence interval (e.g., using the Wilson interval methods).^61^

### Sensitivity Analysis

We had planned to conduct a sensitivity analysis if a meta-analysis had been performed by examining the effects of excluding studies with particular heterogeneous results as well as performing subgroup analyses based on heterogeneous characteristics identified from the review for comparability across studies. However, as we were unable to conduct a meta-analysis, we instead conducted a sensitivity analysis to test the robustness of our results when including only one type of microplastic and only one outcome per study contributing to the synthesis as per Cochrane guidance.^63^

## Results

The initial search identified 1815 unique studies for screening from which 17 animal studies met our inclusion criteria for data extraction (see Supporting Information File 1, “Study Flow Diagram”). The second search identified an additional 1042 studies, with 14 included (three human and 11 animal). See Supporting Information File 2 (“List of excluded studies and reasons for exclusion at full text review”).

### Characteristics of Included Studies

Three human cross-sectional observational studies examined reproductive (*n* = 2) and respiratory (*n* = 1) outcomes, and 28 experimental animal studies examined reproductive (*n* = 11), respiratory (*n* = 7), and digestive (*n* = 10) outcomes.

#### Human

Human studies were published from 2022 to 2024. Total study populations ranged from 40 to 80. Human studies were conducted in Turkey (*n* = 1), Iran (*n* = 1), and China (*n* = 1). Microplastics were measured in maternal amniotic fluid (*n* = 1), placenta (*n* = 1), and nasal lavage fluid (*n* = 1). Microplastics were characterized by polymer type in two (66%) of the studies. Polystyrene, polyethylene, polyethyleneterephthalate, polypropylene, chlorinated polyethylene, polyamide, and others were detected.

#### Animal

Animal studies were published from 2018 to 2024 and were mostly conducted in China [*n* = 22(79%)]. Most of the animal studies [*n* = 22(79%)] were conducted in mice with 15–180 rodents per study. The total number of rodents per exposure group ranged from five to 45. The number of exposure groups ranged from two to four per study. See Supporting Information File 3 (“List of included studies and study characteristics”).

In animals, microplastics were administered through ingestion in water [*n* = 16 (57%)], in food [*n* = 5 (18%)], or via inhalation [*n* = 7(25%)]. Microplastics in inhalation studies were suspended in air, water, or saline with various methods of delivery, including, for example, intranasal inhalation and intratracheal instillation. Exposures lasted from 14 days to 32 weeks. Nearly all exposures were in adult aged animals, with mice in two reproductive studies being exposed during early life development (i.e., during gestation or during gestation and postnatal development). There was little variability in study design and the types, sizes, and shapes of the microplastics across the 28 animal studies. The type of microplastic used was overwhelmingly polystyrene [*n* = 22(79%)]. The size of microplastics administered was between 0.1 and 467.85 μm.

Animal studies covering digestive and respiratory outcomes were conducted in China, France, and the Republic of Korea, and reproductive outcome studies were conducted in China, Pakistan, and Canada. Some publications were produced by the same lab group, raising the possibility that errors in the method or approach might be propagated across multiple studies. Two lab groups produced two digestive papers each,^68−71^ while another published three reproductive papers.^72−74^ See Tables 3–6 for included human and animal studies with further details in Supporting Information Files 3 and 4.

**Table 3:** Human Studies Evaluating Reproductive and Respiratory Outcomes Included in Our Rapid Review of the Effects of Microplastic Exposure

ref	study population	microplastic size and type	outcomes
(35)	43 pregnant women	PET (polyethylene terephthalate)	reproductive: growth outcomes (birth weight, length, and head circumference)
		polypropylene (PP)	
		PE (polyethylene)	
		PS (polystyrene)	
		(mean size of 9.86 μm)	
(36)	40 pregnant women	PE (polyethylene)	reproductive: growth outcomes (birth weight)
		CPE (chlorinated polyethylene)	
		PA (polyamide)	gestational age
		PU (polyurethane)	
		PP (polypropylene)	
		EVA (ethylene vinyl acetate copolymer)	
		SBS (styrene–butadiene–styrene)	
		PET (polyethylene terephthalate)	
		PVC (polyvinyl chloride)	
		(20.34–467.85 μm)	
(91)	80 people (50 patients with chronic rhinosinusitis without nasal polyp and 30 healthy volunteers)	N/A	respiratory: chronic rhinosinusitis

**Table 4:** Animal Studies Evaluating Digestive Outcomes Included in Our Rapid Review of the Effects of Microplastic Exposure

ref	study population	microplastic size and type	exposure route/frequency/duration/concentration	outcomes
(70)	24 mice	5 μm polystyrene	water ingestion/continuous/6 weeks/100 or 1000 μg/L	apical: gross or microanatomic colon effects
(71)	40 mice	0.5 and 50 μm polystyrene	water ingestion/continuous/5 weeks/100 or 1000 μg/L	apical: gross or microanatomic colon effects
(78)	80 mice	10–150 μm polyethylene	food ingestion/daily/5 weeks/2, 20, or 200 μg	key characteristic: chronic inflammation
(68)	40 mice	500 nm polystyrene	water ingestion/daily/2 weeks/10, 50, or 100 μg/g	key characteristics: chronic inflammation and oxidative stress
(69)	24 mice	5 μm polystyrene	water ingestion/daily/2 weeks/10, 50, or 100 μg/L	apical: gross or microanatomic colon effects
				key characteristics: alterations in cell proliferation, cell death, or nutrient supply and receptor-mediated effects
(75)	39 mice	36 and 116 μm (median sizes) polyethylene	food ingestion/continuous/6 weeks/100 or 200 μg	apical: gross or microanatomic colon and small intestine effects
				key characteristics: chronic inflammation and immunosuppression
(76)	49 mice	5 μm polystyrene	water ingestion/daily/90 days/100 or 1000 μg/L	apical: gross or microanatomic colon effects
				key characteristics: changes in cell proliferation, cell death, or nutrient supply; chronic inflammation; and oxidative stress
(79)	180 female mice	∼50 nm polystyrene	water ingestion/daily/32 weeks/0.1, 1, or 10 mg/L	key characteristics: oxidative stress, immunosuppression, and chronic inflammation
(77)	60 male mice	40–60 and 40–100 μm polystyrene	food ingestion/continuous/21 weeks/50 or 500 mg/kg of food	apical: gross or microanatomic colon effects
(80)	42 female mice	30 and 200 μm polyethylene	food ingestion/daily/35 days/2, 20, or 200 μg	key characteristics: oxidative stress

**Table 5:** Animal Studies Evaluating Reproductive Outcomes Included in Our Rapid Review of the Effects of Microplastic Exposure

ref	study population	microplastic size and type	exposure route/frequency/duration/concentration	outcomes^a^
(72)	32 female rats	0.5 μm polystyrene	water ingestion/continuous/90 days/0.015, 0.15, or 1.5 mg	apical: female reproductive outcomes (follicles/ovarian reserve capacity)
				key characteristics: alterations in hormone receptor signaling and/or reproductive hormone production, secretion, or metabolism
(85)	40 male mice	5 μm polystyrene	water ingestion/daily/35 days/100 μg/L, 1000 μg/L, or 10 mg/L	apical: male reproductive outcomes (sperm and sperm-related outcomes)
(73)	32 female rats	0.5 μm polystyrene	water ingestion/daily/90 days/0.015, 0.15, or 1.5 μg/g	apical: female reproductive outcomes (follicles/ovarian reserve capacity)
				key characteristic: alterations in hormone receptor signaling and/or reproductive hormone production, secretion, or metabolism
(74)	32 male rats	0.5 μm polystyrene	water ingestion/daily/90 days/0.015, 0.15, or 1.5 mg	apical: male reproductive outcomes (sperm and sperm-related outcomes)
(86)	32 female mice	100 nm polystyrene	water ingestion/continuous/21 days/0.1, 1, or 10 mg/L	apical: male reproductive outcomes (sperm and sperm-related outcomes)
				other: litter size
(84)	105 male mice	0.5, 4, or 10 μm polystyrene	water ingestion/continuous/180 days/100 or 1000 μg/L	apical: male reproductive outcomes (sperm and sperm-related outcomes & germinal cell thickness)
				key characteristic: alterations in production and levels of reproductive hormones or hormone receptor levels and/or functions
(83)	30 female rats	876 nm polystyrene	food ingestion/daily/45 days/2.5, 5, or 10 mg/kg/day	key characteristic: alterations in production and levels of reproductive hormones and/or hormone receptor levels and/or functions
(87)	40 mice	0.5 μm polystyrene	water ingestion/daily/35 and 70 days/0.5, 5, or 50 mg/L	apical: anogenital index and distance
				apical: male reproductive outcomes (sperm and sperm-related outcomes)
				other: age at puberty
				key characteristic: alterations in production and levels of reproductive hormones or alters hormone receptor levels and/or functions
(82)	40 mice	10–150 μm polyethylene	water ingestion/daily/30 days/0.4, 4, or 40 mg/kg/day	apical: oocyte meiotic progression and blatstocyst development
				other: litter size
				key characteristic: alterations in production and levels of reproductive hormones or alters hormone receptor levels and/or functions
(88)	40 female mice	5 μm polystyrene	water ingestion/continuous/15.5 days/102, 104, or 106 ng/L	apical: weight of fetus and placenta
(89)	15 male mice	1 μm polystyrene	water ingestion/daily/1 mg/kg (low dose) or 5 mg/kg	apical: male reproductive outcomes (testicular aging)

a The outcomes column does not contain all of the outcomes in the study, only the outcomes prioritized for data extraction.

**Table 6:** Animal Studies Evaluating Respiratory Outcomes Included in Our Rapid Review of the Effects of Microplastic Exposure

ref	study population	microplastic size and type	exposure route/frequency/duration/concentration	outcomes
(92)	40 rats	0.10 μm polystyrene	air inhalation/daily/6 h per day, 5 days a week for 2 weeks/0.75 × 10^5^, 1.50 × 10^5^, or 3.00 × 10^5^ particles/cm^3^ ± 20%	apical: pulmonary function
				apical: total cell count
				key characteristic: induces chronic inflammation
(93)	40 mice	<1 μm tire wear microplastic particles	saline inhalation/daily/28 days/0.12, 0.5, or 1 μg/g	apical: pulmonary function
				apical: total cell count
				apical: lung injury
(98)	20 rats	100 nm, 500 nm, 1 μm, and 2.5 μm polystyrene	saline inhalation/unclear/14 days/0.5, 1, or 2 mg/200 μL	key characteristic: induces chronic inflammation
(97)	36 mice	5 μm polystyrene	water inhalation/three times a week/3 weeks/1.25 or 6.25 μg/g	key characteristic: induces chronic inflammation
				key characteristic: induces oxidative stress
(96)	30 male mice	10 μm and 20 nm polystyrene	intranasal inhalation/days 1, 3, 5, 7, 9, 11, 13, and 15/5 or 10 mg/kg	apical: lung injury
(95)	24 male mice	40 nm polystyrene	inhalation tower/daily/1 week, one month, and three months/16, 40, or 100 μg	apical: cell count
				apical: pulmonary function
				apical: lung injury
				key characteristic: induces chronic inflammation
				key characteristic: induces oxidative stress
(94)	24 male mice	0.66 ± 0.27 μm polypropylene	intratracheal instillation/five times per week/4 weeks/1, 2.5, or 5 mg/kg	apical: lung injury
				apical: cell count
				key characteristic: induces chronic inflammation
				key characteristic: induces oxidative stress

### Risk of Bias

See Supporting Information File 5 (“Risk of bias heat map for summaries of risk of bias judgments”) for the studies included in our systematic review of microplastic exposure. Risk of bias heat maps are provided for each outcome (digestive, reproductive, and respiratory) for each evidence stream (human and animal). Risk of bias designations for individual studies are assigned according to criteria provided in the protocol,^45^ and the justification for each study is provided in Supporting Information File 6 (“Risk of bias ratings and justification”).

### Digestive Results

There were no human studies examining this outcome.

We evaluated six outcomes across 10 studies relating to the small or large intestines of the digestive tract, focusing on apical end points (in this case, gross or microanatomic colonic and small intestinal effects) and biological outcomes grouped into the following key characteristics of carcinogens: oxidative stress, chronic inflammation, immunosuppression, receptor-mediated effects (hormones), and cell proliferation (e.g., goblet cell count).

Similar measurements were conducted between studies; however, not all measurements were the same, and estimates could not be combined in a meta-analysis or visually displayed collectively in a figure because estimates were reported on different scales, used different association metrics, or were not fully reported.

#### Apical Outcomes (colon and small intestine)

Six studies evaluated apical measurements on the digestive tract, including colon length, villus length, and other histopathological measurements of the colon and small intestine^69−71,75−77^ (see Supporting Information File 4, “Study Results”).

For the risk of bias, one study was high or probably high for five domains,^69^ one study was high or probably high for two and three domains (different apical outcomes with different ROB ratings),^76^ three studies were high or probably high for two domains,^70,71,75^ and one study was high or probably high for one domain^77^ (see Supporting Information Files 5, “Risk of bias heat map”, and 6 for justification of ratings).

For microplastic type, five studies tested polystyrene^69−71,76,77^ and one tested polyethylene microplastics.^75^

One study^76^ observed significant alterations to the colon, including changes in the muscular layer width. The same study also found significant colon shortening in the exposed group. Another study^75^ observed significant differences in crypt depth but not the villus length in the proximal and distal small intestines for the most exposed group (which throughout this section will often be termed the “exposed group”). The same study also observed a significant increase in the mucosal surface area in the colon epithelium but found opposite or no significant change in staining with neutral and acid mucins in different parts of the digestive system. The third study^69^ found a significant decrease in multiple histopathological end points. The fourth study^71^ found a significant decrease in the extent of mucus secretion in colon for the exposed group. The fifth found a significant decrease in the thickness of the mucosa layer of the small intestine.^77^ The final study found a significant decrease of the alcian blue-periodic acid Schiff (AB/PAS) solution positive area (area with mucins) in all microplastic exposure groups compared to control (unexposed) but did not exhibit a dose response effect across the groups.^70^

**Figure 3:**
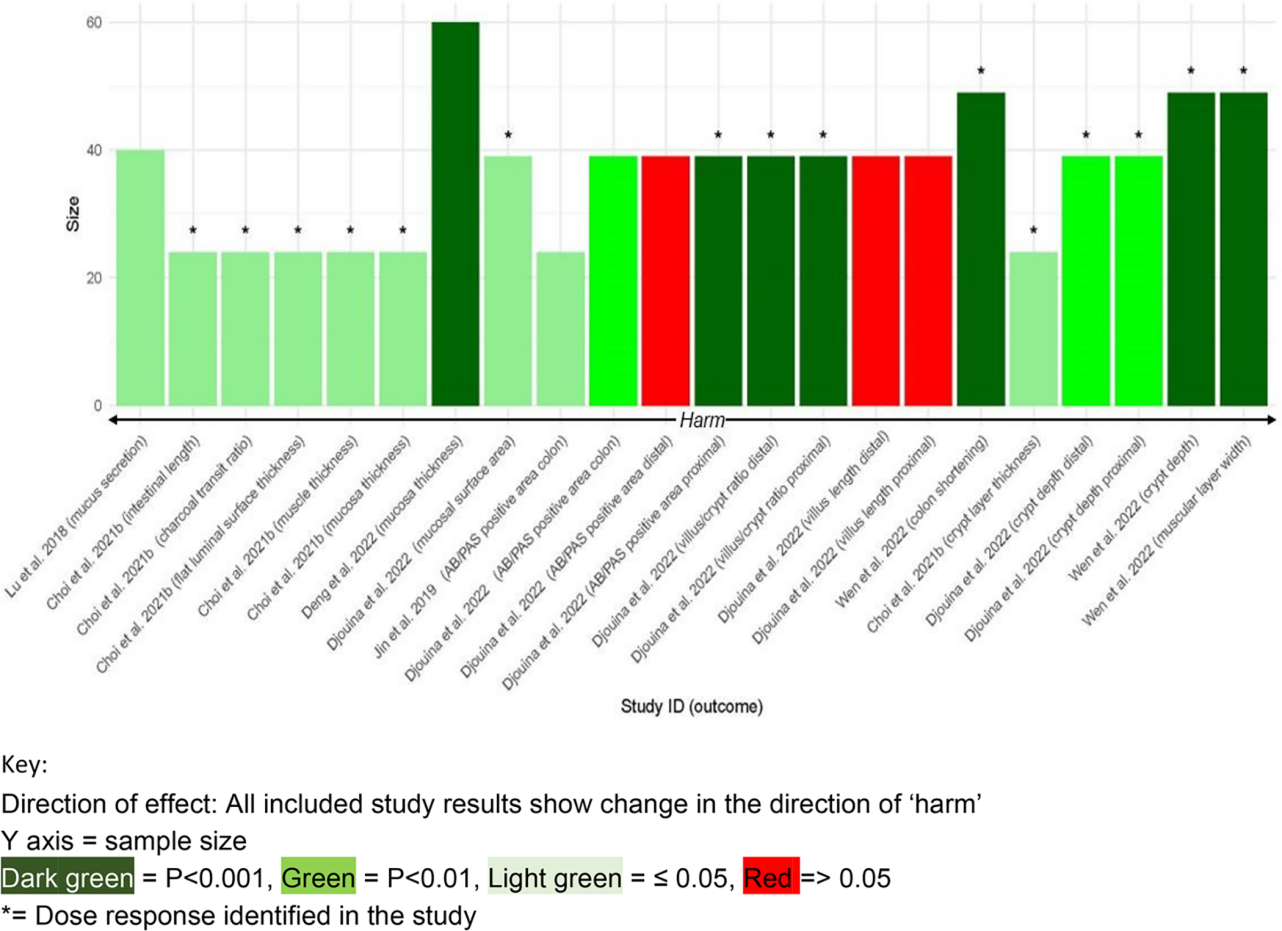
Apical outcomes (colon and small intestine).

The estimate of the proportion of effects showing microplastics are harmful equals 1.00 [95% confidence interval (CI) of 0.85–1.00] [*n* = 22 (positive study results)/22 (total study results)] [see Figure 3 for (1) the direction of the effect, (2) *p* values, (3) the dose response, and (4) the study sample size for included studies in this synthesis].

We conducted a sensitivity analysis and (1) compared the estimate of the proportion of effects showing polystyrene microplastics are harmful = 1.00 (95% CI of 0.76–1.00) (*n* = 12/12) versus polyethylene microplastics = 1.00 (95% CI of 0.72–1.00) (*n* = 10/10) and (2) compared the proportion of effects showing microplastics are harmful when only one result per study was considered = 1.00 (95% CI of 0.61–1.00) (*n* = 6/6) versus our primary analysis of including all study results from each study = 1.00 (95% CI of 0.85–1.00) (*n* = 22/22). See Supporting Information File 7 (“Sensitivity analysis”).

We found no difference in the proportions of effects between polystyrene microplastics and polyethylene microplastics, and we found no difference in the proportions of the effect between our analysis of only one result per study being considered versus our primary analysis of including all study results from each study.

We concluded that exposure to microplastics is “suspected” to adversely impact the colon and small intestine in humans on the basis of (a) the “moderate” quality of the body of evidence [see Supporting Information File 8 (“Evidence ratings for studies”) for a detailed rationale for these ratings], (b) the direction of the effect (i.e., evidence of an increasing adverse health effect with an increasing level of microplastic exposure), and (c) the confidence in the association, considering factors including the number and size of studies.

#### Biological Changes (key characteristics)

##### Alterations of Cell Proliferation, Cell Death, or Nutrient Supply

Two studies assessed cell proliferation and death.^69,76^

For the risk of bias, one study was high or probably high for five domains^69^ and one high or probably high for three domains^76^ (see Supporting Information Files 5 and 6).

For the microplastic type, both studies tested polystyrene microplastics. The first study showed a significant decrease in the number in crypts of Lieberkuhn (intestinal mucosal glands) and goblet cells (cells that secrete mucin) in the exposed group.^69^ The second study also found a significant decrease in the number of goblet cells.^76^ See Supporting Information File 4 (“Study Results”).

The estimate of the proportion of effects showing microplastics are harmful = 1.00 (95% CI of 0.44–1.00) (*n* = 3/3). See Supporting Information File 9 (“Graphical display of results”) and Figure S1. We did not conduct a sensitivity analysis as every result was in the direction of showing harm.

We concluded that exposure to microplastics is “suspected” to adversely impact intestinal cell proliferation and cell death in humans on the basis of (a) the “moderate” quality of the body of evidence (see Supporting Information File 8, “Evidence ratings for studies”), (b) the direction of the effect (i.e., evidence of an increasing adverse health effect with an increasing level of microplastic exposure), and (c) the confidence in the association considering factors including the number and size of studies.

##### Induction of Chronic Inflammation

Five studies evaluated biomarkers (e.g., inflammatory cytokines) related to chronic inflammation.^68,75,76,78,79^

For the risk of bias, one study was rated high or probably high for four domains,^68^ one study was rated high or probably high for three domains,^78^ two studies were rated high or probably high for two domains,^75,76^ and one study was rated high or probably high for only one domain^79^ (see Supporting Information Files 5 and 6).

For the microplastic type, three studies tested polystyrene microplastics^68,76,79^ and two studies tested polyethylene microplastics.^75,78^

Cytokines such as tumor necrosis factor-α (TNF-α), IL-2, IL-6, IL-5, IL-9, IL-10, IP-10, G-CSF, iLb, Rantes, and IL-1α were measured in multiple studies. TNF-α levels significantly increased in the colon^76^ and the intestine.^68^ In two studies, TNF-α levels were not significantly different regardless of the exposed group in colon and small intestine.^75,79^ The level of IL-6 also significantly increased in the colon^76^ and all^75,79^ or part^68^ of the small intestine. The level of IL-10 (anti-inflammatory cytokine) significantly decreased in the colon^76^ but not in intestinal serum.^78^ There was no significant change in Ilb in the intestine in one study.^79^ IL-1α levels significantly increased in the intestine in two studies.^68,78^ For one study, there are two proteins related to inflammation (iNOS and COX-2) with levels that were significantly increased in the exposure group compared to the control.^68^ Eight other cytokines were measured in specific studies, and most of them had significant changes (increase or decrease, depending on the specific cytokine) between control and exposure groups. See Supporting Information File 4 (“Study Results”).

The estimate of the proportion of effects showing microplastics are harmful = 0.94 (95% CI of 0.80–0.98) (*n* = 30/32). See Supporting Information File 9 and Figure S2.

We conducted a sensitivity analysis and (1) compared the estimate of the proportion of effects showing polystyrene microplastics are harmful = 1.00 (95% CI of 0.76–1.00) (*n* = 12/12) versus polyethylene microplastics = 0.90 (95% CI of 0.70–97) (*n* = 18/20) (difference between proportions *p* = 0.26) and (2) measured the proportion of effects showing microplastics are harmful when only one result per study was considered = 1.00 (95% CI of 0.51–1.00) (*n* = 4/4) versus our primary analysis of including all study results from each study = 0.94 (95% CI of 0.80–0.98) (*n* = 30/32) (difference between proportions *p* = 0.61) (Supporting Information File 7).

We found that you could not reasonably distinguish between the polystyrene and the polyethylene results or when only one result per study was considered versus our primary analysis of including all study results from each study.

We concluded that exposure to microplastics is “suspected” to adversely impact intestinal chronic inflammation in humans on the basis of (a) the “moderate” quality of the body of evidence (see Supporting Information File 8, “Evidence ratings for studies”), (b) the direction of the effect (i.e., evidence of an increasing adverse health effect with an increasing level of microplastic exposure), and (c) the confidence in the association considering factors including the number and size of studies.

##### Immunosuppressive Effects

Two studies^75,79^ measured biomarkers related to the immune system, reporting significant changes in immunophenotype populations (CD4 T lymphocytes, CD8 T lymphocytes, CD3+CD8+ T cells, CD19+ lymphocytes and dendritic cells, and inflammatory monocytes), neutrophils (granulocytes in white blood cells), and anti-inflammatory macrophages (play a critical role in inflammation). Changes in cell populations may not directly relate to immunosuppression, but they do relate to the immune system and could produce an immunomodulatory effect. See Supporting Information File 4 (“Study Results”).

The estimate of the proportion of effects showing microplastics are harmful = 1.00 (95% CI of 0.70–1.00) (*n* = 9/9). See Supporting Information File 9 and Figure S3. We did not conduct a sensitivity analysis on the basis of microplastic type or inclusion of only one result per study as every result was in the direction of showing harm.

For the risk of bias, one study was rated high or probably high for two domains^75^ and one study was rated high or probably high^79^ for only one domain (see Supporting Information Files 5 and 6).

For the microplastic type, one study tested polystyrene microplastics^79^ and one study polyethylene microplastics.^75^

We concluded that exposure to microplastics is “suspected” to adversely impact intestinal immune system function in humans on the basis of (a) the “high” quality of the body of evidence (see Supporting Information File 8, “Evidence ratings for studies”), (b) the direction of the effect (i.e., evidence of an increasing adverse health effect with an increasing level of microplastic exposure), and (c) the confidence in the association considering factors including the number and size of studies.

##### Induction of Oxidative Stress

Four studies examined markers indicating increased levels of oxidative stress in the colon and intestine.^68,76,79,80^

For the risk of bias, one study was rated high or probably high for four domains,^68^ one study was rated high or probably high for three domains,^76^ and two studies were rated high or probably high for only one domain^79,80^ (see Supporting Information Files 5 and 6).

For the microplastic type, three studies tested polystyrene^68,76,79^ and one study polyethylene.^80^

Two studies^76,80^ found significant changes for glutathione in the colon and intestine, three studies malondialdehyde concentrations in the colon and intestine,^76,79,80^ and two an increase in reactive oxidative species in the intestine.^68,79^ See Supporting Information File 4 (“Study Results”).

The estimate of the proportion of effects showing microplastics are harmful = 1.00 (95% CI of 0.76–1.00) (*n* = 12/12).

We concluded that impacts of microplastic exposure on intestinal oxidative stress are “not classifiable” on the basis of (a) the “low” quality of the body of evidence (see Supporting Information File 8, “Evidence ratings for studies”), (b) the direction of the effect (i.e., evidence of an increasing adverse health effect with an increasing level of microplastic exposure), and (c) the confidence in the association considering factors including the number and size of studies.

##### Modulation of Receptor-Mediated Effects (hormones)

One study measured hormonal changes (specifically, cholecystokinin, or CCK, and gastrin) in the midcolon.^69^ Midcolonic concentrations of CCK, which is a peptide hormone responsible for the digestion of fat and protein, and gastrin, a hormone that stimulates gastric juice secretion, were significantly decreased. See Supporting Information File 4 (“Study Results”).

For the risk of bias, this study was rated high or probably high for four domains^69^ (see Supporting Information Files 5 and 6).

For the microplastic type, this study tested polystyrene microplastics.^69^

The estimate of the proportion of effects showing microplastics are harmful = 1.00 (95% CI of 0.34–1.00) (*n* = 2/2).

We concluded that impacts of microplastics exposure on digestive hormones are “not classifiable” on the basis of (a) the “low” quality of the body of evidence (see Supporting Information File 8, “Evidence ratings for studies”), (b) the direction of the effect (i.e., evidence of an increasing adverse health effect with an increasing level of microplastic exposure), and (c) the confidence in the association considering factors including the number and size of studies.

We considered the overall quality of the evidence for digestive outcomes as “moderate”. See Supporting Information File 8 (“Evidence ratings for studies”) for the detailed rationale for these ratings.

##### Conclusion about Digestive Studies

Across the outcomes, we identified that exposure to microplastics is “suspected” to be a digestive hazard to humans, including a suspected link to colon cancer, using the key characteristics of carcinogens approach.^53,81^

### Reproductive Results

#### Human Studies

We evaluated two outcomes across two studies related to the reproductive system.^35,36^

##### Growth Outcomes

Both studies evaluated the growth outcome birth weight, one finding a statistically significant correlation with microplastic load in the placenta and reduced birth weight^35^ and the other no difference with microplastic load in amniotic fluid.^36^ One study found a statistically significant correlation with microplastic load in the placenta and reduced birth length and head circumference.^35^ See Supporting Information File 4 (“Study Results”).

The estimate of the proportion of effects showing microplastics are harmful = 0.75 (95% CI of 0.30–0.95) (*n* = 3/4).

For the risk of bias, one study was rated high risk of bias for the domain of confounding and rated probably high risk of bias for knowledge of group assignments (blinding),^35^ and one study was rated probably high risk of bias for selection of study groups and knowledge of group assignments^36^ [see Supporting Information File 5 (“Risk of bias heat map”) and Supporting Information File 6 for justification of ratings].

We concluded that exposure to microplastics is “not classifiable” for birth outcomes in humans on the basis of (a) the “low” quality of the body of evidence (see Supporting Information File 8, “Evidence ratings for studies”, for a detailed rationale for these ratings), (b) the direction of the effect (i.e., evidence of an increasing adverse health effect with an increasing level of microplastic exposure), and (c) the confidence in the association considering factors including the number and size of studies.

##### Gestational Age

One study measured the associations between total microplastic abundance in maternal amniotic fluid and gestational age, finding a statistically significant decrease in age for a unit (particles per gram) increase in microplastics.^36^ See Supporting Information File 4 (“Study Results”).

The estimate of the proportion of effects showing microplastics are harmful = 1.00 (95% CI of 0.21–1.00) (*n* = 1/1).

For the risk of bias, this study was rated probably high risk of bias for the domains selection of study groups and knowledge of group assignments^36^ (see Supporting Information Files 5 and 6).

We concluded that exposure to microplastics is “not classifiable” for gestational age development in humans on the basis of (a) the “low” quality of the body of evidence (see Supporting Information File 8, “Evidence ratings for studies”), (b) the direction of the effect, and (c) the limited confidence in the association considering factors including the number and size of studies.

### Animal Studies

We evaluated 10 outcomes across 11 studies related to the reproductive system. Four studies^72,73,82,83^ evaluated female end points (including hormone level changes in the serum and ovaries and impacts to ovarian follicles), and five studies^74,84−87^ evaluated male end points (including sperm damage, testicular damage, and serum hormone level changes). One study evaluated oocyte meiotic progression/blatstocyst development.^82^ Four evaluated separate birth outcomes (weight of fetus and placenta, litter size, anogenital index, and distance).^82,86−88^ One study evaluated age at puberty.^87^ Studies that assessed hormone levels in the serum and ovaries were also included, as hormonal changes are a key characteristic of reproductive toxicants that may also impact reproductive health directly.^51,56,81^

Similar measurements were conducted between studies; however, not all measurements were the same, and estimates could not be combined in a meta-analysis or visually displayed collectively in a figure because estimates were reported on different scales, used different association metrics, or were not fully reported.

#### Apical Outcomes

##### Weight of Fetus and Placenta

One study^88^ evaluated birth outcomes by measuring the weight of the fetus and placenta. They found a statistically significant decrease in the weight of the fetus between the most and least exposed groups, but not for the weight of the placenta. See Supporting Information File 4 (“Study Results”).

The estimate of the proportion of effects showing microplastics are harmful = 0.50 (95% CI of 0.09–0.91) (*n* = 1/2).

For the risk of bias, this study was rated probably high for two domains (see Supporting Information Files 5 and 6).

For the microplastic type, this study tested polystyrene.

We concluded that exposure to microplastics is “not classifiable” for birth outcomes of the weight of the fetus and placenta on the basis of (a) the “low” quality of the body of evidence (see Supporting Information File 8, “Evidence ratings for studies”), (b) the direction of the effect, and (c) the limited confidence in the association considering factors including the number and size of studies.

##### Litter Size

Two studies^82,86^ evaluated the birth outcome of litter size. One study found a statistically significant difference in the number of offspring between the most and least exposed groups.^82^ One study found no difference in litter size or post-survival rate.^86^ See Supporting Information File 4 (“Study Results”).

The estimate of the proportion of effects showing microplastics are harmful = 0.33 (95% CI of 0.06–0.79) (*n* = 1/3).

For the risk of bias, both studies were rated high or probably high for three domains^82,86^ (see Supporting Information Files 5 and 6).

For the microplastic type, one study tested polystyrene^87^ and polyethylene.^82^

We concluded that exposure to microplastics is “not classifiable” for the birth outcome of litter size on the basis of (a) the “very low” quality of the body of evidence (see Supporting Information File 8, “Evidence ratings for studies”), (b) the direction of the effect, and (c) the limited confidence in the association considering factors including the number and size of studies.

##### Age at Puberty

One study^87^ evaluated age at puberty and found a statistically significant decrease in onset between the most and least exposed groups. See Supporting Information File 4 (“Study Results”).

The estimate of the proportion of effects showing microplastics are harmful = 1.00 (95% CI of 0.21–1.00) (*n* = 1/1).

For the risk of bias, this study was rated probably high for one domain^87^ (see Supporting Information Files 5 and 6).

For the microplastic type, this study tested polystyrene.^87^

We concluded that exposure to microplastics is “not classifiable” for the onset of puberty on the basis of (a) the “low” quality of the body of evidence (see Supporting Information File 8, “Evidence ratings for studies”), (b) the direction of the effect, and (c) the limited confidence in the association considering factors including the number and size of studies.

##### Oocyte Meiotic Progression/Blatstocyst Development

One study^82^ evaluated oocyte meiotic progression/blatstocyst development and found a statistically significant percentage decrease in both outcomes between the least and most exposed groups. See Supporting Information File 4 (“Study Results”).

The estimate of the proportion of effects showing microplastics are harmful = 1.00 (95% CI of 0.34–1.00) (*n* = 2/2).

For the risk of bias, this study was rated high or probably high for three domains^82^ (see Supporting Information Files 5 and 6).

For the microplastic type, this study tested polyethylene.^82^

We concluded that exposure to microplastics is “not classifiable” for effect meiotic progression/blatstocyst development on the basis of (a) the “low” quality of the body of evidence (see Supporting Information File 8, “Evidence ratings for studies”), (b) the direction of the effect, and (c) the limited confidence in the association considering factors including the number and size of studies.

##### Testicular Aging

One study^89^ measured testicular aging across seven measures and saw a consistent statistically significant effect in each one. See Supporting Information File 4 (“Study Results”).

The estimate of the proportion of effects showing microplastics are harmful = 1.00 (95% CI of 0.65–1.00) (*n* = 7/7).

For the risk of bias, this study was rated high or probably high for three domains (see Supporting Information Files 5 and 6).

For the microplastic type, this study tested polystyrene microplastics.

We concluded that exposure to microplastics is “not classifiable” for testicular aging on the basis of (a) the “very low” quality of the body of evidence (see Supporting Information File 8, “Evidence ratings for studies”), (b) the direction of the effect, and (c) the limited confidence in the association considering factors including the number and size of studies.

##### Anogenital Index and Distance

One study^87^ measured anogenital index and distance in two sets of pups, postnatal day 35 and 70, and found no significant difference between the least and most exposed groups for either end point or postnatal day. See Supporting Information File 4 (“Study Results”).

The estimate of the proportion of effects showing microplastics are harmful = 0.50 (95% CI of 0.15–0.85) (*n* = 2/4).

For the risk of bias, this study was rated probably high for one domain^87^ (see Supporting Information Files 5 and 6).

For the microplastic type, this study tested polystyrene.

We concluded that exposure to microplastics is “not classifiable” for anogenital index and distance on the basis of (a) the “very low” quality of the body of evidence (see Supporting Information File 8, “Evidence ratings for studies”), (b) the direction of the effect, and (c) the limited confidence in the association considering factors including the number and size of studies.

##### Sperm Quality

Five studies evaluated the effects of microplastic exposure on sperm and sperm-related outcomes.^74,84−87^

For the risk of bias, one study was rated high or probably high for four and three domains (different apical outcomes and/or results with different ROB ratings),^85^ one study was rated high or probably high for three domains,^86^ one study was rated high or probably high for three and two domains (different apical outcomes and/or results with different ROB ratings),^74^ one study was rated high or probably high for two domains and one domain (different apical outcomes and/or results with different ROB ratings),^84^ and one study was rated probably high for only one domain^87^ (see Supporting Information Files 5 and 6).

For the microplastic type, all five studies tested polystyrene.^74,84−87^

Studies found trends in declines in living sperm, sperm concentrations, and sperm motility as well as increases in sperm malformation (also reported as sperm deformity or sperm abnormality). Outcome assessors were blinded during sperm malformations and viability assessments in only one study.^84^ All studies reported positive associations between increasing microplastic exposure and decreases in measures of sperm quality and/or quantity. See Supporting Information File 4 (“Study Results”).

The estimate of the proportion of effects showing microplastics are harmful = 1.00 (95% CI of 0.70–1.00) (*n* = 9/9). See Supporting Information File 8 and Figure S4. We did not conduct a sensitivity analysis as all studies were in the direction of showing harm.

We concluded that exposure to microplastics is “suspected” to adversely impact sperm quality and testicular health in humans on the basis of (a) the “high” quality of the body of evidence (Supporting Information File 8, “Evidence ratings for studies”), (b) the direction of the effect (i.e., evidence of an increasing adverse health effect with an increasing level of microplastic exposure), and (c) the confidence in the association considering factors including the number and size of studies. See Supporting Information File 7 (“Evidence ratings for studies”) for a detailed rationale for these ratings.

##### Germinal Cell Thickness

One study^84^ evaluated the effects of microplastic exposure on germinal cell thickness and found a significant decrease and dose–response effects between control and exposure groups.^84^ See Supporting Information File 4 (“Study Results”).

The estimate of the proportion of effects showing microplastics are harmful = 1.00 (95% CI of 0.21–1.00) (*n* = 1/1).

This study was rated high or probably high for two domains.

This study tested polystyrene.

We concluded that exposure to microplastics is “not classifiable” for germinal thickness on the basis of (a) the “low” quality of the body of evidence (see Supporting Information File 8, “Evidence ratings for studies”), (b) the direction of the effect, and (c) the limited confidence in the association considering factors including the number and size of studies.

##### Follicles/Ovarian Reserve Capacity

Two studies evaluated the effects of microplastic exposure on ovarian follicles.^72,73^

For the risk of bias, both studies were rated high or probably high for three domains^72,73^ (see Supporting Information Files 5 and 6).

For the microplastic type, both studies tested polystyrene.^72,73^

Both studies found a significant decrease in the number of growing follicles for the most exposed group and a consistent dose–response relationship. For both studies, five random visual fields were used to assess the number of growing follicles via microscope imaging for each rat model (six from each group). It is unclear whether five images were sufficient to qualitatively assess the measurement, but the authors do refer to previous literature for their methodology. See Supporting Information File 4 (“Study Results”).

The estimate of the proportion of effects showing microplastics are harmful = 1.00 (95% CI of 0.34–1.00) (*n* = 2/2). See Supporting Information File 9 and Figure S5. We did not conduct a sensitivity analysis as both studies tested polystyrene and each study contributed only one study result for the outcome.

We concluded that exposure to microplastics is “suspected” to adversely impact ovarian follicle development in humans on the basis of (a) the “moderate” quality of the body of evidence, (b) the direction of the effect (i.e., evidence of an increasing adverse health effect with an increasing level of microplastic exposure), and (c) the confidence in the association considering factors including the number and size of studies.

#### Biological Changes (key characteristics)

##### Reproductive Hormones

Six studies measured alterations of reproductive hormones.^72,73,82−84,87^

For the risk of bias, two studies were rated high or probably high for four domains,^82,83^ two studies were rated high or probably high for two domains,^72,73^ and two studies were rated high or probably high for one domain^84,87^ (see Supporting Information Files 5 and 6).

For the microplastic type, five studies tested polystyrene^72,73,83,84,87^ and one study tested polyethylene.^82^

Two studies found significant changes in anti-Müllerian hormone (AMH) concentration: one in serum^73^ and the other in ovaries.^72^ One study found significant changes in Inhibin in pups postnatal day 35 and 70.^87^ Four studies measured luteinizing hormone (LH),^82−84,87^ but only one found significant decreases in the level of serum LH.^84^ Two studies found no significant changes in progesterone.^82,83^ Three of four studies found significant changes in follicle-stimulating hormone (FSH).^83,84,87^ Three studies found significant changes in testosterone concentrations.^83,84,87^ See Supporting Information File 4 (“Study Results”).

The estimate of the proportion of effects showing microplastics are harmful = 0.77 (95% CI of 0.57–0.90) (*n* = 17/22). See Supporting Information File 9 and Figure S6.

We conducted a sensitivity analysis and (1) compared the estimate of the proportion of effects showing polystyrene microplastics are harmful = 0.78 (95% CI of 0.55–0.91) (*n* = 14/18) versus polyethylene microplastics = 0.75 (95% CI of 0.30–95) (*n* = 3/4) (difference between proportions *p* = 0.90) and (2) measured the proportion of effects showing microplastics are harmful when only one result per study was considered = 0.83 (95% CI of 0.44–0.97) (*n* = 5/6) versus our primary analysis of including all study results from each study = 0.77 (95% CI of 0.57–0.90) (*n* = 17/22) (difference between proportions *p* = 0.75) (Supporting Information File 7).

We found that you could not reasonably distinguish between the polystyrene and polyethylene results or when only one result per study was considered versus our primary analysis of including all study results from each study.

We concluded that exposure to microplastics is “suspected” to adversely impact reproductive hormones in humans on the basis of (a) the “moderate” quality of the body of evidence, (b) the direction of the effect (i.e., evidence of an increasing adverse health effect with an increasing level of microplastic exposure), and (c) the confidence in the association considering factors including the number and size of studies.

We considered the overall quality of the evidence for these outcomes as “moderate”. See Supporting Information File 8 (“Evidence ratings for studies”) for a detailed rationale for these ratings.

##### Conclusion about the Reproductive Studies

Across the outcomes that were fully evaluated, we identified that exposure to microplastics is “suspected” to be a hazard to the human reproductive system.

### Respiratory Results

#### Human Studies

We evaluated one study^91^ that measured the relationship between chronic rhinosinusitis without nasal polyps and microplastics and found a statistically significant difference in the level of microplastics in patients with chronic rhinosinusitis without nasal polyps compared to healthy volunteers.

The estimate of the proportion of effects showing microplastics are harmful = 1.00 (95% CI of 0.21–1.00) (*n* = 1/1).

For the risk of bias, this study was rated high for confounding and probably high for study group selection and exposure assessment (see Supporting Information File 5, “Risk of bias heat map”, and Supporting Information File 6 for justification of ratings).

We concluded that exposure to microplastics is “not classifiable” for chronic rhinosinusitis in humans on the basis of (a) the “very low” quality of the body of evidence, (b) the direction of the effect, and (c) the limited confidence in the association considering factors including the number and size of studies.

#### Animal Studies

We evaluated five outcomes across seven studies related to the respiratory system. Four studies^92−95^ evaluated total cell count (total cells, macrophages, lymphocytes, neutrophils, and polymorphonuclear cells). Three studies measured^92,93,95^ pulmonary function (pressure–volume loops, peak expiratory flows, tissue dampening, tissue elastance, central airway resistance, forced vital capacity, forced expiratory volume, tidal volume, minute volume, inspiratory time, expiratory time, peak inspiratory flow, and peak expiratory flow). Four studies^93−96^ evaluated lung injury (lung tissue score, pulmonary parenchymal area, average vessel thickness, and number of alveolar septa).

Three studies evaluated biomarkers related to chronic inflammation (IL-6 secretions, TNF-α secretions, IL-8 secretions, IL-1β secretions, TGF-β). Three studies^94,95,97^ evaluated biomarkers for lung fibrosis (vimentin, α-SMA, surfactant protein-C, MCP-1, and Krebs von den lungen-6 & KC) resulting from inflammation, and three studies^94,95,97^ evaluated biomarkers related to oxidative stress (ROS, SOD, GSH-PX, and CAT).

Similar measurements were conducted between studies; however, not all measurements were the same, and estimates could not be combined in a meta-analysis or visually displayed collectively in a figure because estimates were reported on different scales, used different association metrics, or were not fully reported.

#### Apical Outcomes

##### Pulmonary Function

Three studies^92,93,95^ evaluated pulmonary function (pressure–volume loops, peak expiratory flows, tissue dampening, tissue elastance, central airway resistance, forced vital capacity, forced expiratory volume, tidal volume, minute volume, inspiratory time, expiratory time, peak inspiratory flow, and peak expiratory flow) and found decreased forced vital capacity (FVC) and forced expiratory volume at 1 s (FEV_1_). See Supporting Information File 4 (“Study Results”).

The estimate of the proportion of effects showing microplastics are harmful = 0.83 (95% CI of 0.63–0.93) (*n* = 19/23). See Supporting Information File 9 and Figure S7.

For the risk of bias, three studies were rated high/probably high for two domains^92,93,95^ (see Supporting Information Files 5 and 6).

For the microplastic type, two studies tested polystyrene^92,93,95^ and one tested tire wear microplastic particles.^93^

We conducted a sensitivity analysis and (1) compared the estimate of the proportion of effects showing polystyrene microplastics are harmful = 0.73 (95% CI of 0.48–0.89) (*n* = 11/15) versus tire wear microplastics = 1.00 (95% CI of 0.68–1.00) (*n* = 8/8) (difference between proportions *p* = 0.11) and (2) measured the proportion of effects showing microplastics are harmful when only one result per study was considered = 1.00 (95% CI of 0.44–1.00) (*n* = 3/3) versus our primary analysis of including all study results from each study = 0.83 (95% CI of 0.63–0.93) (*n* = 19/23) (difference between proportions *p* = 0.43) (Supporting Information File 7).

We found polystyrene microplastics had a lower estimate of the proportion of effects showing harm versus tire wear microplastics; however, the difference was not statistically significant. We found when only one result per study was considered, the estimate of the proportion of effects showing microplastics are harmful was greater versus our primary analysis of including all study results from each study; however, the difference was not statistically significant.

We concluded that exposure to microplastics is “suspected” to adversely impact pulmonary function in humans on the basis of (a) the “moderate” quality of the body of evidence (see Supporting Information File 8, “Evidence ratings for studies”, for a detailed rationale for these ratings), (b) the direction of the effect (i.e., evidence of an increasing adverse health effect with an increasing level of microplastic exposure), and (c) the confidence in the association considering factors including the number and size of studies.

##### Lung Injury

Four studies^93−96^ evaluated lung injury (lung tissue score, pulmonary parenchymal area, average vessel thickness, number of alveolar septa, and alveolar epithelial hyperplasia) and found consistent effects indicating damage and fibrosis to the lung tissue. These findings are consistent with lung tissue damage. See Supporting Information File 4 (“Study Results”).

The estimate of the proportion of effects showing microplastics are harmful = 0.88 (95% CI of 0.53–0.98) (*n* = 7/8). See Supporting Information File 9 and Figure S8.

For the risk of bias, one study was rated high/probably high across four domains,^94^ for three domains,^93^ and two were rated high/probably high for two domains^95,96^ (see Supporting Information Files 5 and 6).

For the microplastic type, two studies tested polystyrene,^95,96^ one tested polypropylene,^94^ and one tested tire wear microplastic particles^93^

We conducted a sensitivity analysis and (1) compared the estimate of the proportion of effects showing polystyrene microplastics are harmful = 1.00 (95% CI of 0.44–1.00) (*n* = 3/3) versus polypropylene = 1.00 (95% CI of 0.21–1.00) (*n* = 1/1) and versus tire wear microplastics = 1.00 (95% CI of 0.51–1.00) (*n* = 4/4) and (2) measured the proportion of effects showing microplastics are harmful when only one result per study was considered = 0.75 (95% CI of 0.30–0.95) (*n* = 3/4) versus our primary analysis of including all study results from each study = 0.88 (95% CI of 0.53–0.98) (*n* = 7/8) (difference between proportions *p* = 0.57) (Supporting Information File 7).

We found no difference in the estimate of the proportion of effects showing harm between polystyrene versus polyethylene microplastics and between polystyrene versus tire ware microplastics. We found when only one result per study was considered, the estimate of the proportion of effects showing microplastics are harmful was lower versus our primary analysis of including all study results from each study; however, the difference was not statistically significant.

We concluded that exposure to microplastics is “suspected” to cause lung injury on the basis of (a) the “moderate” quality of the body of evidence (see Supporting Information File 8, “Evidence ratings for studies”), (b) the direction of the effect (i.e., evidence of an increasing adverse health effect with an increasing level of microplastic exposure), and (c) the confidence in the association considering factors including the number and size of studies.

##### Total Cell Counts

Four studies^92−95^ evaluated total cell counts (total cells, macrophages, lymphocytes, neutrophils, and polymorphonuclear cells).

For the risk of bias, one study was rated high/probably high across four domains,^94^ one was rated high/probably high for two domains,^95^ two were rated high/probably high for one domain^92^ (see Supporting Information Files 5 and 6).

For the microplastic type, two studies tested polystyrene,^92,95^ one tested polypropylene,^94^ and one tested tire wear microplastic particles.^93^ Two studies found a decrease in the number of macrophages that were statistically significant.^93,95^ Three studies found an increase in the total number of cells and lymphocytes.^93−95^ Two studies found a statistically significant increase in the number of neutrophils.^94,95^ See Supporting Information File 4 (“Study Results”).

The estimate of the proportion of effects showing microplastics are harmful = 0.74 (95% CI of 0.54–0.87) (*n* = 17/23). We concluded that impacts of microplastics exposure on total cell counts are “not classifiable” on the basis of (a) the “very low” quality of the body of evidence (see Supporting Information File 8, “Evidence ratings for studies”), (b) the direction of the effect (i.e., evidence of an increasing adverse health effect with an increasing level of microplastic exposure), and (c) the confidence in the association considering factors including the number and size of studies.

#### Biological Changes (key characteristics)

##### Chronic Inflammation

Five studies^92,94,95,98,99^ evaluated biomarkers related to chronic inflammation (IL-6 secretions, TNF-α secretions, IL-8 secretions, IL-1β secretions and TGF-β) and resultant lung fibrosis (vimentin, α-SMA, surfactant protein-C, Krebs von den lungen-6, and MCP-1) and found increased levels of measured biomarkers in mice exposed to microplastics consistent with inflammation and lung fibrosis. See Supporting Information File 4 (“Study Results”).

The estimate of the proportion of effects showing microplastics are harmful = 0.96 (95% CI of 0.82–0.99) (*n* = 27/28). See Supporting Information File 9 and Figure S9.

For the risk of bias, one study was rated high/probably high across four domains,^94^ one study was rated high/probably high in three domains,^97^ and three studies were rated high/probably high for two domains^92,95,98^ (see Supporting Information Files 5 and 6).

For the microplastic type, four studies tested polystyrene^92,95,98,97^ and one tested polypropylene^94^ microplastics.

We conducted a sensitivity analysis and (1) compared the estimate of the proportion of effects showing polystyrene microplastics are harmful = 0.96 (95% CI of 0.79–0.99) (*n* = 22/23) versus polypropylene microplastics = 1.00 (95% CI of 0.57–1.00) (*n* = 5/5) (difference between proportions *p* = 0.64) and (2) measured the proportion of effects showing microplastics are harmful when only one result per study was considered = 1.00 (95% CI of 0.57–1.00) (*n* = 5/5) versus our primary analysis of including all study results from each study = 0.96 (95% CI of 0.82–0.99) (*n* = 27/28) (difference between proportions *p* = 0.64) (Supporting Information File 7).

We found that you could not reasonably distinguish between the polystyrene and the polypropylene results or when only one result per study was considered versus our primary analysis of including all study results from each study.

We concluded that exposure to microplastics is “suspected” to induce chronic inflammation and lung fibrosis in humans on the basis of (a) the “moderate” quality of the body of evidence (see Supporting Information File 8, “Evidence ratings for studies”), (b) the direction of the effect (i.e., evidence of an increasing adverse health effect with an increasing level of microplastic exposure), and (c) the confidence in the association considering factors including the number and size of studies.

##### Oxidative Stress

Three studies^94,95,97^ evaluated biomarkers related to oxidative stress (ROS, SOD, GSH-PX, and CAT) and found that the decrease in the levels of SOD, GSH/PX, and CAT and the increase in the level of ROS are consistent with oxidative stress in the lung. See Supporting Information File 4 (“Study Results”).

The estimate of the proportion of effects showing microplastics are harmful = 1.00 (95% CI of 0.70–1.00) (*n* = 9/9). See Supporting Information File 9 and Figure S10.

For the risk of bias, one study was rated high/probably high across four domains,^94^ one study was rated high/probably high in three domains,^97^ and one study was rated high/probably high for two domains^95^ (see Supporting Information Files 5 and 6). We did not conduct a sensitivity analysis as every result was in the direction of showing harm.

For the microplastic type, two studies tested polystyrene^95,97^ and one tested polypropylene^94^ microplastics.

We concluded that exposure to microplastics is “suspected” to induce oxidative stress on the basis of (a) the “moderate” quality of the body of evidence (see Supporting Information File 8, “Evidence ratings for studies”), (b) the direction of the effect (i.e., evidence of an increasing adverse health effect with an increasing level of microplastic exposure), and (c) the confidence in the association considering factors including the number and size of studies.

We considered the overall quality of the evidence for these outcomes as “moderate” quality. See Supporting Information File 7 (“Evidence ratings for studies”) for a detailed rationale for these ratings.

##### Conclusion about the Respiratory Studies

Across the outcomes that were fully evaluated, we identified that exposure to microplastics is “suspected” to be a hazard to the human respiratory system.

## Discussion

We have identified suspected human health risks from microplastic exposure in three body systems (digestive, reproductive, and respiratory). For reproductive outcomes (sperm quality) and digestive outcomes (immunosuppression) we rated the overall body of evidence as “high” quality and concluded microplastic exposure is “suspected” to adversely impact them based on consistent evidence of adverse health effects and confidence in the association. We downgraded the evidence from “presumed” based on the sample size and number of studies. For reproductive outcomes (female follicles and reproductive hormones), digestive outcomes (gross or microanatomic colon/small intestine effects, alters cell proliferation and cell death, and chronic inflammation), and respiratory outcomes (pulmonary function, lung injury, chronic inflammation, and oxidative stress) we rated the overall body of evidence as “moderate” quality and concluded microplastic exposure is “suspected” to adversely impact them based on consistent evidence of adverse health effects and confidence in the association. We concluded that exposure to microplastics is “unclassifiable” for birth outcomes and gestational age in humans based on the “low” and “very low” quality of the evidence.

Given the ubiquity of microplastics and the consistent, growing recognition of their existence in the human body, it is likely that microplastics will impact other body systems, which is a potential area for future research.^100^ This is a particularly timely given that plastic production is projected to triple by 2060.^2^

These findings have important implications for policy and research. First, given the indication of harm that we have identified, the need for additional research on the health effects of microplastics should not preclude action. We strongly recommend that regulatory agencies and decision makers can act on limited evidence given that evidence has been shown to grow and get stronger^101^ and initiate actions to prevent or mitigate human exposure to microplastics. Second, there is opportunity under the U.S. Environmental Protection Agency’s Toxic Substances Control Act (TSCA) to consider microplastics as a class or category of chemicals^102^ in its risk evaluations, which is a key component of identifying health risks for risk management actions. The U.S. Congress gave the U.S. Environmental Protection Agency (EPA) the authority to jointly evaluate any “category of chemical substances”,^103^ defined as “a group of chemical substances the members of which are similar in molecular structure, in physical, chemical, or biological properties, in use, or in mode of entrance into the human body or into the environment, or the members of which are in some other way suitable for classification.”^104^ Microplastics would meet this definition. Additionally, EPA could conduct a cumulative risk assessment based on their draft approach.^105^

The strengths of this work include the use of established rapid systematic review (rapid review) methods to accelerate the process of performing a full systematic review.^38,39^ Our rapid review was guided by the Navigation Guide systematic review method,^40^ which has been implemented to evaluate the health effects of multiple chemical exposures^41−43,106^ and used by the World Health Organization and International Labor Organization Joint Estimates of the Work-related Burden of Disease and Injury.^44^ These methods represent a transparent, rigorous, and unbiased approach to gathering the available evidence, evaluating it, and developing actionable statements for decision makers.

We applied the key characteristics approach,^51,53,56^ an approach that is in alignment with the State of California’s current efforts to advance methods using biological and mechanistic data to understand human health harms from exposure to chemicals.^107^ For the digestive and respiratory outcomes, we utilized the key characteristics of carcinogens. For reproductive health outcomes, we utilized the key characteristics of reproductive toxicity.^51,56^ We used the concept of key characteristics to identify mechanisms indicative of cancer or reproductive toxicity.^51,53,56,81^ Using this approach, the greater the number of key characteristics identified, the more likely the exposure (microplastics) was linked to these adverse health outcomes. We prioritized the evidence most useful for understanding the impacts of microplastic exposure on human health and reported significant findings on the basis of statistical relevance. We conducted a sensitivity analysis to test the robustness of our results when including only one type of microplastic and only one study result per outcome in the synthesis.

We extrapolated microplastic exposure concentrations in rodent studies to the predicted exposure concentrations in humans. We converted all microplastic concentrations (which were reported in a variety of ways, including micrograms per liter, micrograms, milligrams per kilogram, micrograms per gram, and milligrams per day) to particles per liter for water or particles per gram for food. Assuming an approximate daily consumption rate of 5 mL of water and 5 g of food for each rodent, a daily microplastic consumption rate was estimated unless specified otherwise.^108^ To convert the units from mass to particles, we assumed a spherical shape and density of each plastic polymer under standard conditions (1.05 g/cm^3^ for polystyrene and 0.96 g/cm^3^ for polyethylene).^109^

For microplastic sizes between 5 and 150 μm, the range of daily microplastic intake for exposed rodent experiments is approximately 7–70 000 microplastic particles, which is in range with the estimated daily microplastic intake for humans (∼422 particles per day).^110^ For smaller microplastic sizes such as 0.05–0.5 μm, the range of daily exposure concentrations was approximately 7 × 10^6^ to 8.02 × 10^11^, which could be higher than estimated human exposure concentrations but can still be informative for human health effects.

There were both methodological limitations and evidence base limitations of this review. Although the methods we employed were extremely rigorous, we recognize the possibility for increased human error, particularly in our screening and risk of bias assessment methods in which one person was screened/evaluated and another verified, which would be conducted in duplicate in a full systematic review. We also did not evaluate all outcomes reported in the included studies, nor did we consider all body systems that may be impacted by microplastic exposure. We further recognize that we were addressing only rodent studies and that the inclusion of other species (such as zebrafish) would make our findings more robust. Additionally, the use of *p* values to identify if there was a significant harmful difference between the control and most exposed group is likely to underestimate the number of outcomes where microplastic exposure leads to changes between these groups.^62^ However, we avoided placing increased weight on statistical significance, which does not address biological significance or the magnitude of the effect observed.

Despite the growing body of evidence linking microplastics to adverse health outcomes, limitations in the evidence base remain. The studies in our rapid review are limited to primary microplastics of only three polymer types (polystyrene, polyethylene, and polypropylene) and one source of secondary microplastics (tire wear particles). The shape and size of microplastics evaluated in the included studies were also very homogeneous (generally spherically shaped). The variety of microplastics in terms of polymer types, sizes, and shapes is much greater and may differentially impact health but has not been studied in chronic rodent systems.^111^ We also could not account for additives in the plastics or the effects of microplastics degraded from sources like fabrics given the lack of studies on these topics. We could also not consider aggregate or cumulative exposures to microplastics and other environmental contaminants. We also did not consider the biological contaminants that may attach to microplastics,^112^ which may impact how other environmental chemicals or other biological contaminants enter the human body. Our study was also limited by the study population; only one study each evaluated sensitive life stages (e.g., child development), exacerbation by other stressors (e.g., poverty and food scarcity), or disease or genetic status (e.g., only healthy homogeneous rodents evaluated). Thus, we could not evaluate cumulative impacts of microplastic exposure.

There is a potential for publication biases. It is possible that studies showing null effects of microplastic exposure were either not accepted or submitted for publication or that other important end points in the included studies were either not measured or not reported. We additionally found limited human studies, which could reflect a lack of appropriate resource allocation to address the challenges of conducting epidemiological studies, or that this is a nascent area of research and that the follow-up time required to show the relationship between microplastics and human health effects has not been sufficient for these studies to be published. As this was a rapid review, we did not contact the authors for missing data.

Given these limitations, it is likely our conclusions underestimate the true health impacts of microplastic exposure. Importantly, these limitations highlight that there are clear opportunities for future research, including (1) epidemiological studies and standardizing analytical methods investigating the health impacts of microplastic exposure, (2) other health outcomes impacted by microplastic exposure, and (3) evaluating the impact of microplastic exposure for susceptible human populations due to their developmental stage or other socioenvironmental stressors. Finally, research should focus on identifying, and then evaluating, strategies for mitigating or preventing exposures to microplastics.

## Conclusion

Microplastics are “suspected” to harm human reproduction and digestive and respiratory health, with a suggested link to colon cancer. Future research on microplastics should investigate additional health outcomes impacted by microplastic exposure and identify strategies to reduce exposure. Governments at all levels of jurisdiction (federal, state, and local) should take immediate action to mitigate exposure from microplastics.

## Supporting Information Available

Identification of studies via databases and registers (PDF)
Outline of the 111 studies that were excluded after reviewing the full text along with a rationale for their exclusion (XLSX)
Information about all of the studies from which data were extracted (*n* = 31) (XLSX)
Information about study results for the digestive (*n* = 7), reproductive (*n* = 6), and respiratory (*n* = 5) studies that exposed their test subjects (rodents) to multiple concentrations of microplastics (XLSX)
Risk of bias heat maps for a summary of risk of bias judgments (PDF)
Microplastic risk of bias ratings and justifications (PDF)
Supporting Information File 7 (XLSX)
Quality ratings for the body of evidence by selected outcome for included digestive and reproductive studies (XLSX)
Graphical display of results (PDF)

## Footnotes

a As described in Materials and Methods, while we searched for studies with any health effects, due to the time restrictions of the project, we made transparent decisions to select only a few outcomes for evaluation in this study.
