# Supplementary figures and images for: Effects of Microplastic Exposure on Human Digestive, Reproductive, and Respiratory Health: A Rapid Systematic Review
Source: Environ Sci Technol. 2024 Dec 18;58(52):22843–64. doi: 10.1021/acs.est.3c09524 (PMC11697325; doi:10.1021/acs.est.3c09524)

## Identification of studies via databases and registers

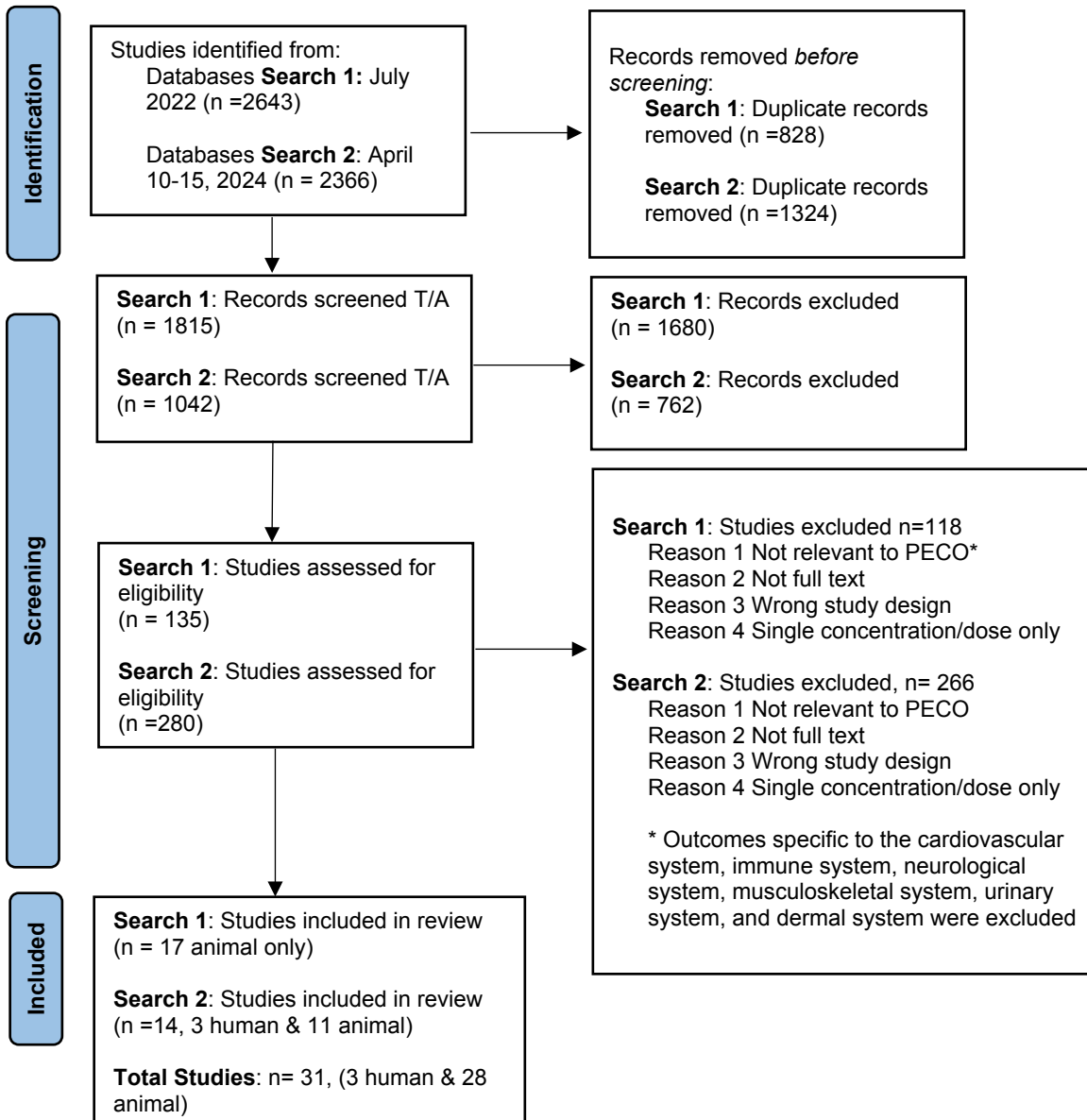

Supplement: Supplementary file 1 — es3c09524_si_001.pdf [file es3c09524_si_001.pdf]
