# Supplementary material for: Effects of Microplastic Exposure on Human Digestive, Reproductive, and Respiratory Health: A Rapid Systematic Review
Source: Environ Sci Technol. 2024 Dec 18;58(52):22843–64. doi: 10.1021/acs.est.3c09524 (PMC11697325; doi:10.1021/acs.est.3c09524)
Supplement: Supplementary file 5 — es3c09524_si_005.pdf [file es3c09524_si_005.pdf]

## Supplementary File 5. Risk of Bias Heat Map for summary of risk of bias judgments

### Risk of Bias Heat Map for Digestive Studies

| | Sequence Generation | Allocation Concealment | Blinding of Personnel & Outcome Assessors | Incomplete Outcome Data | Selective Outcome Reporting | Conflicts of Interest | Other Potential Threats to Validity – Outcome Evaluation |
|---|---|---|---|---|---|---|---|
| Jin et al., 2019 | + | - | - | ++ | + | + | + |
| Lu et al., 2018 | + | - | - | ++ | + | + | + |
| Li, B et al., 2020 | - | - | - | ++ | + | ++ | + |
| Chen, S et al. 2022 | + | - | + | + | + | ++ | + |
| Choi et al., 2021a | - | - | - | - | + | ++ | + |
| Choi et al., 2021b | - | - | - | - | + | ++ | + (n=1)[a]; - (n=5)[b] |
| Deng et al 2022 | + | + | - | + | + | ++ | + |
| Djouina et al., 2022 | + | - | - | + | + | ++ | + |
| Li et al. 2024 | + | - | ++ | + | + | ++ | + |
| Wen et al., 2022 | + | - | - | + | + | ++ | + (n=2)[c]; - (n=5)[d] |

*Notes:* ++ indicates low, + indicates probably low, - indicates probably high, -- indicates high.

a) Modulates receptor-mediated effects: CCK concentration, Gastrin concentration (mid colon)

b) Mucosa thickness, Muscle thickness, Flat luminal surface thickness, Crypt layer thickness (mid colon), charcoal transit ratio (mid colon); Intestine length (mid colon); Alters cell proliferation, cell death, or nutrient supply: Number of crypt of Lieberkuhn (mid colon); Alters cell proliferation, cell death, or nutrient supply: Goblet cell counts (mid colon)

c) Colon length; Induces chronic inflammation: Pro-inflammation cytokines (TNF-α, IL-6, and IL-10)

d) Alters cell proliferation, cell death, or nutrient supply: Goblet cell counts; Oxidative stress: Colonic glutathione (GSH); Oxidative stress: Superoxide dismutase (SOD); Oxidative stress: Malondialdehyde (MDA); Muscular layer width, Crypt depth (colon), intestine (proximal)

## Risk of Bias Heat Map for Reproductive Studies: Animal

| | Sequence Generation | Allocation Concealment | Blinding of Personnel & Outcome Assessors | Incomplete Outcome Data | Selective Outcome Reporting | Conflicts of Interest | Other Potential Threats to Validity – Outcome Evaluation |
|---|---|---|---|---|---|---|---|
| Agaahei etal., 2021 | + | _ | _ | + | + | ++ | ++ |
| An et al., 2021 | + | - | - | + | + | ++ | + (n=1)[a]; - (n=1)[b] |
| J Hou et al., 2021 | + | - | - | + | + | ++ | + (n=1)[c]; - (n=1)[d] |
| Huang et al., 2022 | + | - | - | + | + | ++ | - |
| B Hou et al., 2021 | + | - | - | ++ | + | ++ | - (n=1)[e]; -- (n=1)[f] |
| Li et al., 2021 | + | - | - | ++ | + | ++ | + (n=1)[g]; - (n=2)[h] |
| Jin et al., 2022 | + | - | + | ++ (n=2)[i]; - (n=2)[j] | + | ++ | + (n=2)[k]; - (n=2)[l] |
| Saeed et al., 2022 | - | - | - | ++ | + | + | __ |
| Wu etal., 2023 | + | - | - | + | + | ++ | __ |
| Zhang et al., 2023 | + | - | - | __ | + | + | + (n=3)[m]; - (n=1)[n] |
| Zhao et al., 2023 | + | + | - | + | + | ++ | + |

*Notes:* ++ indicates low, + indicates probably low, - indicates probably high, -- indicates high.

a) Hormone level changes: AMH levels (ovaries)

b) Follicles/Ovarian reserve capacity: Number of growing follicles

c) Hormone level changes: AMH levels (pg/ml) IL-18 (pg/ml) IL-1β (pg/ml)

d) Follicles: Number of growing follicles

e) Sperm damage: Rate of living sperm (%)

f) Sperm damage: Malformation (%)

g) Sperm damage: Sperm motility (%)

h) Sperm damage: Sperm concentration (106/ml); Sperm Damage: Sperm abnormality (%)

i) Viability of sperm (%); Sperm damage: Sperm abnormality deformity (%)

j) Hormone level changes: Testosterone LH levels (ng/ml) FSH levels (ng/ml) Concentrations of testosterone in serum (ng/ml); Testicular Damage: Seminiferous tubular diameter, Germinal epithelium thickness

k) Sperm damage: Viability of sperm (%); Hormone level changes: Testosterone LH levels (ng/ml) FSH levels (ng/ml) Concentrations of testosterone in serum (ng/ml)

l) Sperm damage: Sperm abnormality (%); Grminal epithelium thickness

m) Apical (Oocyte meiotic progression), Apical (Blatstocyst development), Other, (Litter size)

n) Alters production and levels of reproductive hormones ((E2, P, FSH and LH)

## Risk of Bias Heat Map for Reproductive Studies: Human

| | Study Group | Knowledge of Group Assignments | Exposure Assessment | Outcome Assessment | Confounding | Outcome Data | Selective Outcome Reporting | Conflicts of Interest | Other Potential Threats to Validity – Outcome Evaluation |
|---|---|---|---|---|---|---|---|---|---|
| Amereh et al., 2022 | + | - | ++ | + | -- | ++ | + | ++ | + |
| Xu et al., 2024 | - | - | ++ | ++ | + | ++ | + | ++ | + |

## Risk of Bias Heat Map for Respiratory Studies: Animal

| | Sequence Generation | Allocation Concealment | Blinding of Personnel & Outcome Assessors | Incomplete Outcome Data | Selective Outcome Reporting | Conflicts of Interest | Other Potential Threats to Validity – Outcome Evaluation |
|---|---|---|---|---|---|---|---|
| Y. Li et al., 2022 | + | - | ++ (n=2)[a]; - (n=2)[b] | + | + | ++ | + (n=2)[c]; -- (n=1)[d] |
| Fan et al., 2022 | + | - | - | ++ | + | ++ | + |
| X. Li et al., 2022 | - | - | - | + | + | ++ | + |
| Lim et al., 2021 | + | + | - | ++ (n=1)[e]; + (n=2)[f] | + | ++ | + (n=2)[g]; -- (n=1)[h] |
| Woo etal., 2023 | - | - | - | -- | + | + | + |
| Wu etal. 2024 | + | - | - | ++ | + | ++ | + |
| Yang et al. 2024 | + | - | - | + | + | + | + |

*Notes:* ++ indicates low, + indicates probably low, - indicates probably high, -- indicates high.

a) Lung injury: Lung tissue score, pulmonary parenchymal area, average vessel thickness, number of alveolar septal; Induces chronic inflammation: Lung collagen area (%)

b) Pulmonary function: tissue damping (cm$H_2O$/mL) tissue elastance (cm$H_2O$/mL) central airway resistance (cm$H_2O$/mL) peak expiratory flow (mL/s) forced vital capacity (mL) forced expiratory volume (mL), forced expiratory volume at 1s (mL) FEV0.1/ FV; Cell count: Number of total cells, macrophages, lymphocytes, neutrophils in bronchoalveolar lavage fluid

c) Pulmonary function: tissue damping (cm$H_2O$/mL) tissue elastance (cm$H_2O$/mL) central airway resistance (cm$H_2O$/mL) peak expiratory flow (mL/s) forced vital capacity (mL) forced expiratory volume (mL), forced expiratory volume at 1s (mL) FEV0.1/ FV; Cell count: Number of total cells, macrophages, lymphocytes, neutrophils in bronchoalveolar lavage fluid; Lung injury: Pulmonary parenchymal area, average vessel thickness, number of alveolar septal

d) Lung injury: Lung tissue score; Induces chronic inflammation: Lung collagen area (%)

e) Pulmonary function: frequency (BPM) tidal volume (mL) minute volume (mL/min) inspiratory time (s) expiratory time (s) peak inspiratory flow (mL/s) peak expiratory flow (mL/s)

f) Cell count: total cell count, macrophage, polymorphonuclear, and lymphocytes in bronchoalveolar lavage fluid; Induces chronic inflammation: Lung inflammatory index (TGF-β, TNF-α, vimentin protein expression); Induces oxidative stress: total protein (mg/dL), albumin (mg/dL), lactate dehydrogenase (mg/dL) in bronchoalveolar lavage fluid; Induces chronic inflammation: Lung inflammatory index (TGF-β, TNF-α, vimentin protein expression)

g) Cell count: total cell count, macrophage, polymorphonuclear, and lymphocytes; Induces chronic inflammation: Lung inflammatory index in bronchoalveolar lavage fluid.

h) Pulmonary function: frequency (BPM) tidal volume (mL) minute volume (mL/min) inspiratory time (s) expiratory time (s) peak inspiratory flow (mL/s) peak expiratory flow (mL/s);

## Risk of Bias Heat Map for Respiratory Studies: Human

| | Study Groups | Knowledge of Group assignments | Exposure Assessment | Outcome Assessment | Confounding | Outcome Data | Selective Outcome Reporting | Conflicts of Interest | Other Potential Threats to Validity – Outcome Evaluation |
|---|---|---|---|---|---|---|---|---|---|
| Tas et al., 2023 | - | + | - | ++ | -- | ++ | + | + | + |
