# Supplementary material for: Effects of Microplastic Exposure on Human Digestive, Reproductive, and Respiratory Health: A Rapid Systematic Review
Source: Environ Sci Technol. 2024 Dec 18;58(52):22843–64. doi: 10.1021/acs.est.3c09524 (PMC11697325; doi:10.1021/acs.est.3c09524)
Supplement: Supplementary file 6 — es3c09524_si_006.pdf [file es3c09524_si_006.pdf]

Supplementary File 6. Microplastics Risk of Bias Ratings & Justification

Digestive Studies

**Jin et al. 2019**		
**Domain**	**Rating**	**Justification for rating**
Sequence Generation	Probably low	There is insufficient information about the sequence generation process to permit a judgment of low risk of bias. Study authors make a simple statement such as 'we randomly allocated', but do not provide details regarding specific random components used in the sequence generation process.
Allocation Concealment	Probably high	Study authors do not make any statement about allocation concealment and the review author does not find indirect evidence suggesting allocation concealment.
Blinding of personnel and outcome assessors	Probably high	Study authors do not make any statement about blinding and the review author does not find indirect evidence suggesting blinding.
Incomplete Outcome Data	Apical: AB-PAS/mucus secretion coverage ratio (colon): Low	Animals included in the analysis are exactly those who were randomized into the experiment. The number of animals allocated to treatment groups is reported for outcomes of interest and data are provided indicating adequate follow up of all animals from the beginning of the study.
Selective Outcome Reporting	Probably low	All of the study's pre-specified outcomes outlined in the methods, that are of interest in the review have been reported in the pre-specified way (i.e., the outcomes outlined in the methods section match what is reported in the results section and vice versa), and the number of animals analyzed for outcomes of interest is provided.
Conflict of Interest	Probably low	A conflict of interest statement denying financial interests is not provided, but associated funds and/or persons appear to be free of financial interests in study outcome and are unaffiliated with parties with a financial interest.
Other Potential Threats to Validity- Outcome Evaluation	Apical: AB-PAS/mucus secretion coverage ratio (colon): Probably low	Methods were clearly described. They did select multiple colon selections and image fields for statistical testing. Pixel intensity and ratio by an image analyzer was used to assess this measurement. Therefore, there is no sufficient evidence of high risk of bias.

**Lu et al. 2018**		
**Domain**	**Rating**	**Justification for rating**
Sequence Generation	Probably low	There is insufficient information about the sequence generation process to permit a judgment of low risk of bias, but study authors make a simple statement such as 'we randomly allocated', but do not provide details regarding specific random components used in the sequence generation process.
Allocation Concealment	Probably high	Study authors do not make any statement about allocation concealment and the review author does not find indirect evidence suggesting allocation concealment.
Blinding of personnel and outcome assessors	Probably high	Study authors do not make any statement about blinding and the review author does not find indirect evidence suggesting blinding.
Incomplete Outcome Data	Apical: Decreased mucin secretion (colon): Low	The number of animals allocated is reported and matches the number of animals reported for each outcome (i.e., no missing outcome data).
Selective Outcome Reporting	Probably low	All of the study's pre-specified outcomes outlined in the methods, that are of interest in the review have been reported in the pre-specified way (i.e., the outcomes outlined in the methods section match what is reported in the results section and vice versa), and the number of animals analyzed for outcomes of interest is provided.
Conflict of Interest	Probably low	A conflict of interest statement denying financial interests is not provided, but associated funds and/or persons appear to be free of financial interests in study outcome and are unaffiliated with parties with a financial interest.
Other Potential Threats to Validity – Outcome Evaluation	Apical: Decreased mucin secretion (colon): Probably low	Mucus coverage ratio was measured by 6 sections of AB-PAS staining in each group. The text does not specify how many mice per group. The pixels were determined by an image analyzer.

**B Li et al. 2020**		
**Domain**	**Rating**	**Justification for rating**

Sequence Generation	Probably high	All of the study's pre-specified (primary and secondary) outcomes outlined in the methods, that are of interest in the review have been reported in the pre-specified way (i.e., the outcomes outlined in the methods section match what is reported in the results section and vice versa), and the number of animals analyzed for outcomes of interest is provided.
Allocation Concealment	Probably high	Study authors do not make any statement about allocation concealment and the review author does not find indirect evidence suggesting allocation concealment.
Blinding of personnel and outcome assessors	Probably high	Study authors do not make any statement about blinding and the review author does not find indirect evidence suggesting blinding.
Incomplete Outcome Data	Induces chronic inflammation: IL-1 α, G-CSF, IL-2, IL-5, IL-6, IL-9, IP-10 and RANTES IL-6, IL-10, IL-1 (intestine): Low	The number of animals allocated is reported and matches the number of animals reported for each outcome (i.e., no missing outcome data).
Selective Outcome Reporting	Probably low	All of the study's pre-specified (primary and secondary) outcomes outlined in the methods, that are of interest in the review have been reported in the pre-specified way (i.e., the outcomes outlined in the methods section match what is reported in the results section and vice versa), and the number of animals analyzed for outcomes of interest is provided.
Conflict of Interest	Low	The study did not receive support from a company, study author, or other party having a financial interest in the outcome of the study. A conflict of interest statement is provided to indicate the study authors have no financial interests and there is evidence of the parties not having a financial interest.
Other Potential Threats to Validity – Outcome Evaluation	Induces chronic inflammation: \ IL-1 α, G-CSF, IL-2, IL-5, IL-6, IL-9, IP-10 and RANTES: Probably low	IL-1 α, G-CSF, IL-2, IL-5, IL-6, IL-9, IP-10 and RANTES are a standard biomarkers for inflammation. They are measured either by ELISA kits or qt-PCR. All authors followed standard procedure to measure the biomarkers with enough samples to perform statistical analysis. Therefore, there is no evidence of potential high risk of bias.

Choi et al. 2021a		
**Domain**	**Rating**	**Justification for rating**
Sequence Generation	Probably high	Study authors do not make any statement about sequence generation and the review author does not find indirect evidence suggesting random sequence generation.
Allocation Concealment	Probably high	Study authors do not make any statement about allocation concealment and the review author does not find indirect evidence suggesting allocation concealment.
Blinding of personnel and outcome assessors	Probably high	Study authors do not make any statement about blinding and the review author does not find indirect evidence suggesting blinding.
Incomplete Outcome Data	Induces chronic inflammation: TNF-α, IL-1α,IL-6, iNOS, COX-2, NF-kB (intestine): Probably high	Study authors do not report numbers of animals allocated to treatment groups, but do provide data indicating adequate follow up for a subset of animals)
	Induces oxidative stress: SOD activity, SOD expression relative levels (intestine): Probably high	Study authors do not report numbers of animals allocated to treatment groups, but do provide data indicating adequate follow up for a subset of animals
	Induces oxidative stress: ROS concentration (intestine): Probably high	Study authors do not report numbers of animals allocated to treatment groups, but do provide data indicating adequate follow up for a subset of animals
Selective Outcome Reporting	Probably low	All of the study's pre-specified (primary and secondary) outcomes outlined in the methods, that are of interest in the review have been reported in the pre-specified way (i.e., the outcomes outlined in the methods section match what is reported in the results section and vice versa), and the number of animals analyzed for outcomes of interest is provided.
Conflict of Interest	Low	The study did not receive support from a company, study author, or other party having a financial interest in the outcome of the study. A conflict of interest statement is provided to indicate the

		study authors have no financial interests and there is evidence of the parties not having a financial interest.
Other Potential Threats to Validity – Outcome Evaluation	Induces chronic inflammation: TNF-α, IL-1α,IL-6, iNOS, COX-2, NF-kB (intestine): Probably low	TNF, IL-6, and IL-1α are standard biomarkers for inflammation. They are measured either by ELISA kits or qt-PCR. All authors followed standard procedure to measure the biomarker with enough samples to perform statistical analysis. Therefore, there is no evidence of potential high risk of bias
	Induces oxidative stress: SOD activity, SOD expression relative levels (intestine): Probably low	SOD measurement was performed by commercial kit. There is no evidence of potential high risk of bias.
	Induces oxidative stress: ROS concentration, (intestine): Probably low	ROS measurement was performed by commercial kit. There is no evidence of potential high risk of bias.

**Choi et al. 2021b**		
**Domain**	**Rating**	**Justification for rating**
Sequence Generation	Probably high	Study authors do not make any statement about sequence generation and the review author does not find indirect evidence suggesting random sequence generation.
Allocation Concealment	Probably high	Study authors do not make any statement about allocation concealment and the review author does not find indirect evidence suggesting allocation concealment.
Blinding of personnel and outcome assessors	Probably high	Study authors do not make any statement about blinding and the review author does not find indirect evidence suggesting blinding.
Incomplete Outcome Data	Apical: Mucosa thickness, Muscle thickness, Flat luminal	Study authors do not report numbers of animals allocated to treatment groups, but do provide data indicating adequate follow up for a subset of animals.

	surface thickness, Crypt layer thickness (mid colon): Probably high	
	Modulates receptor-mediated effects: CCK concentration, Gastrin concentration (mid colon): Probably high	Study authors do not report numbers of animals allocated to treatment groups, but do provide data indicating adequate follow up for a subset of animals.
	Apical: Charcoal transit ratio (mid colon): Probably high	Study authors do not report numbers of animals allocated to treatment groups, but do provide data indicating adequate follow up for a subset of animals.
	Apical: Intestine length (mid colon): Probably high	Study authors do not report numbers of animals allocated to treatment groups, but do provide data indicating adequate follow up for a subset of animals.
	Alters cell proliferation, cell death, or nutrient supply: Number of crypt of Lieberkuhn (mid colon): Probably high	Study authors do not report numbers of animals allocated to treatment groups, but do provide data indicating adequate follow up for a subset of animals.
	Alters cell proliferation, cell death, or nutrient supply: Goblet cell counts (mid colon): Probably high	Study authors do not report numbers of animals allocated to treatment groups, but do provide data indicating adequate follow up for a subset of animals.
Selective Outcome Reporting	Probably Low	All of the study's pre-specified outcomes outlined in the protocol, methods, abstract, and/or introduction that are of interest in the review have been reported in the pre-specified way, but study authors only report a subset of animals were examined for outcome of interest.
Conflict of Interest	Low	The study did not receive support from a company, study author, or other party having a financial interest in the outcome of the study. A conflict of interest statement is provided to indicate the

		study authors have no financial interests and there is evidence of the parties not having a financial interest.
Other Potential Threats to Validity – Outcome Evaluation	Apical: Mucosa thickness, Muscle thickness, Flat luminal surface thickness, Crypt layer thickness (mid colon): Probably high	H&E stained sections of mid-colon were used to measure the histopathological parameters. They were measured in duplicate for each slide. 4 to 6 mice per group was used for this analysis. They have examples of the measurements in the paper. However, it looks like the measurements can fluctuate depending on where the parameter is measured within the slide. This could lead to a high risk of bias.
	Modulates receptor-mediated effects: CCK concentration, Gastrin concentration (mid colon): Probably low	Hormone measure was performed on ELISA kit. There is not a 100% certainty of low risk of bias but there is no evidence of high risk of bias for this measurement.
	Apical: Charcoal transit ratio (mid colon): Probably high	The charcoal transit ratio is measured by light microscopy and measuring the 'charcoal' section of the intestine. They do show example light images in the publication but it is ambiguous when the 'charcoal' section begins or ends. There is no scale bar for comparison. The intestinal length is also not stretched completely in the image.
	Apical: Intestine length (mid colon): Probably high	Similar criticism as the charcoal transit ratio measurement. All measurements were done via optical imaging. The image does not stretch the intestine all the way and no measurement metric for comparison.
	Alters cell proliferation, cell death, or nutrient supply: Number of crypt of Lieberkuhn (mid colon): Probably high	The samples were prepped by fixing thin tissue sections onto grids and the measurement was done by TEM (transmission electron microscopy). The authors did not explicitly say the number of tissue sections or blinding in the text so there could be selective bias.
	Alters cell proliferation, cell death, or nutrient supply: Goblet cell counts (mid colon): Probably high	H&E stained sections of mid-colon were used to measure the histopathological parameters. They were measured in duplicate for each slide. 4 to 6 mice per group was used for this analysis. They have examples of the measurements in the paper. The number of Goblet cells was also not normalized to any kind of area section of the tissue, therefore it is difficult to compare across trials. There's some evidence of potential high risk of bias but not definitive.

Djouina et al. 2022		
**Domain**	**Rating**	**Justification for rating**
Sequence Generation	Probably low	Study authors make a simple statement such as ‘we randomly allocated’, but do not provide details regarding specific random component used in the sequence generation process
Allocation Concealment	Probably high	Study authors do not make any statement about allocation concealment and the review author does not find indirect evidence suggesting allocation concealment.
Blinding of personnel and outcome assessors	Probably high	Study authors do not make any statement about blinding and the review author does not find indirect evidence suggesting blinding.
Incomplete Outcome Data	Induces chronic inflammation: mRNA quantification of inflammatory cytokines TNF-α, Ifng, Il6, and Il1b (distal & proximal small intestine & colon) : Probably low	Study authors report number of animals allocated to treatment groups, but do not provide data indicating adequate follow up for a subset of animals.
	Apical: Villus length (Distal and proximal small intestine): Probably low	Study authors report number of animals allocated to treatment groups, but do not provide data indicating adequate follow up for all animals, only a subset of animals.
	Villus/crypt ratio: Small intestine (proximal), Small intestine (distal): Probably low	Study authors report number of animals allocated to treatment groups, but do not provide data indicating adequate follow up for all animals, only a subset of animals
	Apical: AB/PAS positive area (Distal and proximal small intestine, colon – epithelium mucosal surface area) =: Probably low	Study authors report number of animals allocated to treatment groups, but do not provide data indicating adequate follow up for all animals, only a subset of animals.

	Apical: Mucosal surface area (Colon-(epithelium): Probably low	Study authors report number of animals allocated to treatment groups, but do not provide data indicating adequate follow up for all animals, only a subset of animals.
	Is immunosuppressive: CD4 T lymphocytes (Proximal small intestine): Probably low	Study authors report number of animals allocated to treatment groups, but do not provide data indicating adequate follow up for a subset of animals.
	Is immunosuppressive: CD8 T lymphocytes (proximal small intestine): Probably low	Study authors report number of animals allocated to treatment groups, but do not provide data indicating adequate follow up for a subset of animals.
	Is immunosuppressive: Dendritic cells (proximal small intestine): Probably low	Study authors report number of animals allocated to treatment groups, but do not provide data indicating adequate follow up for a subset of animals.
	Is immunosuppressive: Inflammatory monocytes: (proximal small intestine): Probably low	Study authors report number of animals allocated to treatment groups, but do not provide data indicating adequate follow up for a subset of animals.
	Is immunosuppressive: Neutrophils (colon) Probably low	Study authors report number of animals allocated to treatment groups, but do not provide data indicating adequate follow up for a subset of animals.
	Is immunosuppressive: Anti-inflammatory macrophages (colon): Probably low	Study authors report number of animals allocated to treatment groups, but do not provide data indicating adequate follow up for a subset of animals.
	Apical: Crypt depth: (proximal small intestine): Probably low	Study authors report number of animals allocated to treatment groups, but do not provide data indicating adequate follow up for all animals, only a subset of animals.
Selective Outcome Reporting	Probably low	All of the study's pre-specified outcomes outlined in the methods, abstract, and/or introduction that are of interest in the review have been reported in the pre-specified way, but study authors report the number of animals analyzed for outcomes of interest as a range or report values for which numbers of animals analyzed need to be calculated by the review author.
Conflict of Interest	Low	The study did not receive support from a company, study author, or other party having a financial interest in the outcome of the study. A conflict of interest statement is provided to indicate the study authors have no financial interests and there is evidence of the parties not having a financial interest.

Other Potential Threats to Validity-Outcome evaluation	Induces chronic inflammation: mRNA quantification of inflammatory cytokines TNF-α, Il6, Il-b (distal & proximal small intestine, & colon): Probably low	Standard biomarkers for inflammation. They are measured either by ELISA kits or qt-PCR. All authors followed standard procedure to measure the biomarker with enough samples to perform statistical analysis. Therefore, there is no evidence of potential high risk of bias
	Apical: Villus length: (proximal and distal small intestine)) Probably low	These measurements were performed by microscopy and imaging software. Representative tissue samples were fixed with 4% paraformeldehyde. These are typical routine methods for these apical endpoints. However, they did not mention any blinding which is critical for image-based measurements. They presented vague details of the number of tissues measured and number of mice measured in each group. However, "at least 5" mice is a sufficient amount for statistical testing.
	Apical: Villus/crypt ratio: (proximal and distal small intestine) Probably low	These measurements were performed by microscopy and imaging software. Representative tissue samples were fixed with 4% paraformeldehyde. These are typical routine methods for these apical endpoints. However, they did not mention any blinding which is critical for image-based measurements. They presented vague details of the number of tissues measured and number of mice measured in each group. However, "at least 5" mice is a sufficient amount for statistical testing.
	Apical: AB/PAS positive area (proximal and distal small intestine, colon – epithelium mucosal surface area) Probably low	These measurements were performed by microscopy and imaging software. Representative tissue samples were fixed with 4% paraformeldehyde. These are typical routine methods for these apical endpoints. However, they did not mention any blinding which is critical for image-based measurements. They presented vague details of the number of tissues measured and number of mice measured in each group. However, "at least 5" mice is a sufficient amount for statistical testing.
	Apical: Mucosal surface area (Colon- epithelium) Probably low	These measurements were performed by microscopy and imaging software. Representative tissue samples were fixed with 4% paraformeldehyde. These are typical routine methods for these apical endpoints. However, they did not mention any blinding which is critical for image-based measurements. They presented vague details of the number of tissues measured and number of mice measured in each group. However, "at least 5" mice is a sufficient amount for statistical testing.
	Is immunosuppressive: CD4 T lymphocytes (proximal small intestine) Probably low	Measurements were performed via cell isolation and flow cytometry with standard methodology. There is not a 100% certainty of low risk of bias but there is no evidence of high risk of bias for this measurement.

	Is immunosuppressive: CD8 T lymphocytes (Proximal small intestine) Probably low	Measurements were performed via cell isolation and flow cytometry with standard methodology. There is not a 100% certainty of low risk of bias but there is no evidence of high risk of bias for this measurement.
	Apical: Crypt depth (proximal small intestine, distal small intestine) Probably low	These measurements were performed by microscopy and imaging software. Representative tissue samples were fixed with 4% paraformeldehyde. These are typical routine methods for these apical endpoints. However, they did not mention any blinding which is critical for image-based measurements. They presented vague details of the number of tissues measured and number of mice measured in each group. However, "at least 5" mice is a sufficient amount for statistical testing.
	Is immunosuppressive: Inflammatory monocytes (proximal small intestine) Probably low	Measurements were performed via cell isolation and flow cytometry with standard methodology. There is not a 100% certainty of low risk of bias but there is no evidence of high risk of bias for this measurement.
	Is immunosuppressive: Neutrophils (colon) Probably low	Measurements were performed via cell isolation and flow cytometry with standard methodology. There is not a 100% certainty of low risk of bias but there is no evidence of high risk of bias for this measurement.
	Is immunosuppressive: Anti-inflammatory macrophages (colon) Probably low	Measurements were performed via cell isolation and flow cytometry with standard methodology. There is not a 100% certainty of low risk of bias but there is no evidence of high risk of bias for this measurement.

**Wen et al. 2022**		
**Domain**	**Rating**	**Justification for rating**
Sequence Generation	Probably low	Study authors make a simple statement such as ‘we randomly allocated’, but do not provide details regarding specific random component used in the sequence generation process.
Allocation Concealment	Probably high	Study authors do not make any statement about allocation concealment and the review author does not find indirect evidence suggesting allocation concealment.
Blinding of personnel and outcome assessors	Probably high	Study authors do not make any statement about blinding and the review author does not find indirect evidence suggesting blinding.

Incomplete Outcome Data	Alters cell proliferation, cell death, or nutrient supply: Goblet cell counts (colon): Probably low	Study authors report number of animals allocated to treatment groups, but do not provide data indicating adequate follow up for all animals, only a subset of animals.
	Oxidative stress: Colonic glutathione (GSH, SOD, MDA) (colon): Probably low	Study authors report number of animals allocated to treatment groups, but do not provide data indicating adequate follow up for all animals, only a subset of animals.
	Apical: Colon length (colon) Probably low	Study authors report number of animals allocated to treatment groups, but do not provide data indicating adequate follow up for all animals, only a subset of animals.
	Induces chronic inflammation: TNF-α, IL-6, and IL-10 (colon): Probably low	Study authors report number of animals allocated to treatment groups, but do not provide data indicating adequate follow up for all animals, only a subset of animals.
	Muscular layer width, Crypt depth (colon, proximal small intestine): Probably low	Study authors report number of animals allocated to treatment groups, but do not provide data indicating adequate follow up for all animals, only a subset of animals.
Selective Outcome Reporting	Probably Low	All of the study's pre-specified outcomes outlined in the methods, abstract, that are of interest in the review have been reported in the pre-specified way, but study authors only report a subset of animals were examined for outcome of interest
Conflict of Interest	Low	Funding source is limited to government, non-profit organizations, or academic grants funded by government, foundations and/or non-profit organizations without financial interest in the treatments studied
Other Potential Threats to Validity- Outcome Evaluation	Alters cell proliferation, cell death, or nutrient supply: Goblet cell counts (colon): Probably high	The muscular layer width, crypt depth was measured using ImageJ 6.0, goblet cells were counted under an optical microscope. There is no mention of blinding. N = 3 for each trial tested. There's no guidance for the reader on how they conclude their measurements. No example measurement images. There is some evidence of potentially high risk of bias, but not definitive.
	Oxidative stress: Colonic glutathione (GSH, SOD, MDA) (colon): Probably high	GSH, SOD, MDA measurements were performed by commercial kits but the liver and colon tissue samples were homogenized together. Therefore, there is some evidence of potential risk of bias because of the blending between liver and colon.
	Apical: Colon length (colon): Probably low	Colon shortening was measured by a ruler and microscope. Example colon was clearly laid out in one of the figures with a ruler for comparison. There is no sufficient evidence of potential risk of bias.

	Induces chronic inflammation: Pro-inflammation cytokines (TNF-α, IL-6, and IL-10) (colon): Probably low	Standard biomarkers for inflammation. They are measured either by ELISA kits or qt-PCR. All authors followed standard procedure to measure the biomarker with enough samples to perform statistical analysis. Therefore, there is no evidence of potential high risk of bias.
	Muscular layer width, Crypt depth (colon, proximal small intestine): Probably high	The muscular layer width, crypt depth was measured using ImageJ 6.0, goblet cells were counted under an optical microscope. N = 3 for each trial tested. There's no guidance for the reader on how they conclude their measurements. No example measurement images. There is some evidence of potentially high risk of bias, but not definitive

**Li et al. 2024**		
**Domain**	**Rating**	**Justification for rating**
Sequence Generation	Probably low	Study authors make a simple statement such as 'we randomly allocated', but do not provide details regarding specific random component used in the sequence generation process.
Allocation Concealment	Probably high	Study authors do not make any statement about allocation concealment and the review author does not find indirect evidence suggesting allocation concealment.
Blinding of personnel and outcome assessors	Low	Investigators report blinding of key study personnel;
Incomplete Outcome Data	Immunosuppressive: CD8 T lymphocytes, CD3+CD8+ T cells, CD19+ lymphocytes: Probably Low	Study authors report number of animals allocated to treatment groups, and provide data indicating adequate follow up for only a subset of animals that is equal across each treatment group.
	Oxidative stress: (GSH, SOD, MDA, ROS) (intestine): Probably low	Study authors report number of animals allocated to treatment groups, and provide data indicating adequate follow up for only a subset of animals that is equal across each treatment group.
	Induces chronic inflammation: TNF-α, IL-6, and IL-b (intestine): Probably low	Study authors report number of animals allocated to treatment groups, and provide data indicating adequate follow up for only a subset of animals that is equal across each treatment group.

Selective Outcome Reporting	Probably Low	All of the study's pre-specified outcomes outlined in the methods, abstract, that are of interest in the review have been reported in the pre-specified way, but study authors only report a subset of animals were examined for outcome of interest
Conflict of Interest	Low	Funding source is limited to government, non-profit organizations, or academic grants funded by government, foundations and/or non-profit organizations without financial interest in the treatments studied
Other Potential Threats to Validity-Outcome Evaluation	Immunosuppressive: CD8 T lymphocytes, CD3+CD8+ T cells, CD19+ lymphocytes: Probably Low	The lymphocytes T populations were analyzed by flow cytometry. Measured in a standard way. There is no evidence of potential high risk of bias.
	Oxidative stress: (GSH, SOD, MDA, ROS) (intestine): Probably low	Measured in a standard way. There is no evidence of potential high risk of bias.
	Induces chronic inflammation: TNF-α, IL-6, and IL-b (intestine): Probably low	Measured in a standard way. There is no evidence of potential high risk of bias.

**Deng et al. 2022**		
**Domain**	**Rating**	**Justification for rating**
Sequence Generation	Probably low	Study authors make a simple statement such as 'we randomly allocated', but do not provide details regarding specific random component used in the sequence generation process.
Allocation Concealment	Probably Low	Review author finds indirect evidence suggesting allocation concealment, but study authors do not provide details about how investigators were prevented from foreseeing assignment
Blinding of personnel and	Probably High	Study authors do not make any statement about blinding and the review author does not find indirect evidence suggesting blinding.

outcome assessors		
Incomplete Outcome Data	Probably Low	Study authors report number of animals allocated to treatment groups, but do not provide data indicating adequate follow up for a subset of animals
Selective Outcome Reporting	Probably Low	All of the study's pre-specified outcomes outlined in the methods, abstract, that are of interest in the review have been reported in the pre-specified way, but study authors only report a subset of animals were examined for outcome of interest
Conflict of Interest	Low	Funding source is limited to government, non-profit organizations, or academic grants funded by government, foundations and/or non-profit organizations without financial interest in the treatments studied
Other Potential Threats to Validity-Outcome Evaluation	Probably Low	Measured in a standard way. There is no evidence of potential high risk of bias.

**Chen, S et al. 2022**		
**Domain**	**Rating**	**Justification for rating**
Sequence Generation	Probably low	Study authors make a simple statement such as 'we randomly allocated', but do not provide details regarding specific random component used in the sequence generation process.
Allocation Concealment	Probably high	Study authors do not make any statement about allocation concealment and the review author does not find indirect evidence suggesting allocation concealment.
Blinding of personnel and outcome assessors	Low	The review author judges certain aspects of the outcome or outcome measurement are unlikely to be influenced by lack of blinding, but the review author does not feel confident enough to permit a low risk of bias judgment.
Incomplete Outcome Data	Oxidative stress: (GSH, MDA) (duodenum, jejunum, ileum,	Study authors report number of animals allocated to treatment groups, and provide data indicating adequate follow up for only a subset of animals that is equal across each treatment group.

	caecum, and colon): Probably low	
Selective Outcome Reporting	Probably Low	All of the study's pre-specified outcomes outlined in the methods, abstract, that are of interest in the review have been reported in the pre-specified way, but study authors only report a subset of animals were examined for outcome of interest
Conflict of Interest	Low	Funding source is limited to government, non-profit organizations, or academic grants funded by government, foundations and/or non-profit organizations without financial interest in the treatments studied
Other Potential Threats to Validity-Outcome Evaluation	Oxidative stress: (GSH, MDA) (duodenum, jejunum, ileum, caecum, and colon): Probably low	Used enzyme-linked immunosorbent assays (ELISA) kits (JM-03100 M1, JM-02912 M1, JM-11347 M1, and JM-03038 M1, Jiangsu Jingmei Biotechnology Co. Ltd.) and micro-plate readers (Infinite F50 and Rayto RT-6100)

Reproductive Studies: Human

**Amereh et al. 2022**		
**Domain**	**Rating**	**Justification for rating**
Are the study groups at risk of not representing their source populations in a manner that might introduce selection bias?	Probably low	There is insufficient information about participant selection to permit a judgment of low risk of bias, but there is indirect evidence which suggests that inclusion/exclusion criteria,<br><br>recruitment and enrollment procedures, and participation and follow-up rates were consistent across groups as described by the criteria for a judgment of low risk of bias
Was knowledge of the group assignments inadequately prevented (i.e., blinded or masked) during the study, potentially leading to subjective measurement of either exposure or outcome?	Probably High	No blinding or incomplete blinding, and the outcome measures or exposure measures is likely to be influenced by lack of blinding

Were exposure assessment methods lacking accuracy?	Low	There is high confidence in the accuracy of the exposure assessment methods, such as methods that have been tested for validity and reliability in measuring the targeted exposure and appropriate QA/QC for methods are described
Were outcome assessment methods lacking accuracy?	Apical (Weight): Low	Outcomes were assessed and defined consistently across all study participants, using valid and reliable measures
	Apical (Length): Low	Outcomes were assessed and defined consistently across all study participants, using valid and reliable measures
	Apical (Head circumference)" Low	Outcomes were assessed and defined consistently across all study participants, using valid and reliable measures
Was potential confounding inadequately incorporated?	High	Study did not control for any confounders
Were incomplete outcome data inadequately addressed?	Apical (Weight): Low	No missing outcome data
	Apical (Length): Low	No missing outcome data
	Apical (Head circumference): Low	No missing outcome data
Does the study report appear to have selective outcome reporting?	Probably Low	There is insufficient information about selective outcome reporting to permit a judgment of low risk of bias, but there is indirect evidence which suggests the study was free of selective reporting,
Conflict of Interest	Low	Funding source is limited to government, non-profit organizations, or academic grants funded by government, foundations and/or non-profit organizations without financial interest in the treatments studied
Other Potential Threats to Validity- Outcome Evaluation	Low	The study appears to be free of other sources of bias.

**Xue et al. 2024**

**Domain**	**Rating**	**Justification for rating**
Are the study groups at risk of not representing their source populations in a manner that might introduce selection bias?	Probably High	There is insufficient information about participant selection to permit a judgment of high risk of bias, but there is indirect evidence which suggests that inclusion/exclusion criteria, recruitment and enrollment procedures, and participation and follow-up rates were inconsistent across groups
Was knowledge of the group assignments inadequately prevented (i.e., blinded or masked) during the study, potentially leading to subjective measurement of either exposure or outcome?	Probably High	No blinding or incomplete blinding, and the outcome measures or exposure measures is likely to be influenced by lack of blinding
Were exposure assessment methods lacking accuracy?	Low	There is high confidence in the accuracy of the exposure assessment methods, such as methods that have been tested for validity and reliability in measuring the targeted exposure and appropriate QA/QC for methods are described
Were outcome assessment methods lacking accuracy?	Apical (Weight): Low	Outcomes were assessed and defined consistently across all study participants, using valid and reliable measures
	Other (Age): Low	Outcomes were assessed and defined consistently across all study participants, using valid and reliable measures
Was potential confounding inadequately incorporated?	Probably low	The study appropriately accounted for most but not all of the important confounders (Tier I)
Were incomplete outcome data inadequately addressed?	Apical (Weight): Low	No missing outcome data
	Other (Age): Low	No missing outcome data

Does the study report appear to have selective outcome reporting?	Probably Low	There is insufficient information about selective outcome reporting to permit a judgment of low risk of bias, but there is indirect evidence which suggests the study was free of selective reporting,
Conflict of Interest	Low	Funding source is limited to government, non-profit organizations, or academic grants funded by government, foundations and/or non-profit organizations without financial interest in the treatments studied
Other Potential Threats to Validity- Outcome Evaluation	Low	The study appears to be free of other sources of bias.

*Reproductive Studies: **Animal***

**An et al. 2021**		
**Domain**	**Rating**	**Justification for rating**
Sequence Generation	Probably low	Study authors make a simple statement such as 'we randomly allocated', but do not provide details regarding specific random component used in the sequence generation process.
Allocation Concealment	Probably high	Study authors do not make any statement about allocation concealment and the review author does not find indirect evidence suggesting allocation concealment.
Blinding of personnel and outcome assessors	Probably high	Study authors do not make any statement about blinding and the review author does not find indirect evidence suggesting blinding.
Incomplete Outcome Data	Apical: Number of growing follicles (ovaries): Probably low	Study authors report number of animals allocated to treatment groups, but do not provide data indicating adequate follow up for a subset of animals.
	Alters hormone receptor signaling; alters reproductive hormone production, secretion, or metabolism: AMH levels (ovaries): Probably low	Study authors report number of animals allocated to treatment groups, but do not provide data indicating adequate follow up for a subset of animals.

Selective Outcome Reporting	Probably low	All of the study's pre-specified outcomes outlined in the methods, that are of interest in the review have been reported in the pre-specified way (i.e., the outcomes outlined in the methods section match what is reported in the results section and vice versa), and the number of animals analyzed for outcomes of interest is provided.
Conflict of Interest	Low	The study did not receive support from a company, study author, or other party having a financial interest in the outcome of the study. A conflict of interest statement is provided to indicate the study authors have no financial interests and there is evidence of the parties not having a financial interest.
Other Potential Threats to Validity-Outcome Evaluation	Apical: Number of growing follicles (ovaries): Probably high	The number of growing follicles were observed by 5 visual fields under a microscope. Since it is a visual observation and no mention of blinding, there is evidence of potential high risk of bias.
	Alters hormone receptor signaling; alters reproductive hormone production, secretion, or metabolism: AMH levels (ovaries): Probably low	AMH levels were measured with ELISA kits. Because these are standard markers and they followed manufacturer's direction, these outcomes have no evidence of high risk of bias.

**J Hou et al. 2021**		
**Domain**	**Rating**	**Justification for rating**
Sequence Generation	Probably low	There is insufficient information about the sequence generation process to permit a judgment of low risk of bias, but study authors make a simple statement such as 'we randomly allocated', but do not provide details regarding specific random components used in the sequence generation process.
Allocation Concealment	Probably high	Study authors do not make any statement about allocation concealment and the review author does not find indirect evidence suggesting allocation concealment.
Blinding of personnel and outcome assessors	Probably high	Study authors do not make any statement about blinding and the review author does not find indirect evidence suggesting blinding.
Incomplete Outcome Data	Apical: Number of growing follicles (ovaries): Probably low	Study authors report number of animals allocated to treatment groups, but do not provide data indicating adequate follow up for all animals, only a subset of animals.

	Alters hormone receptor signaling; alters reproductive hormone production, secretion, or metabolism: AMH levels (pg/ml) IL-18 (pg/ml) IL-1β (pg/ml) (serum): Probably low	Study authors report number of animals allocated to treatment groups, but do not provide data indicating adequate follow up for all animals, only a subset of animals.
Selective Outcome Reporting	Probably low	All of the study's pre-specified outcomes outlined in the methods, that are of interest in the review have been reported in the pre-specified way (i.e., the outcomes outlined in the methods section match what is reported in the results section and vice versa), and the number of animals analyzed for outcomes of interest is provided.
Conflict of Interest	Low	The study did not receive support from a company, study author, or other party having a financial interest in the outcome of the study. A conflict of interest statement is provided to indicate the study authors have no financial interests and there is evidence of the parties not having a financial interest.
Other Potential Threats to Validity-Outcome Evaluation	Apical: Number of growing follicles (ovaries): Probably high	The number of growing follicles were observed by 5 visual fields under a microscope. Since it is a visual observation and no mention of blinding, there is evidence of potential high risk of bias.
	Alters hormone receptor signaling; alters reproductive hormone production, secretion, or metabolism: Hormone level changes: AMH levels (pg/ml) IL-18 (pg/ml) IL-1β (pg/ml) (serum): Probably low	AMH, IL-18 & IL-1β levels were measured with ELISA kits. Because these are standard markers and they followed manufacturer's direction, these outcomes have no evidence of high risk of bias.

**Huang et al. 2022**		
**Domain**	**Rating**	**Justification for rating**
Sequence Generation	Probably low	There is insufficient information about the sequence generation process to permit a judgment of low risk of bias, but there is indirect evidence that suggests the sequence generation process was random.

Allocation Concealment	Probably high	Study authors do not make any statement about allocation concealment and the review author does not find indirect evidence suggesting allocation concealment.
Blinding of personnel and outcome assessors	Probably high	Study authors do not make any statement about blinding and the review author does not find indirect evidence suggesting blinding.
Incomplete Outcome Data	Apical: Sperm count (epididymis): Probably low	The number of animals allocated is reported and matches the number of animals reported for each outcome (i.e., no missing outcome data). However, the originally, 32 pregnant mice were randomized into four groups (8 dams each), but only six dams and their male pups per group were followed during the experimental period.
Selective Outcome Reporting	Probably low	All of the study's pre-specified (primary and secondary) outcomes outlined in the methods, that are of interest in the review have been reported in the pre-specified way (i.e., the outcomes outlined in the methods section match what is reported in the results section and vice versa), and the number of animals analyzed for outcomes of interest is provided.
Conflict of Interest	Low	The study did not receive support from a company, study author, or other party having a financial interest in the outcome of the study. A conflict of interest statement is provided to indicate the study authors have no financial interests and there is evidence of the parties not having a financial interest.
Other Potential Threats to Validity-Outcome Evaluation	Apical: Sperm count (epididymis): Probably high	They used a hemocytometer to count the number of sperm according to manufacturer instructions. There was no blinding during this process. Only a brief description of the method. Therefore, there is some evidence of potential high risk of bias.

**B Hou et al. 2021**		
**Domain**	**Rating**	**Justification for rating**
Sequence Generation	Probably low	There is insufficient information about the sequence generation process to permit a judgment of low risk of bias, but study authors make a simple statement such as 'we randomly allocated', but do not provide details regarding specific random components used in the sequence generation process.
Allocation Concealment	Probably high	There is insufficient information about allocation concealment to permit a judgment of high risk of bias, but there is indirect evidence that suggests the allocation was not adequately concealed, as described by the criteria for a judgment of high risk of bias such as:

		Study authors do not make any statement about allocation concealment and the review author does not find indirect evidence suggesting allocation concealment.
Blinding of personnel and outcome assessors	Probably high	Study authors do not make any statement about blinding and the review author does not find indirect evidence suggesting blinding.
Incomplete Outcome Data	Apical: Rate of living sperm (Testis): Low	The number of animals allocated is reported and matches the number of animals reported for each outcome (i.e., no missing outcome data).
	Apical: Sperm malformation (Testis): Low	The number of animals allocated is reported and matches the number of animals reported for each outcome (i.e., no missing outcome data).
Selective Outcome Reporting	Probably low	All of the study's pre-specified outcomes outlined in the methods, that are of interest in the review have been reported in the pre-specified way (i.e., the outcomes outlined in the methods section match what is reported in the results section and vice versa), and the number of animals analyzed for outcomes of interest is provided.
Conflict of Interest	Low	The study did not receive support from a company, study author, or other party having a financial interest in the outcome of the study. A conflict of interest statement is provided to indicate the study authors have no financial interests and there is evidence of the parties not having a financial interest.
Other Potential Threats to Validity- Outcome Evaluation	Apical: Rate of living sperm (Testis): Probably high	A drop of sperm is dropped onto a slide and the living sperm was counted. One major risk of bias is the timing between preparing the sample and analyzing the sperm count. A longer processing time could lead to more sperm dying and then skewing the result. There was no mention of blinding which is important for this outcome.
	Apical: Sperm malformation (Testis): High	Sperm shape was analyzed by a microscope and observation. There is no mention how they got these results based on metrics. There are only a few descriptor words based on the shape. Therefore, this result has sufficient evidence of high risk of bias. There are example photographs but no explanation on how they get the result.

**Li et al. 2021**		
**Domain**	**Rating**	**Justification for rating**
Sequence Generation	Probably low	There is insufficient information about the sequence generation process to permit a judgment of low risk of bias, but study authors make a simple statement such as 'we

		randomly allocated', but do not provide details regarding specific random components used in the sequence generation process.
Allocation Concealment	Probably high	Study authors do not make any statement about allocation concealment and the review author does not find indirect evidence suggesting allocation concealment.
Blinding of personnel and outcome assessors	Probably high	Study authors do not make any statement about blinding and the review author does not find indirect evidence suggesting blinding.
Incomplete Outcome Data	Apical: Sperm abnormality (Testis): Low	The number of animals allocated is reported and matches the number of animals reported for each outcome.
	Apical: Sperm concentration (Testis): Low	The number of animals allocated is reported and matches the number of animals reported for each outcome.
	Apical: Sperm motility (Testis): Low	The number of animals allocated is reported and matches the number of animals reported for each outcome.
Selective Outcome Reporting	Probably low	All of the study's pre-specified outcomes outlined in the methods, that are of interest in the review have been reported in the pre-specified way (i.e., the outcomes outlined in the methods section match what is reported in the results section and vice versa), and the number of animals analyzed for outcomes of interest is provided.
Conflict of Interest	Low	The study did not receive support from a company, study author, or other party having a financial interest in the outcome of the study. A conflict of interest statement is provided to indicate the study authors have no financial interests and there is evidence of the parties not having a financial interest.
Other Potential Threats to Validity-Outcome Evaluation	Apical: Sperm motility (Testis): Probably low	The sperm abnormality was measured by an automatic sperm analyzer. There is no evidence of potential high risk of bias.
	Apical: Sperm concentration (Testis): Probably high	The sperm concentration was measured via microscope. The sperm suspension was smeared on a glass slide and five smears for each animal and 12 fields were randomly collected for observation. Blinding was never mentioned. Because the measurement was based on user observation, that could be high risk of bias.
	Apical: Sperm abnormality (Testis): Probably high	The sperm abnormality was measured via microscope. The sperm suspension was smeared on a glass slide and five smears for each animal and 12 fields were randomly collected for observation. Blinding was never mentioned. Because the measurement was based on user observation, that could be high risk of bias. They clearly explain the definition of sperm abnormality.

Jin et al. 2022		
**Domain**	**Rating**	**Justification for rating**
Sequence Generation	Probably low	There is insufficient information about the sequence generation process to permit a judgment of low risk of bias, but study authors make a simple statement such as 'we randomly allocated', but do not provide details regarding specific random components used in the sequence generation process.
Allocation Concealment	Probably high	Study authors do not make any statement about allocation concealment and the review author does not find indirect evidence suggesting allocation concealment.
Blinding of personnel and outcome assessors	Probably low	Study authors describe blinding for one experiment, and the methods for a second experiment are similar but do not specifically mention blinding.
Incomplete Outcome Data	Apical: Viability of sperm (Testis): Low	The number of animals allocated is reported and matches the number of animals reported for each outcome (i.e., no missing outcome data).
	Apical: Sperm deformity (Testis): Low	The number of animals allocated is reported and matches the number of animals reported for each outcome (i.e., no missing outcome data).
	Alters production and levels of reproductive hormones OR Alters hormone receptor levels/functions: Testosterone LH levels (ng/ml) FSH levels (ng/ml) Concentrations of testosterone (ng/ml) (serum): Probably low	Study authors report number of animals allocated to treatment groups, but do not provide data indicating adequate follow up for a subset of animals.
	Apical: Seminiferous tubular diameter, Germinal epithelium thickness (Testis): Probably low	Study authors report number of animals allocated to treatment groups, but do not provide data indicating adequate follow up for a subset of animals or only provide a qualitative statement about missing outcome data
Selective Outcome Reporting	Probably low	All of the study's pre-specified (primary and secondary) outcomes outlined in the protocol, methods, abstract, and/or introduction that are of interest in the review have been reported in the pre-specified way (i.e., the outcomes outlined in the methods section match what is

		reported in the results section and vice versa), and the number of animals analyzed for outcomes of interest is provided.
Conflict of Interest	Low	The study did not receive support from a company, study author, or other party having a financial interest in the outcome of the study. A conflict of interest statement is provided to indicate the study authors have no financial interests and there is evidence of the parties not having a financial interest.
Other Potential Threats to Validity-Outcome Evaluation	Apical: Viability of sperm (Testis): Probably low	They used a hemocytometer to count the number of sperm according to manufacturer instructions. The person was blinded and count to 1000 sperm. There is no evidence of potential high risk of bias
	Apical: Sperm deformity (Testis): Probably high	The person was blinded and count to 1000 sperm. Sperm deformity included specific shapes and morphologies manifested but still depends on user observation which has some bias. There is some evidence of potential high risk of bias
	Alters production and levels of reproductive hormones OR Alters hormone receptor levels/functions: Testosterone LH levels (ng/ml); FSH levels (ng/ml); Concentrations of testosterone (serum) (ng/ml): Probably Low	Testosterone, LH, and FSH were measured with ELISA kits. Because these are standard markers and they followed manufacturer's direction, these outcomes have no evidence of high risk of bias.
	Apical: Testicular Damage: Seminiferous tubular diameter, Germinal epithelium thickness (Testis): Probably high	Sufficient number of slides for statistical analysis, used image-based software for measurement, no mention of blinding, 'round' or 'nearly round' does not have a clear definition so could have potentially high selection bias.

**Saeed, et al. 2022**		
**Domain**	**Rating**	**Justification for rating**
Sequence Generation	Probably high	Study authors do not make any statement about sequence generation and the review author does not find indirect evidence suggesting random sequence generation.

Allocation Concealment	Probably high	Study authors do not make any statement about allocation concealment and the review author does not find indirect evidence suggesting allocation concealment.
Blinding of personnel and outcome assessors	High	Study authors do not make any statement about allocation concealment and the review author does not find indirect evidence suggesting allocation concealment.
Incomplete Outcome Data	Alters production and levels of reproductive hormones OR Alters hormone receptor levels/functions: (FSH, Estradiol, LH, Progesterone, Tesosterone): Low	The number of animals allocated is reported and matches the number of animals reported for each outcome (i.e., no missing outcome data)
Selective Outcome Reporting	Probably low	All of the study's pre-specified outcomes outlined in the methods, abstract, that are of interest in the review have been reported in the pre-specified way, but study authors only report a subset of animals were examined for outcome of interest.
Conflict of Interest	Probably low	There is insufficient information to permit a judgment of low risk of bias, but there is indirect evidence that suggests the study is free of conflicts of interest, as described by the criteria for a judgment of low risk of bias<br>No funding statement provided. Authors are form academic institutions and they have no COI
Other Potential Threats to Validity-Outcome Evaluation	Hormone level changes (FSH, Estradiol, LH, Progesterone, Tesosterone): High	The concentrations of estradiol, LH, FSH, progesterone, and testosterone in the blood samples were analyzed. Methods inadequately described. Concerns of a high risk of bias.

**Zhao et al., 2024**		
**Domain**	**Rating**	**Justification for rating**
Sequence Generation	Probably low	Study authors make a simple statement such as 'we randomly allocated', but do not provide details regarding specific random component used in the sequence generation process.

Allocation Concealment	Probably high	Study authors do not make any statement about allocation concealment and the review author does not find indirect evidence suggesting allocation concealment.
Blinding of personnel and outcome assessors	Probably low	Study authors state that some study personnel were blinded, but it is unclear if all important personnel were blinded and it is unlikely that the blinding could have been broken;
Incomplete Outcome Data	Apical: AGI/AGD: Probably low	Study authors report number of animals allocated to treatment groups, and provide data indicating adequate follow up for only a subset of animals that is equal across each treatment group.
	Other: Age at puberty: Probably low	Study authors report number of animals allocated to treatment groups, and provide data indicating adequate follow up for only a subset of animals that is equal across each treatment group.
	Apical: Sperm counts & motility: Probably low	Study authors report number of animals allocated to treatment groups, and provide data indicating adequate follow up for only a subset of animals that is equal across each treatment group.
	Alters production and levels of reproductive hormones OR Alters hormone receptor levels/functions: Testosterone, Luteinizing hormones, Follicle stimulating hormone, Inhibin: Probably low	Study authors report number of animals allocated to treatment groups, and provide data indicating adequate follow up for only a subset of animals that is equal across each treatment group.
Selective Outcome Reporting	Probably low	All of the study's pre-specified outcomes outlined in the methods, abstract, that are of interest in the review have been reported in the pre-specified way, but study authors only report a subset of animals were examined for outcome of interest
Conflict of Interest	Low	Funding source is limited to government, non-profit organizations, or academic grants funded by government, foundations and/or non-profit organizations without financial interest in the treatments studied
Other Potential Threats to Validity-	Apical: AGI/AGD: Probably low	First, we observed and recorded the weight of the body and testes and calculated the anogenital index (AGI). Measured in a standard way. There is no evidence of potential high risk of bias.

Outcome Evaluation	Other: Age at puberty: Probably Low	The separation of the prepuce from the glans penis occurs around the time of puberty (Korenbrot et al., 1977). Balanopreputial separation is an indicator of activation of the reproductive axis in males. It was assessed from PND 22 in each group.
	Apical: Sperm counts & motility: Probably low	At least six fields were assessed for each sample, and the sperm concentration and percentages of motile spermatozoa were determined. Measured in a standard way. There is no evidence of potential high risk of bias.
	Alters production and levels of reproductive hormones OR Alters hormone receptor levels/functions: Testosterone, Luteinizing hormones, Follicle stimulating hormone, Inhibin: Probably low	Ten samples from each group were randomly selected for testing, and each assay was conducted with three technical replicates. Measured in a standard way. There is no evidence of potential high risk of bias.

Zhang et al. 2023		
**Domain**	**Rating**	**Justification for rating**
Sequence Generation	Probably low	Study authors make a simple statement such as 'we randomly allocated', but do not provide details regarding specific random component used in the sequence generation process.
Allocation Concealment	Probably high	Study authors do not make any statement about allocation concealment and the review author does not find indirect evidence suggesting allocation concealment.
Blinding of personnel and outcome assessors	Probably high	Study authors do not make any statement about blinding and the review author does not find indirect evidence suggesting blinding.
Incomplete Outcome Data	Alters production and levels of reproductive hormones OR Alters hormone receptor levels/functions (E2, P, FSH and LH): High	Review author is not confident that the animals included in the analysis are exactly those who were randomized into the experiment. The number of animals allocated to treatment groups is not reported for outcomes of interest data are not provided to indicate that there was adequate follow up of all animals from the beginning of the experiment
	Apical (Oocyte meiotic progression): High	Review author is not confident that the animals included in the analysis are exactly those who were randomized into the experiment. The number of animals allocated to treatment groups is not reported for outcomes of interest data are not provided to indicate that there was adequate follow up of all animals from the beginning of the experiment
	Apical (Blatstocyst development): High	Review author is not confident that the animals included in the analysis are exactly those who were randomized into the experiment. The number of animals allocated to treatment groups is not reported for outcomes of interest data are not provided to indicate that there was adequate follow up of all animals from the beginning of the experiment
	Other (Litter size): High	Review author is not confident that the animals included in the analysis are exactly those who were randomized into the experiment. The number of animals allocated to treatment groups is not reported for outcomes of interest data are not provided to indicate that there was adequate follow up of all animals from the beginning of the experiment

Selective Outcome Reporting	Probably Low	All of the study's pre-specified outcomes outlined in the methods, abstract, that are of interest in the review have been reported in the pre-specified way, but study authors only report a subset of animals were examined for outcome of interest
Conflict of Interest	Low	Funding source is limited to government, non-profit organizations, or academic grants funded by government, foundations and/or non-profit organizations without financial interest in the treatments studied
Other Potential Threats to Validity- Outcome Evaluation	Alters production and levels of reproductive hormones OR Alters hormone receptor levels/functions (E2, P, FSH and LH): Probably High	No description of assessment methods
	Apical (Oocyte meiotic progression): Probably low	Measured in a standard way. There is no evidence of potential high risk of bias.
	Apical (Blatstocyst development): Probably low	Measured in a standard way. There is no evidence of potential high risk of bias.
	Other (Litter size): Probably low	Measured in a standard way. There is no evidence of potential high risk of bias.

**Agahei et al. 2022**		
**Domain**	**Rating**	**Justification for rating**
Sequence Generation	Probably low	Study authors make a simple statement such as 'we randomly allocated', but do not provide details regarding specific random component used in the sequence generation process.
Allocation Concealment	Probably high	Study authors do not make any statement about allocation concealment and the review author does not find indirect evidence suggesting allocation concealment.
Blinding of personnel and outcome assessors	Probably high	Study authors do not make any statement about blinding and the review author does not find indirect evidence suggesting blinding.
Incomplete Outcome Data	Probably low	Study authors report number of animals allocated to treatment groups, and provide data indicating adequate follow up for only a subset of animals that is equal across each treatment group.

Selective Outcome Reporting	Probably low	All of the study's pre-specified outcomes outlined in the methods, abstract, that are of interest in the review have been reported in the pre-specified way, but study authors only report a subset of animals were examined for outcome of interest
Conflict of Interest	Low	Funding source is limited to government, non-profit organizations, or academic grants funded by government, foundations and/or non-profit organizations without financial interest in the treatments studied
Other Potential Threats to Validity-Outcome Evaluation	Low	There is high confidence in the accuracy of the outcome assessment methods, such as methods that have been tested for validity and reliability in measuring the targeted outcome.

**Wu et al. 2023**		
**Domain**	**Rating**	**Justification for rating**
Sequence Generation	Probably low	Study authors make a simple statement such as 'we randomly allocated', but do not provide details regarding specific random component used in the sequence generation process.
Allocation Concealment	Probably high	Study authors do not make any statement about allocation concealment and the review author does not find indirect evidence suggesting allocation concealment.
Blinding of personnel and outcome assessors	Probably high	Study authors do not make any statement about blinding and the review author does not find indirect evidence suggesting blinding.
Incomplete Outcome Data	Probably low	Study authors report number of animals allocated to treatment groups, but do not provide data indicating adequate follow up for a subset of animals
	Probably low	Study authors report number of animals allocated to treatment groups, but do not provide data indicating adequate follow up for a subset of animals
	Probably low	Study authors report number of animals allocated to treatment groups, but do not provide data indicating adequate follow up for a subset of animals
Selective Outcome Reporting	Probably low	All of the study's pre-specified outcomes outlined in the methods, abstract, that are of interest in the review have been reported in the pre-specified way, but study authors only report a subset of animals were examined for outcome of interest

Conflict of Interest	Low	Funding source is limited to government, non-profit organizations, or academic grants funded by government, foundations and/or non-profit organizations without financial interest in the treatments studied
Other Potential Threats to Validity- Outcome Evaluation	Low	There is high confidence in the accuracy of the outcome assessment methods, such as methods that have been tested for validity and reliability in measuring the targeted outcome.

Respiratory Studies: Human

**Tas et al. 2024**		
**Domain**	**Rating**	**Justification for rating**
Are the study groups at risk of not representing their source populations in a manner that might introduce selection bias?	Probably High	There is insufficient information about participant selection to permit a judgment of high risk of bias, but there is indirect evidence which suggests that inclusion/exclusion criteria, recruitment and enrollment procedures, and participation and follow-up rates were inconsistent across groups
Was knowledge of the group assignments inadequately prevented (i.e., blinded or masked) during the study, potentially leading to subjective measurement of either exposure or outcome?	Probably Low	Diagnoses was made on enrollment into the study
Were exposure assessment methods lacking accuracy?	Probably High	There is insufficient information about the exposure assessment methods to permit a judgment of high risk of bias, but there is indirect evidence which suggests that methods were not robust
Were outcome assessment methods lacking accuracy?	Low	Outcomes were assessed and defined consistently across all study participants, using valid and reliable measures

Was potential confounding inadequately incorporated?	High	Important potential confounders were not measured
Were incomplete outcome data inadequately addressed?	Low	No missing outcome data
Does the study report appear to have selective outcome reporting?	Probably Low	There is insufficient information about selective outcome reporting to permit a judgment of low risk of bias, but there is indirect evidence which suggests the study was free of selective reporting,
Conflict of Interest	Probably Low	There is insufficient information to permit a judgment of low risk of bias, but there is indirect evidence which suggests the study was free of support from a company, study author, or other entity having a financial interest in the outcome of the study
Other Potential Threats to Validity- Outcome Evaluation	Low	The study appears to be free of other sources of bias.

*Respiratory Studies: **Animal***

**Y Li et al. 2022**		
**Domain**	**Rating**	**Justification for rating**
Sequence Generation	Probably low	Study authors make a simple statement such as 'we randomly allocated', but do not provide details regarding specific random component used in the sequence generation process.
Allocation Concealment	Probably high	Study authors do not make any statement about allocation concealment and the review author does not find indirect evidence suggesting allocation concealment.
Blinding of personnel and outcome assessors	Lung injury: Lung tissue score, pulmonary parenchymal area, average vessel	Investigators report blinding of key study personnel.

	thickness, number of alveolar septal: Low	
	Induces chronic inflammation: Lung collagen area: Low	Investigators report blinding of key study personnel.
	Pulmonary function: tissue damping, tissue elastance, central airway resistance, peak expiratory flow, forced vital capacity, forced expiratory volume, forced expiratory volume at 1s, FEV0.1/ FV: Probably high	Study authors do not make any statement about blinding and the review author does not find indirect evidence suggesting blinding.
	Cell count: Number of total cells, macrophages, lymphocytes, neutrophils in bronchoalveolar lavage fluid: Probably high	Study authors do not make any statement about blinding and the review author does not find indirect evidence suggesting blinding. There is no indication of whether or not cells were counted by a machine.
Incomplete Outcome Data	Probably low	Study authors report number of animals allocated to treatment groups, and provide data indicating adequate follow up for only a subset of animals that is equal across each treatment group.
Selective Outcome Reporting	Probably Low	All of the study's pre-specified outcomes outlined in the protocol, methods, abstract, and/or introduction that are of interest in the review have been reported in the pre-specified way.

Conflict of Interest	Low	The study did not receive support from a company, study author, or other party having a financial interest in the outcome of the study. A conflict of interest statement is provided to indicate the study authors have no financial interests and there is evidence of the parties not having a financial interest.
Other Potential Threats to Validity – Outcome Evaluation	Lung injury: pulmonary parenchymal area, average vessel thickness, number of alveolar septal: Probably low	Measured using Image J software to decrease interobserver variability. Measured in a standard way. There is no evidence of potential high risk of bias.
	Lung injury: Lung tissue score: High	Study authors used the Ashcroft score to measure the amount of fibrosis. Authors used a total of 20 fields per exposure level (4 mice x 5 fields). The primary literature of the Ashcoft score notes that there are issues with interobserver variability especially at lower number of fields of 20. Therefore, risk of bias is high considering the small sample size (Ashcroft T, Simpson JM, Timbrell V. Simple method of estimating severity of pulmonary fibrosis on a numerical scale. J Clin Pathol. 1988 Apr;41(4):467-70. doi: 10.1136/jcp.41.4.467. PMID: 3366935; PMCID: PMC1141479.)
	Induces chronic inflammation: Lung collagen area: High	Interobserver variability of Sirius staining is high per literature review.
	Pulmonary function: tissue damping, tissue elastance, central airway resistance, peak expiratory flow, forced vital capacity, forced expiratory volume, forced expiratory volume at	Standard measurement was used with anesthetized animals. There is no evidence of potential high risk of bias.

	1s, FEV0.1/ FV: Probably low	
	Cell count: Number of total cells, macrophages, lymphocytes, neutrophils in bronchoalveolar lavage fluid: Probably low	Cell classification count of BALF was performed by counting 400 cells per slide, including macrophages, neutrophils, and lymphocytes. This is a standard technique for BALF.

**Fan et al., 2022**		
**Domain**	**Rating**	**Justification for rating**
Sequence Generation	Probably low	Study authors make a simple statement such as 'we randomly allocated', but do not provide details regarding specific random component used in the sequence generation process.
Allocation Concealment	Probably high	There is insufficient information about allocation concealment to permit a judgment of high risk of bias, but there is indirect evidence that suggests the allocation was not adequately concealed, as described by the criteria for a judgment of high risk of bias. Study authors do not make any statement about allocation concealment and the review author does not find indirect evidence suggesting allocation concealment.
Blinding of personnel and outcome assessors	Probably high	Study authors do not make any statement about blinding and the review author does not find indirect evidence suggesting blinding.
Incomplete Outcome Data	Low	The number of animals allocated to treatment groups is reported for outcomes of interest and data are provided indicating adequate follow up of all animals from the beginning of the study.

Selective Outcome Reporting	Probably low	There was no protocol, and the outcomes were only described in the methods, abstract, and/or introduction; There is insufficient information about selective outcome reporting to permit a judgment of low risk of bias, but there is indirect evidence that suggests the study was free of selective reporting.
Conflict of Interest	Low	The study did not receive support from a company, study author, or other party having a financial interest in the outcome of the study. An author conflict of interest statement is provided to indicate the study authors have no financial interests and there is evidence of the parties not having a financial interest.
Other Potential Threats to Validity-Outcome evaluation	Probably low	Measured in a standard way in BALF. There is no evidence of potential high risk of bias.

**X. Li et al., 2022**		
**Domain**	**Rating**	**Justification for rating**
Sequence Generation	Probably high	Study authors do not make any statement about sequence generation and the review author does not find indirect evidence suggesting random sequence generation.
Allocation Concealment	Probably high	Study authors do not make any statement about allocation concealment and the review author does not find indirect evidence suggesting allocation concealment.
Blinding of personnel and outcome assessors	Probably high	Study authors do not make any statement about blinding and the review author does not find indirect evidence suggesting blinding.
Incomplete Outcome Data	Probably low	Study authors report number of animals allocated to treatment groups, but do not provide data indicating adequate follow up for a subset of animals.
Selective Outcome Reporting	Probably low	There was no protocol, and the outcomes were only described in the methods, abstract, and/or introduction; There is insufficient information about selective outcome reporting to

		permit a judgment of low risk of bias, but there is indirect evidence that suggests the study was free of selective reporting.
Conflict of Interest	Low	The study did not receive support from a company, study author, or other party having a financial interest in the outcome of the study. An author conflict of interest statement is provided to indicate the study authors have no financial interests and there is evidence of the parties not having a financial interest.
Other Potential Threats to Validity- Outcome evaluation	Probably low	Measured in a standard way.

**Lim et al., 2021**		
**Domain**	**Rating**	**Justification for rating**
Sequence Generation	Probably low	OECD guidelines indicate randomization should occur, but do not specify how. Study authors indicate they followed guidelines but do not specify how randomization occurred.
Allocation Concealment	Probably low	OECD guidelines state that animals should be given identification numbers, but authors do not provide details.
Blinding of personnel and outcome assessors	Probably high	Study authors do not make any statement about blinding and the review author does not find indirect evidence suggesting blinding.
Incomplete Outcome Data	Cell count: total cell count, macrophage, polymorphonuclear, and lymphocytes in bronchoalveolar lavage fluid; Induces chronic inflammation: Lung	Study authors report number of animals allocated to treatment groups, and provide data indicating adequate follow up for only a subset of animals that is equal across each treatment group.

	inflammatory index: Probably low	
	Pulmonary function: frequency, tidal volume, minute volume, inspiratory time, expiratory time, peak inspiratory flow, peak expiratory flow: Low	The number of animals allocated is reported and matches the number of animals reported for each outcome (i.e., no missing outcome data).
Selective Outcome Reporting	Probably low	Not all of the study's pre-specified primary outcomes (as outlined in the protocol, i.e. OECD TG 412) that are of interest in the review have been reported in the pre-specified way, but study authors reported all of the data for the pre-specified outcomes that were listed in the abstract and methods section of the paper.
Conflict of Interest	Low	The study did not receive support from a company, study author, or other party having a financial interest in the outcome of the study. An author conflict of interest statement is provided to indicate the study authors have no financial interests and there is evidence of the parties not having a financial interest.
Other Potential Threats to Validity-Outcome evaluation	Cell count: total cell count, macrophage, polymorphonuclear, and lymphocytes in bronchoalveolar lavage fluid; Induces oxidative stress: total protein, albumin, lactate dehydrogenase in bronchoalveolar lavage fluid; Induces chronic inflammation: Lung inflammatory index: Probably low	Measured in a standard way in BALF. There is no evidence of potential high risk of bias.

	Pulmonary function: frequency, tidal volume, minute volume, inspiratory time, expiratory time, peak inspiratory flow, peak expiratory flow: High	Plethysmography in an awake and moving animal is highly variable. It was performed using whole body plethysmography. Literature review indicated low sensitivity and specificity of the oxidative stress and inflammatory markers.

**Wu et al. 2022**		
**Domain**	**Rating**	**Justification for rating**
Sequence Generation	Probably low	Study authors make a simple statement such as 'we randomly allocated', but do not provide details regarding specific random component used in the sequence generation process.
Allocation Concealment	Probably high	Study authors do not make any statement about allocation concealment and the review author does not find indirect evidence suggesting allocation concealment.
Blinding of personnel and outcome assessors	Probably high	Study authors do not make any statement about blinding and the review author does not find indirect evidence suggesting blinding.
Incomplete Outcome Data	Low	No missing outcome data
Selective Outcome Reporting	Probably Low	All of the study's pre-specified outcomes outlined in the methods, abstract, that are of interest in the review have been reported in the pre-specified way, but study authors only report a subset of animals were examined for outcome of interest
Conflict of Interest	Low	Funding source is limited to government, non-profit organizations, or academic grants funded by government, foundations and/or non-profit organizations without financial interest in the treatments studied
Other Potential Threats to Validity- Outcome Evaluation	Probably low	Measured in a standard way. There is no evidence of potential high risk of bias.

Woo et al. 2023		
**Domain**	**Rating**	**Justification for rating**
Sequence Generation	Probably High	Study authors do not make any statement about sequence generation and the review author does not find indirect evidence suggesting random sequence generation
Allocation Concealment	Probably high	Study authors do not make any statement about allocation concealment and the review author does not find indirect evidence suggesting allocation concealment.
Blinding of personnel and outcome assessors	Probably high	Study authors do not make any statement about blinding and the review author does not find indirect evidence suggesting blinding.
Incomplete Outcome Data	Apical: Lung Injury (Alveolar epithelial hyperplasia): High	Data are not provided to indicate that there was adequate follow up of all animals from the beginning of the experiment.
	Apical: Cell Count (Macrophage aggregates, Macrophage cell counts, Cell count (total), Cell count (lymphoctyes), Cell count (neutrophils), Inflammatory cell infiltration): High	Data are not provided to indicate that there was adequate follow up of all animals from the beginning of the experiment
	Induces oxidative stress: (ROS): High	Data are not provided to indicate that there was adequate follow up of all animals from the beginning of the experiment
	Induce chronic inflammation: (TNF-α Secretions, IL-1β, Secretions, IL-6, MCP-1, KC: High	Data are not provided to indicate that there was adequate follow up of all animals from the beginning of the experiment
Selective Outcome Reporting	Probably Low	All of the study's pre-specified outcomes outlined in the methods, abstract, that are of interest in the review have been reported in the pre-specified way, but study authors only report a subset of animals were examined for outcome of interest
Conflict of Interest	Probably Low	There is insufficient information to permit a judgment of low risk of bias, but there is indirect evidence that suggests the study is free of conflicts of interest,

Other Potential Threats to Validity- Outcome Evaluation	Apical: Lung Injury (Alveolar epithelial hyperplasia): Probably low	Histopathological analysis Te left lung of each mice was fixed with 10% neutral bufered formalin (NRF). Te specimens were dehydrated and embedded in parafn to produce tissue blocks which were sectioned into 4-µm thick slices. Lung sections from each animal were stained with haematoxylin and eosin (H&E). All samples were analysed using a Leica DM2500 microscope (Leica Instruments, Wetzlar, Germany) at 200×and 400×magnifcations. Te degree of lung injury in each animal was scored on a scale as follow: 0, no symptoms; 1, minimal; 2, slight; 3, moderate; 4, severe.
	Apical: Cell Count (Macrophage aggregates, Macrophage cell counts, Cell count (total), Cell count (lymphoctyes), Cell count (neutrophils), Inflammatory cell infiltration): Probably low	Measured in a standard way in BALF. There is no evidence of potential high risk of bias.
	Induces oxidative stress: (ROS): Probably low	Measured in a standard way. There is no evidence of potential high risk of bias.
	Induce chronic inflammation: (TNF-α Secretions, IL-1β, Secretions, IL-6, MCP-1, KC: Probably low	Te TNF-α, IL-1β, IL-6, MCP-1, and KC levels in BALF were quantified by ELISA using a commercial kit (R&D Systems) in accordance with the manufacturer's protocols. Measured in a standard way in BALF. There is no evidence of potential high risk of bias.

**Yang et al. 2024**		
**Domain**	**Rating**	**Justification for rating**
Sequence Generation	Probably low	Study authors make a simple statement such as 'we randomly allocated', but do not provide details regarding specific random component used in the sequence generation process.
Allocation Concealment	Probably high	Study authors do not make any statement about allocation concealment and the review author does not find indirect evidence suggesting allocation concealment.
Blinding of personnel and outcome assessors	Probably high	Study authors do not make any statement about blinding and the review author does not find indirect evidence suggesting blinding.

Incomplete Outcome Data	Apical: Pulmonary function: Probably low	Study authors report number of animals allocated to treatment groups, but do not provide data indicating adequate follow up for a subset of animals
	Apical Lung Injury (Mean Chord Length, Thickness of Fibrosis Layer: Probably low	Study authors report number of animals allocated to treatment groups, but do not provide data indicating adequate follow up for a subset of animals
	Apical: Cell counts (Cell count (macrophages), Cell count (total cells), Cell count (lymphoctyes), Cell count (neutrophils): Probably low	Study authors report number of animals allocated to treatment groups, but do not provide data indicating adequate follow up for a subset of animals
	Induces chronic inflammation: TNF-α, IL-6, MCP-1: Probably low	Study authors report number of animals allocated to treatment groups, but do not provide data indicating adequate follow up for a subset of animals
	Induces oxidative stress: ROS, SOD, GSH: Probably low	Study authors report number of animals allocated to treatment groups, but do not provide data indicating adequate follow up for a subset of animals
Selective Outcome Reporting	Probably Low	All of the study's pre-specified outcomes outlined in the methods, abstract, that are of interest in the review have been reported in the pre-specified way, but study authors only report a subset of animals were examined for outcome of interest
Conflict of Interest	Low	Funding source is limited to government, non-profit organizations, or academic grants funded by government, foundations and/or non-profit organizations without financial interest in the treatments studied
Other Potential Threats to Validity-Outcome Evaluation	Apical (pulmonary function): Probably low	For respiratory function, frequency (F), and indicators of bronchoconstriction, including tidal mid-expiratory flow (EF50) and enhanced pause (Penh) were measured in real time using the plethysmograph (Buxco® FinePointe, USA).
	Apical Lung Injury (Mean Chord Length, Thickness of Fibrosis Layer: Probably low	The left lung samples were fixed in 10% neutral formalin buffer for 24 h at 4◦C. Then, fixed tissues were embedded in paraffin, serially sectioned into 5 μm thick slices, and stained with hematoxylin and eosin (H&E) or Masson dye. Microscopic observation was performed to examine the lung histopathological changes (IX83, Olympus, Japan). Established indices of experimental COPD, mean chord lengths (Lm) and thickness of fibrosis layer around airway, were measured following the literature (Laucho-Contreras et al., 2015; Wang et al., 2023).
	Apical: Cell counts (Cell count (macrophages), Cell count	The left lung was ligated, BALFs were collected from anesthetized mice by injecting 1 mL of saline into the right lung serval times lavage. Then, collected BALFs were centrifuged at

	(total cells), Cell count (lymphoctyes), Cell count (neutrophils): Probably low	500 g for 5 min at 4 ◦C. The supernatants were transferred to analyze ROS, ALB, TP, LDH, and inflammatory cytokines using ELISA kits. The cell deposition was resuspended to count cell number, including total cell number, macrophages, neutrophils, and lymphocytes by flow cytometry.
	Induces chronic inflammation: TNF-α, IL-6, MCP-1: Probably low	Measured in a standard way. There is no evidence of potential high risk of bias.
	Induces oxidative stress: ROS, SOD, GSH: Probably low	Measured in a standard way. There is no evidence of potential high risk of bias.
