# Supplementary material for: Effects of Microplastic Exposure on Human Digestive, Reproductive, and Respiratory Health: A Rapid Systematic Review
Source: Environ Sci Technol. 2024 Dec 18;58(52):22843–64. doi: 10.1021/acs.est.3c09524 (PMC11697325; doi:10.1021/acs.est.3c09524)
Supplement: Supplementary file 9 — es3c09524_si_009.pdf [file es3c09524_si_009.pdf]

Supplementary File 9, Graphical Display of Results

Digestive Results

Figure S1. Alterations of cell proliferation, cell death, or nutrient supply

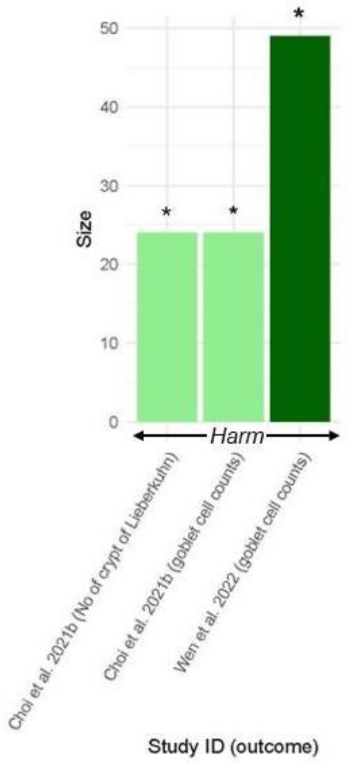

Key:
Direction of effect: All included study results show change in the direction of 'harm'
Y axis = sample size
Dark green = P<0.001, Green = P<0.01, Light green = ≤ 0.05, Red = > 0.05
*= Dose response identified in the study

Figure S2. Induction of chronic inflammation

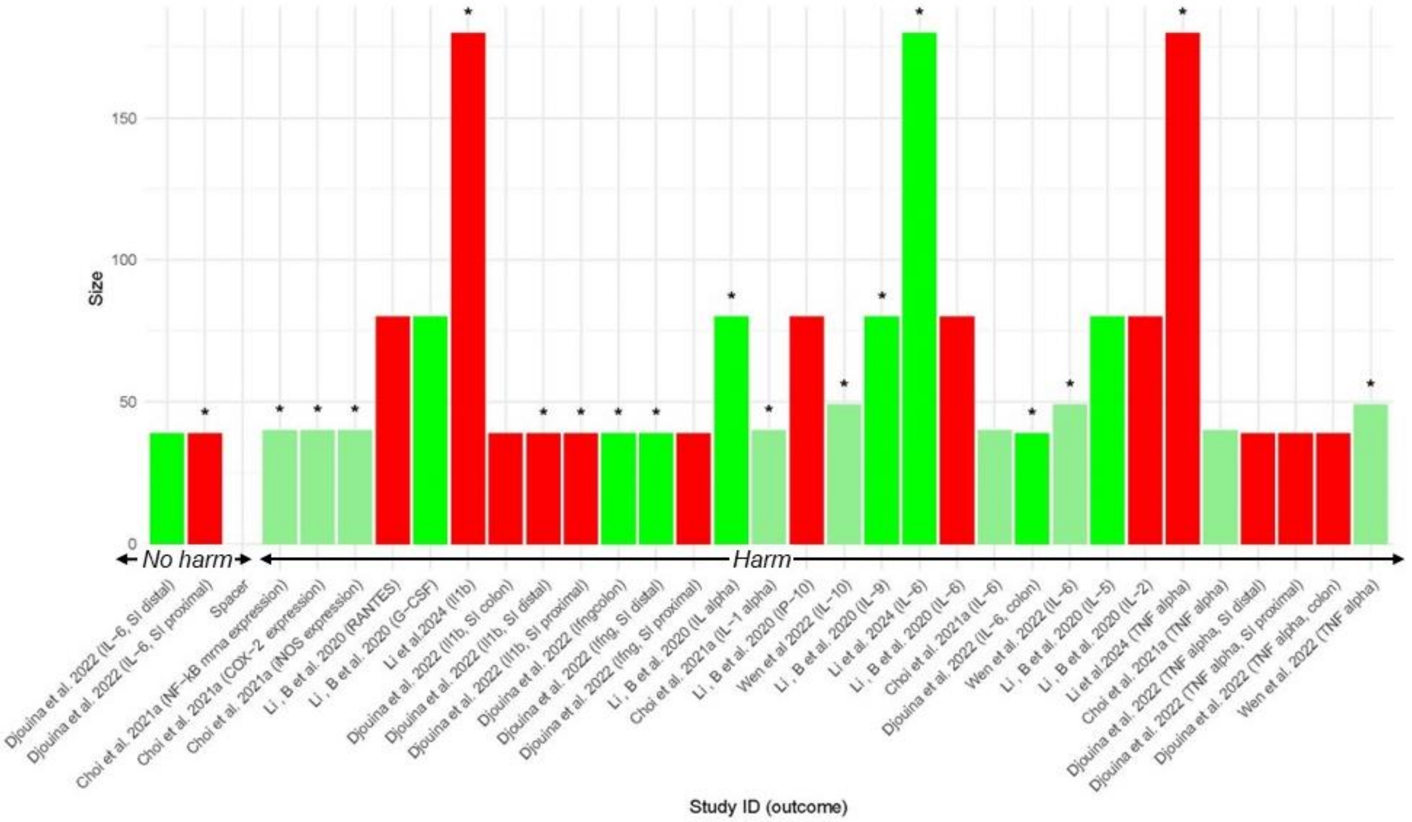

Key:
Direction of effect: Two study results show ‘no harm’, and 30 study results show change in the direction of ‘harm’
Y axis = sample size
Dark green = P<0.001, Green = P<0.01, Light green = ≤ 0.05, Red = > 0.05
*= Dose response identified in the study

Figure S3. Immunosuppressive effects

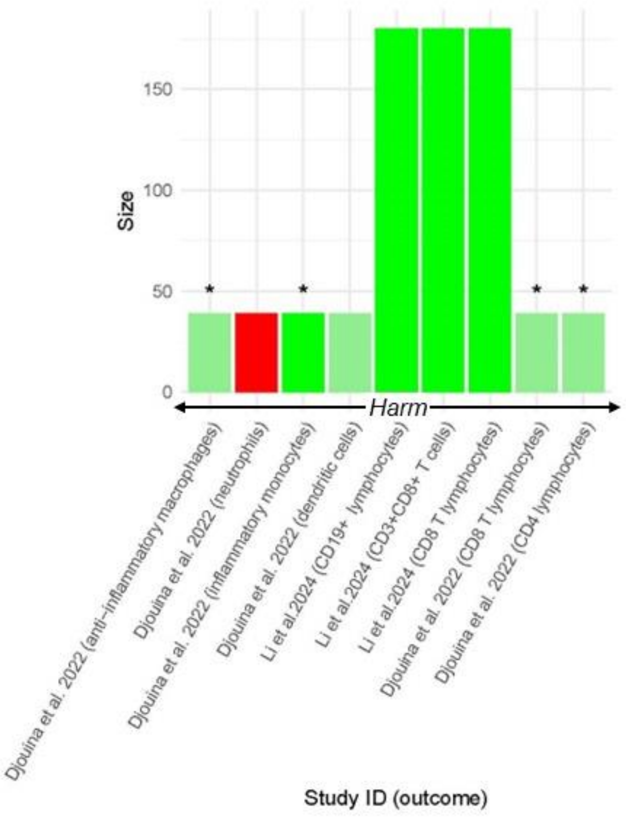

Key:
Direction of effect: All included study results show change in the direction of 'harm'
Y axis = sample size
Dark green = P<0.001, Green = P<0.01, Light green = ≤ 0.05, Red = > 0.05
*= Dose response identified in the study

Reproductive Results

Figure S4. Sperm quality

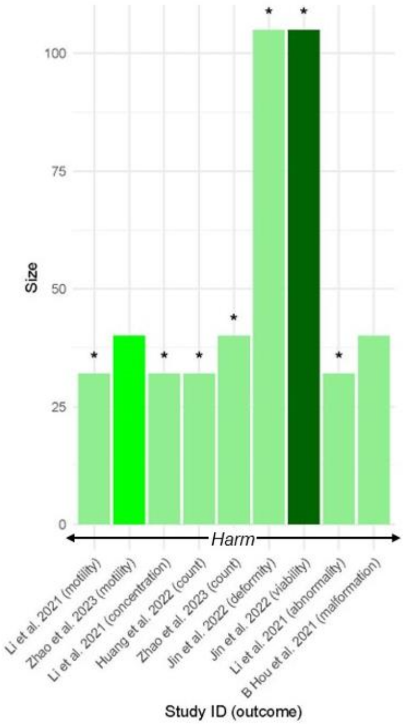

Key:
Direction of effect: All included study results show change in the direction of 'harm'
Y axis = sample size
Dark green = P<0.001, Green = P<0.01, Light green = ≤ 0.05, Red = > 0.05
*= Dose response identified in the study

Figure S5. Follicles/Ovarian reserve capacity

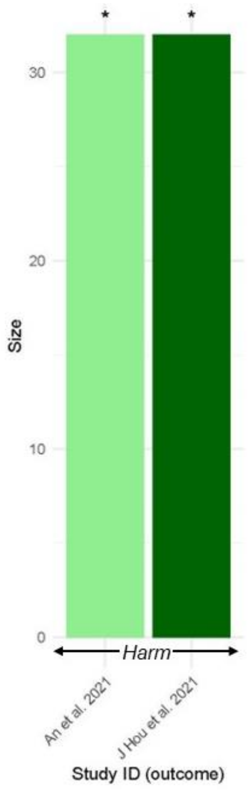

Key:
Direction of effect: All included study results show change in the direction of ‘harm’
Y axis = sample size
Dark green = P<0.001, Green = P<0.01, Light green = ≤ 0.05, Red = > 0.05
*= Dose response identified in the study

Figure S6. Reproductive hormones

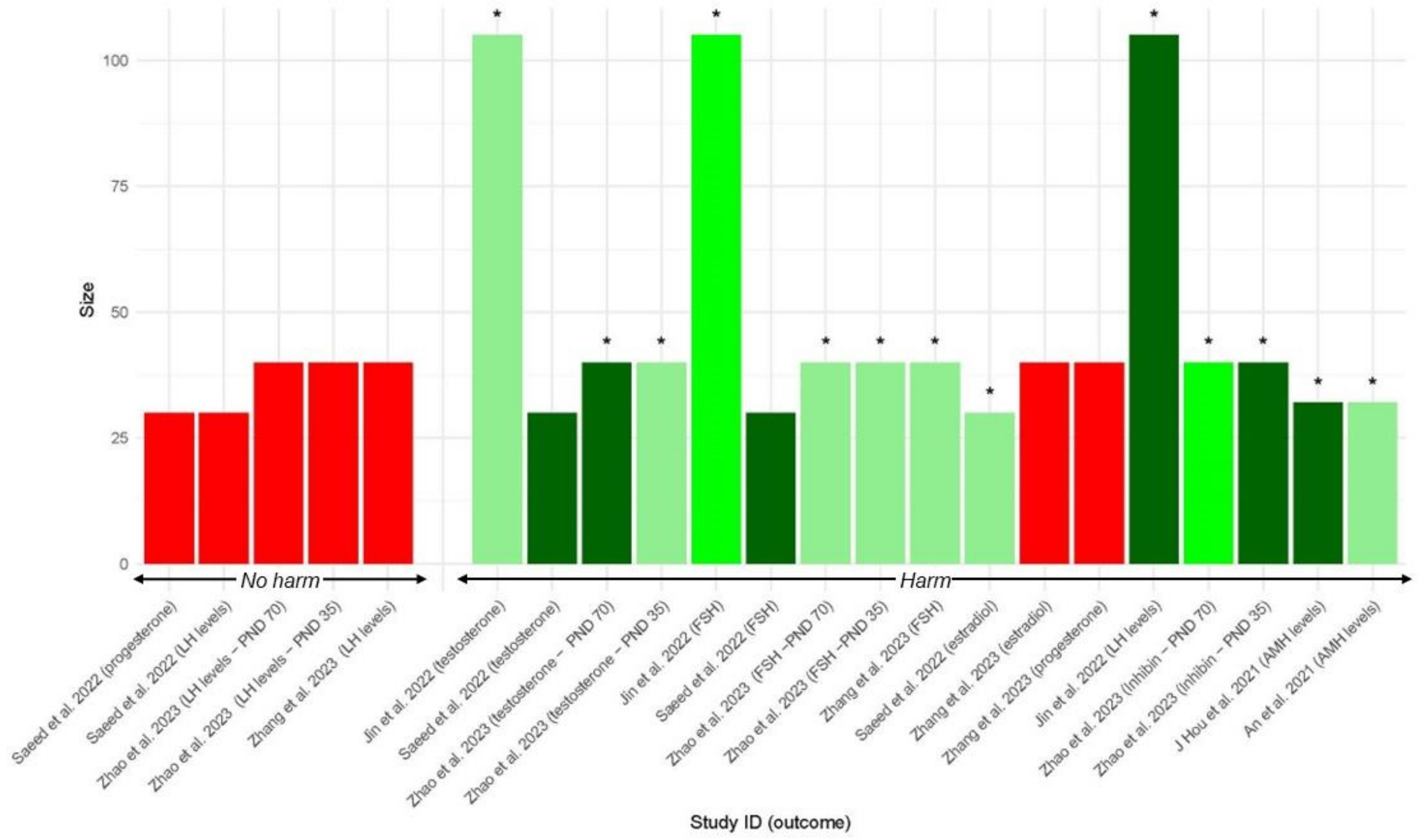

Key:
Direction of effect: Five study results show 'no harm', and 17 study results show change in the direction of 'harm'
Y axis = sample size
Dark green = P<0.001, Green = P<0.01, Light green = ≤ 0.05, Red = > 0.05
*= Dose response identified in the study

Respiratory Results

Figure S7. Pulmonary function

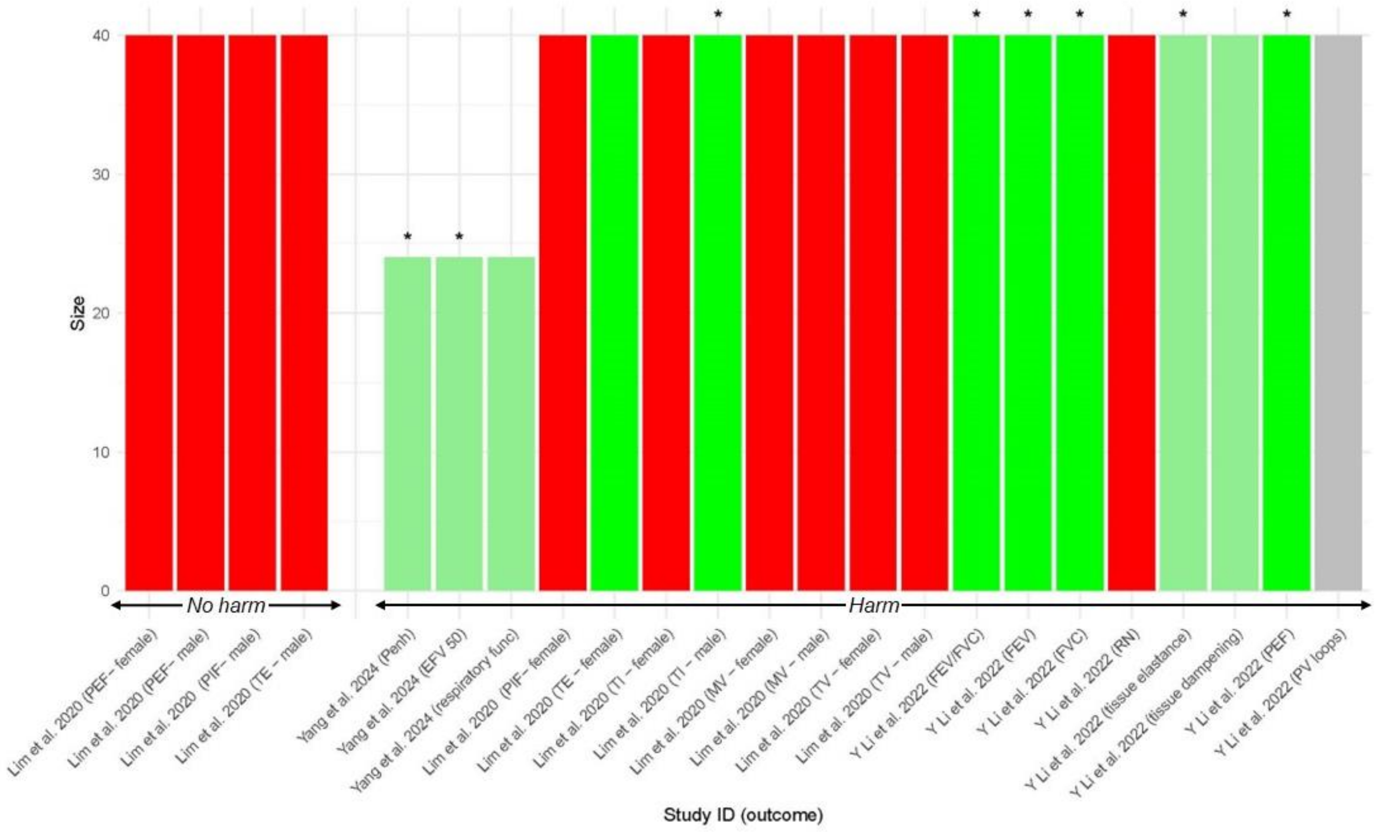

Key:

Direction of effect: Four study results show ‘no harm’, and 18 study results show change in the direction of ‘harm’

Y axis = sample size

Dark green = P<0.001, Green = P<0.01, Light green = ≤ 0.05, Red = > 0.05 Grey = P value is not calculated

*= Dose response identified in the study

Figure S8. Lung Injury

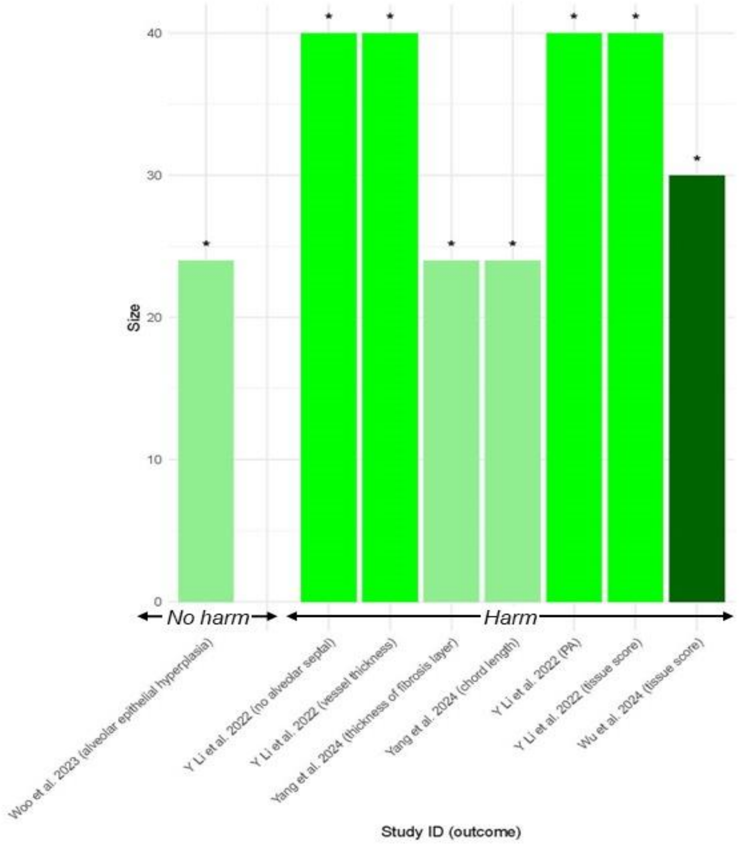

Key:
Direction of effect: One study result show ‘no harm’, and seven study results show change in the direction of ‘harm’.
Y axis = sample size
Y axis = sample size
Dark green = P<0.001, Green = P<0.01, Light green = ≤ 0.05, Red = > 0.05
*= Dose response identified in the study

Figure S9. Chronic inflammation

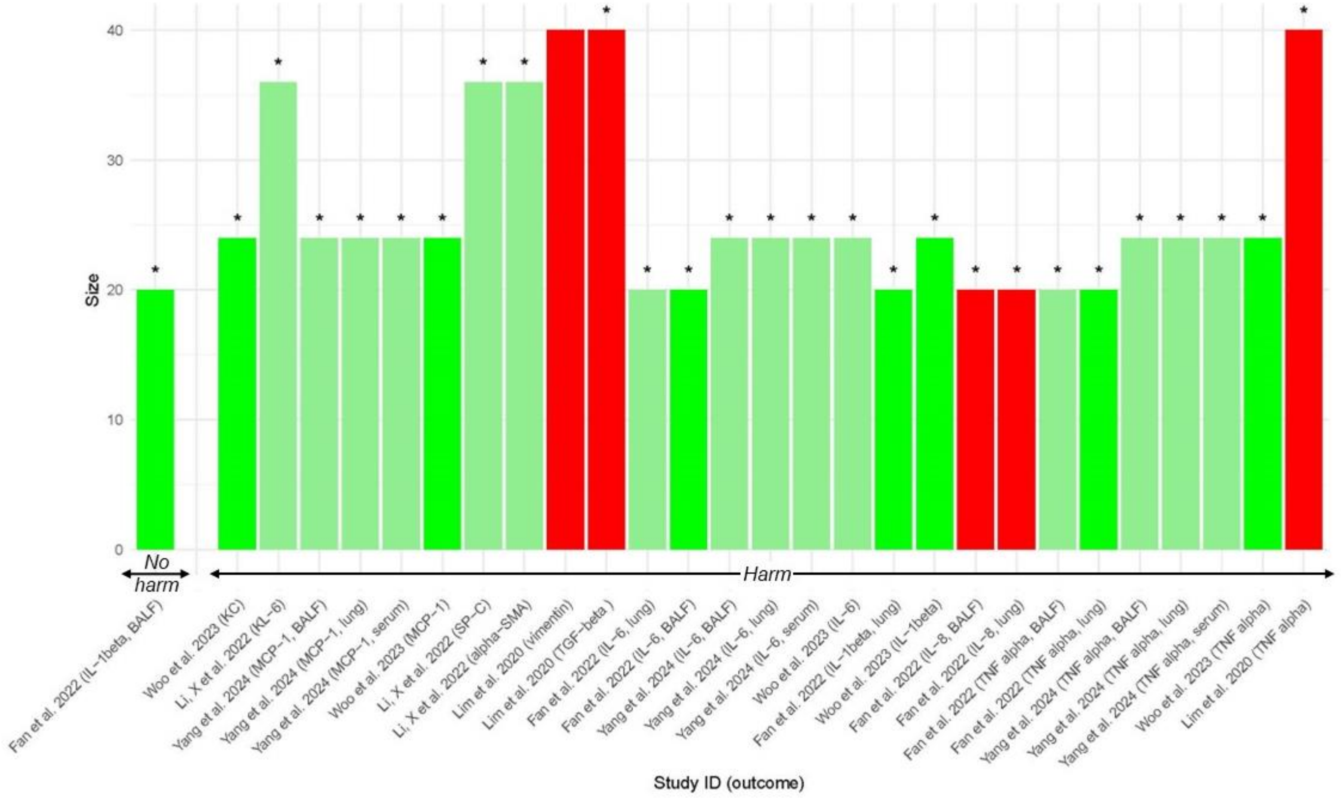

Key:

Direction of effect: One study result show 'no harm', and 27 study results show change in the direction of 'harm'.

Y axis = sample size

Dark green = P<0.001, Green = P<0.01, Light green = ≤ 0.05, Red = > 0.05

*= Dose response identified in the study

Figure S10. Oxidative stress

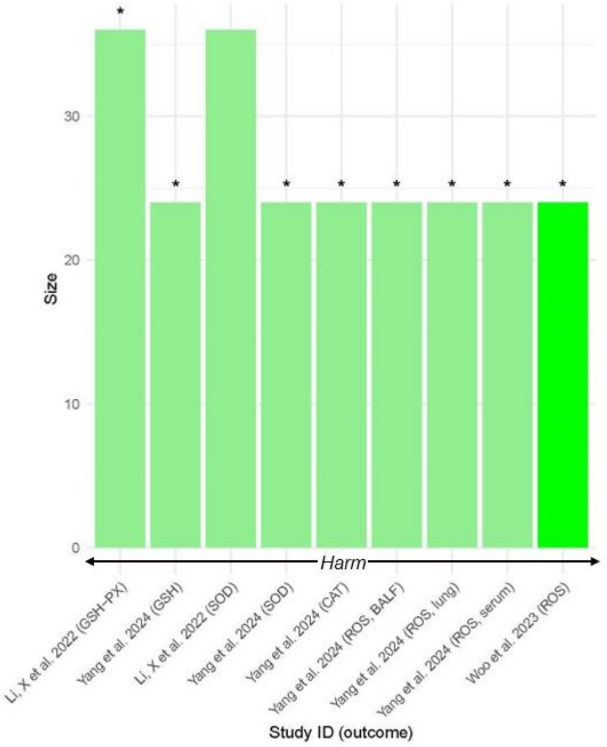

Key:

Direction of effect: All included study results show change in the direction of 'harm'.

Y axis = sample size

Dark green = P<0.001, Green = P<0.01, Light green = ≤ 0.05, Red = > 0.05

*= Dose response identified in the study
